# Integrated siRNA design based on surveying of features associated with high RNAi effectiveness

**DOI:** 10.1186/1471-2105-7-516

**Published:** 2006-11-27

**Authors:** Wuming Gong, Yongliang Ren, Qiqi Xu, Yejun Wang, Dong Lin, Haiyan Zhou, Tongbin Li

**Affiliations:** 1 Department of Neuroscience, University of Minnesota, Minneapolis, MN 55455, USA

## Abstract

**Background:**

Short interfering RNAs have allowed the development of clean and easily regulated methods for disruption of gene expression. However, while these methods continue to grow in popularity, designing effective siRNA experiments can be challenging. The various existing siRNA design guidelines suffer from two problems: they differ considerably from each other, and they produce high levels of false-positive predictions when tested on data of independent origins.

**Results:**

Using a distinctly large set of siRNA efficacy data assembled from a vast diversity of origins (the *siRecords *data, containing records of 3,277 siRNA experiments targeting 1,518 genes, derived from 1,417 independent studies), we conducted extensive analyses of all known features that have been implicated in increasing RNAi effectiveness. A number of features having positive impacts on siRNA efficacy were identified. By performing quantitative analyses on cooperative effects among these features, then applying a *disjunctive rule merging *(DRM) algorithm, we developed a bundle of siRNA design rule sets with the false positive problem well curbed. A comparison with 15 online siRNA design tools indicated that some of the rule sets we developed surpassed all of these design tools commonly used in siRNA design practice in positive predictive values (PPVs).

**Conclusion:**

The availability of the large and diverse siRNA dataset from *siRecords *and the approach we describe in this report have allowed the development of highly effective and generally applicable siRNA design rule sets. Together with ever improving RNAi lab techniques, these design rule sets are expected to make siRNAs a more useful tool for molecular genetics, functional genomics, and drug discovery studies.

## Background

Short interfering RNAs (siRNAs) are double-stranded RNAs typically of length between 19 and 25 with 2 nucleotide overhangs on the 3' ends, and they are capable of inducing sequence-specific, post-transcriptional deletion of gene products, leading to the silencing of the gene activity. Naturally occurring siRNAs are cleavage products from long double-stranded RNAs (dsRNAs) by Dicer, a ribonuclease III enzyme [1,2]. The siRNA-induced mRNA degradation is a complicated process involving multiple steps, initiated by the binding of siRNA with RISC (RNA induced silencing complex), followed by RISC's activation, resulting in the recognition of the target mRNA and the degradation of the latter [1,3,4]. As a gene knock-down tool used in labs, siRNAs can also be chemically synthesized and introduced into the cells by direct transfection [5,6] or delivered into the cells in forms of hairpin precursors through plasmid or viral vectors [7,8]. The siRNA-based gene knock-down techniques are preferred by many because of their ability to disrupt individual gene's function without affecting related genes [9]. These techniques are particularly attractive for gene silencing studies in mammalian cells, because, unlike longer double-stranded RNAs, siRNAs are not likely to trigger interferon responses which lead to non-specific mRNA degradation [5].

The efficacy issue represents a major challenge in siRNA design. This issue concerns the question of how to choose from the large number of candidate siRNAs the ones that give rise of the highest levels of knock-down activity. It is well known that only a fraction of these candidate siRNAs are highly effective in silencing the target genes. Two siRNAs targeting the mRNA sites that are separated by only a few nucleotides could exhibit very different knock-down efficacies [10,11]. What are the properties some siRNAs possess that render them more effective in knocking down the target genes than others? This is an issue of heated debate. Several sets of rules for designing high-efficacy siRNAs have been proposed (e.g., [11-14]). In addition, a long list of factors have been claimed to influence siRNA knock-down efficacy and thus should be considered in siRNA design [15-26].

There are significant disagreements among these design rules and considerable controversies over these claims. This situation has been discussed extensively in several recent review articles [27,28], therefore we only list some examples of these disagreements here: [20] suggested that the sequence information alone was sufficient in determining the efficacy of a siRNA; however, [15,22,24] advocated the need to incorporate thermodynamic properties (calculated using tools such as Mfold [29]) in assisting siRNA design; while [17,25] emphasized the importance of the accessibility to the mRNA sites by the siRNAs, and endorsed methods of filtering candidate siRNAs based on mRNA secondary structure properties. On factors determined by siRNA sequences, [12,30] recommended choosing of sequences of intermediate G/C contents (around 50%) for effective siRNAs, while [11,18,24,31,32] endorsed the choosing of sequences of lower G/C contents (< 60%) to increase the chance of making high-efficacy siRNAs. On position-specific properties, [11] suggested that the nucleotides on positions 3, 10, 13 and 19 on the sense strand played a critical role in determining the knock-down efficacy; while [14] claimed that positions 19 and 11, and perhaps 6, 13 and 16 on the sense strand were important in determining the knock-down efficacy of the siRNAs.

The debates over siRNA efficacy go beyond the disagreements among these design rules. In fact, the effectiveness of these rules *per se *is in question. [17] showed that most published siRNA design tools output large numbers of ineffective siRNAs, and had a similar performance to (or even worse than) a random selector when tested on data of an independent origin. [20] made similar observations, and alleged that several published efficacy predicting algorithms gave close to random classification on unseen data.

At least two groups of researchers pointed out that many existing studies on siRNA design criteria suffered from the "overfitting" problem [20,24]. This term describes scenarios where rules are extracted from datasets that have small sample sizes, low signal-to-noise ratios, and unique experimental settings. Rules obtained under these conditions are prone to spurious effects caused by noise in the data samples or specific aspects of the experimental settings or both; rules obtained in this manner are likely to perform unsatisfactorily when used on data obtained under different experimental settings.

The key to countering the overfitting problem and developing truly effective and generally applicable siRNA design rules is the availability of a large collection of siRNA efficacy data from diverse origins. We recently undertook the effort to document all siRNA experiments in published studies and provide sensible efficacy ratings of these experiments. This effort resulted in *siRecords*, the largest known curated database of mammalian siRNA experiments with consistent efficacy ratings [33]. The availability of the *siRecords *data makes it possible to better analyze factors responsible for achieving effective RNAi experiments.

In this study, we first conducted a survey on the *siRecords *data of all known "features" previously implicated to influence siRNA knock-down efficacy. This survey resulted in a list of features that significantly boosted the chance of achieving higher siRNA efficacies. Then, we examined quantitatively how these significant features interact with one another in their joint effects on achieving higher efficacies. The combinations of features that give rise to the highest levels of boosting to siRNA efficacies were picked and reorganized using a *disjunctive rule merging *(DRM) procedure, which led to a bundle of non-redundant rule sets with controlled stringency level. The performance of these rule sets (termed the *DRM rule sets*) was then assessed using a reserved dataset and compared with existing design tools commonly used in current siRNA design practice.

An implementation of the DRM rule sets developed in this study is available for testing as an online siRNA design server [34].

## Results

### Overview of siRecords data

*siRecords *is a continuing effort aimed to document all mammalian siRNA experiments reported in literature, and provide systematically rated efficacies for these experiments [33]. Currently, about 9000 records of siRNA experiments targeting more than 3000 genes are hosted in the *siRecords *database. For each siRNA experiment, we document the siRNA sequence, the target gene, key information about experimental conditions (cell line used; the method of producing the siRNA – chemically synthesized or vector-based; the method of testing the siRNA efficacy – western blot or real-time PCR or others), and an efficacy rating (elaborated below).

**Figure 1:**
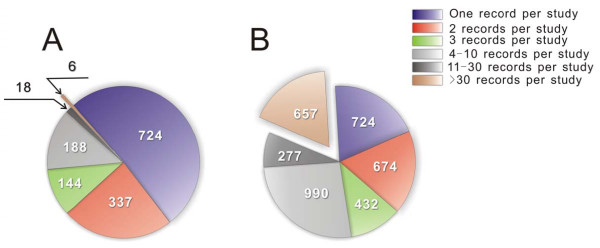
The distribution of the number of siRNA experiments per study is highly skewed in the *siRecords *collection. ***A. ***Studies were categorized based on the number of siRNA experiments reported. Only 6 out of the 1,417 studies (0.4%) reported > 30 siRNA experiments per study. ***B. ***The distribution of the total number of records in each category. Six hundred and fifty-seven records (representing 17.5% of the entire dataset) originated from the 6 studies with > 30 records per study.

For this investigation, we picked all complete records of 19-mer siRNA experiments (21-mers if the two overhanging nucleotides on the 3' ends are counted) from the *siRecords *collection (dated 12/12/2005). The distribution of number of records per study is highly skewed – about 17.5% of the records (657 siRNA experiments) originated from 0.4% of the studies (6 studies, each reporting ≥ 30 siRNA experiments, Figure 1). To prevent our analyses from being biased by this small number of studies, we limited the number of siRNA experiments originated from a single study to be ≤ 30. For these studies where more than 30 siRNA experiments were reported, we randomly picked 30 to include in our analyses and discarded the rest. The resulting dataset includes the records of 3277 siRNA experiments targeting 1518 genes originated from 1417 independent studies. We randomly divided the dataset into two subsets at a 2:1 ratio. The larger subset – termed Set A – included 2184 records, and was used to survey features significantly associated with high efficacies and analyze the combinatorial effects of these features. The other subset (termed Set T, 1093 records) was reserved to test the conclusions obtained through the analyses of Set A.

### Survey of features significantly boosting siRNA efficacy

We set out to determine, using the Set A data, what "features" of the siRNA experiments are associated with elevated RNAi efficacies. A *feature *is a binary property of a siRNA experiment concerning a factor potentially relevant to siRNA efficacy, for example, *the 6th nucleotide of the siRNA sequence = A*. Each feature has a "complementary feature". A feature and its complementary feature constitute a "feature pair". More discussions about the definition of feature and related terms can be found in Methods.

**Figure 2:**
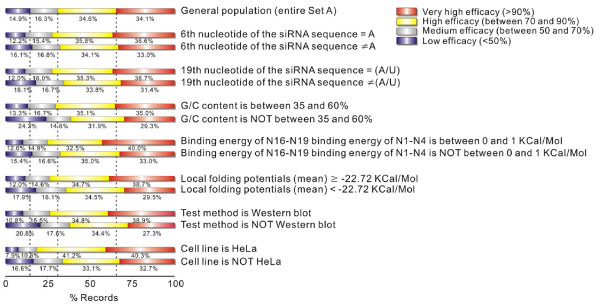
Survey of features associated with the achievement of higher efficacies. The efficacy of a siRNA experiment is rated on a four-level scale. In Set A, the percentages of records achieving these ratings are 34.1%, 34.6%, 16.3% and 14.9%, respectively. The distribution of the efficacy ratings across the four levels changes when certain feature is present in the siRNA experiments. For 14 selected features (they constitute 7 pairs of "complementary features"), the efficacy rating distributions of the subpopulations of siRNA experiments carrying these features are presented. Dotted vertical lines extend from the distribution of the general population.

In *siRecords*, the effectiveness of any siRNA experiment is rated on a four-level scale: very high (if the gene product was reduced by ≥ 90%), high (if the gene product was reduced by 70–90%), medium (if 50–70% knock-down was achieved); and low (if < 50% knock-down was observed). In Set A, the percentages of records receiving very high, high, medium and low efficacy ratings are 34.1%, 34.6%, 16.3% and 14.9% respectively (Figure 2). The decision of using this four-level rating scheme was made based on balanced considerations about the usefulness and the reliability of the ratings [33]. One consequence of this decision is that that the conventional t-test type of analysis [11] can not be performed on this dataset, because the dependent variable (efficacy rating) is not a continuous variable, but rather a categorical, ordinal variable. Proper categorical analysis techniques need to be adopted to analyze this type of data [35].

We chose to use the Wald test of monotone trend to assess the evidence that the presence of a feature is associated with a significant up-shift (or down-shift) of the efficacy distribution. In addition, we conducted odds ratio permutation tests for two efficacy levels: > 90% and > 70% efficacies, because in siRNA design practice, we are interested in assessing whether a feature leads to increased chances of achieving higher efficacies (see Methods). For instance, a Wald test of monotone trend indicated that the presence of the feature *the 6th nucleotide of the siRNA sequence = A *is associated with significant up-shift of the efficacy distribution (P = 0.0058); odds ratio permutation tests showed that the presence of this feature led to significant increase in the probabilities of achieving both > 90% (P = 0.043) and > 70% (P = 0.0024) efficacies (see Supplementary Figure 1 in Additional file 1).

We examined 276 features (they constitute 138 "feature pairs") for their association with higher RNAi efficacies, using the Wald test of monotone trend and the odds ratio permutation tests. The features we examined include, to our knowledge, all that have been implicated in previous studies to improve siRNA effectiveness. Each of these features can be placed into one of five categories. The first category is based on nucleotide identities at specific positions on the 19-mer siRNA sequence, e.g. *the 6th nucleotide = A*; there are 76 feature pairs in this category. The second category includes 19 feature pairs that are either composite sequence features, e.g. *there are at least three (A/U)'s in the seven nucleotides at the 3' end of the siRNA*, or features that are defined based on the G/C content of the siRNA. The third category consists of 13 feature pairs that are based on the thermodynamics of the siRNAs as measured by the melting temperature, or binding energy. The fourth category, consisting of 16 feature pairs, includes features based on target mRNA sites, such as the relative positions of the target sites on the mRNA, and the local secondary structures of the target regions. Finally, the fifth category includes 14 feature pairs that are based on experimental settings, such as the cell lines used in the experiments (HeLa cells, HEK293 cells, and others), the methods used for making and delivering the siRNAs, and the methods used to evaluate the efficacy of the siRNA (Western blot, PCR-based, and others). The complete list of these tested features, and references to the studies that implicated them in enhancing siRNA efficacies, are provided in Supplementary Tables 1-5 in Additional file 1.

**Table 1:** Non-redundant significant features meeting the criteria (*P*_*wald *_< 0.01) and (*P*_*70 *_< 0.01 or *P*_*90 *_< 0.01)

**Feature name**	**% Low**	**% Medium**	**% High**	**% Very high**	***P*_*70*_**	***P*_*90*_**	***P*_*wald*_**
2nd nucleotide = A	12.1	16.0	33.8	38.1	0.01	0.0026	0.0019
4th nucleotide = C	14.1	15.4	31.5	39.0	0.098	0.00036	0.0075
6th nucleotide ≠ C	14.3	15.6	35.0	35.1	0.00066	0.0089	0.0052
7th nucleotide ≠ U	14.4	15.9	34.5	35.2	0.01	0.0043	0.0091
9th nucleotide = C	11.1	16.6	32.6	39.6	0.008	0.00021	0.00053
17th nucleotide = A	11.4	15.5	37.1	35.9	0.00049	0.1	0.0049
18th nucleotide ≠ C	14.4	15.9	34.3	35.4	0.01	0.00071	0.0048
19th nucleotide = (A/U)	12.0	16.0	35.3	36.7	0.00029	0.0043	0.000058
At least three (A/U)s in the seven nucleotides at the 3' end	13.4	16.4	33.7	36.5	0.00001	0.00001	2.5E-09
No occurrences of four or more identical nucleotides in a row	14.2	15.9	35.4	34.5	0.00001	0.012	0.0014
No occurrences of G/C stretches of length 7 or longer	14.3	16.4	34.9	34.4	0.00001	0.00001	0.000015
G/C content is between 35% and 60%	13.3	16.7	35.1	35.0	0.00001	0.0019	0.00018
T_m _is between 20 and 60°C	13.2	16.5	35.0	35.3	0.0045	0.023	0.003
Binding energy of N16–N19 > -9 KCal/Mol	11.8	17.1	34.0	37.1	0.01	0.0026	0.00025
Binding energy of N16–N19 – binding energy of N1–N4 is between 0 and 1 KCal/Mol	12.6	14.9	32.5	39.9	0.01	0.00036	0.0078
Local folding potential (mean) ≥ -22.72 KCal/Mol	12.0	14.6	34.7	38.7	0.00001	0.00001	9.3E-09
Target site is on CDS	14.4	16.2	34.3	35.2	0.00001	0.00001	0.000055
Cell line = HeLa	7.9	10.6	41.2	40.3	0.00001	0.00016	4.0E-09
Test method = Western blot	10.8	15.5	34.8	36.9	0.00001	0.00001	3.8E-14
Test object ≠ mRNA	13.1	14.5	34.8	37.6	0.00001	0.00001	9.3E-10

At P < 0.01, the FDR for the three tests: Wald test of monotone trend, permutation test of odds ratios (> 70%) and permutation test of odds ratios (> 90%) were controlled at the levels of 0.056, 0.044 and 0.038 respectively.

**Table 2:** Non-redundant DRM rule set for the highest *α *level:*RS*_*0.951*_.

**Feature**	**F**_1_	**F**_2_	**F**_3_	**F**_4_	**F**_5_	**F**_6_	**F**_7_	**F**_8_	**F**_9_	**F**_10_	**F**_11_	**F**_12_	**F**_13_	**F**_14_	**F**_15_	**F**_16_	**F**_17_
Rule 1	√	√			√										√		
Rule 2		√			√			√						√	√		
Rule 3	√	√				√									√	√	√
Rule 4		√			√		√	√							√	√	
Rule 5	√				√								√	√	√	√	
Rule 6		√			√	√	√	√							√		
Rule 7		√			√		√	√					√		√		

**List of features:**																	

**Feature Index**	**Feature Names**																

F_1_	2nd nucleotide = A																
F_2_	4th nucleotide = C																
F_3_	6th nucleotide ≠ C																
F_4_	7th nucleotide ≠ U																
F_5_	9th nucleotide = C																
F_6_	17th nucleotide = A																
F_7_	18th nucleotide ≠ C																
F_8_	19th nucleotide = (A/U)																
F_9_	At least three (A/U)s in the seven nucleotides at the 3' end																
F_10_	No occurrences of four or more identical nucleotides in a row																
F_11_	No occurrences of G/C stretches of length 7 or longer																
F_12_	G/C content is between 35 and 60%																
F_13_	T_m _is between 20 and 60°C																
F_14_	Binding energy of N16–N19 > -9 KCal/Mol																
F_15_	Binding energy of N16–N19 – binding energy of N1–N4 is between 0 and 1 KCal/Mol																
F_16_	Local folding potential (mean) ≥ -22.72 KCal/Mol																
F_17_	Target site is on CDS																

**Table 3:** Comparison in performance between 15 online siRNA design tools and DRM rule sets with four different stringency levels (*α *= 0.951, 0.895, 0.845 and 0.827).

**Design Program**	**Institution/Company**	**URL**	**Avg. # Effective siRNAs** **Predicted per Gene**	**Sensitivity** **(> 90%)**	**Specificity** **(> 90%)**	***PPV*** ***(> 90%)***	**Sensitivity** **(> 70%)**	**Specificity** **(> 70%)**	***PPV (> 70%)***
Ambion siRNA Target Finder	Ambion, Inc.	[64]	190.0	0.603	0.456	***37.0%***	0.574	0.457	***70.3%***
Jack Lin's siRNA Sequence Finder	Cold Spring Harbor Laboratory	[65]	207.5	0.204	0.759	***30.9%***	0.221	0.757	***67.1%***
siDESIGN Center	Dharmacon, Inc.	[66]	9.8	0.042	0.976	***48.5%***	0.036	0.982	***81.8%***
siRNA Target Finder	GenScript Corp.	[67]	22.4	0.032	0.979	***44.4%***	0.030	0.988	***85.2%***
Imgenex sirna Designer	Imgenex Corp.	[68]	22.8	0.116	0.913	***41.5%***	0.108	0.929	***77.4%***
EMBOSS sirna	Institute Pasteur	[69]	639.4	0.778	0.250	***35.4%***	0.767	0.258	***69.9%***
IDT RNAi Design (SciTools)	Integrated DNA Technologies, Inc.	[70]	5.8	0.032	0.975	***40.0%***	0.030	0.979	***76.7%***
BLOCK-iT RNAi Designer	Invitrogen Corp.	[71]	11.4	0.026	0.985	***47.6%***	0.020	0.982	***71.4%***
siSearch	Karolinska Institutet	[72]	19.6	0.016	0.986	***37.5%***	0.017	0.991	***81.3%***
SiMAX	MWG-Biotech, Inc.	[73]	35.1	0.161	0.843	***35.3%***	0.172	0.872	***75.1%***
BIOPREDsi	Novartis Institutes for BioMedical Research	[74]	10.0	0.794	0.908	***31.3%***	0.820	0.899	***64.6%***
Promega siRNA Target Designer	Promega Corp.	[75]	38.0	0.093	0.941	***45.5%***	0.083	0.958	***81.8%***
QIAGEN siRNA Design Tool	QIAGEN, Inc.	[76]	29.6	0.167	0.862	***38.9%***	0.161	0.881	***75.3%***
SDS/MPI	University of Hong Kong	[77]	432.8	0.656	0.380	***35.9%***	0.632	0.368	***69.2%***
WI siRNA Selection Program	Whitehead Institute	[78]	9.5	0.019	0.992	***53.8%***	0.015	0.994	***84.6%***
**DRM *RS*_*0.951*_**			**18.9**	**0.021**	**0.996**	***72.7%***	**0.013**	**0.997**	***90.9%***
**DRM *RS*_*0.895*_**			**20.7**	**0.032**	**0.992**	***66.7%***	**0.021**	**0.994**	***88.9%***
**DRM *RS*_*0.845*_**			**38.1**	**0.032**	**0.986**	***54.5%***	**0.026**	**0.984**	***90.9%***
**DRM *RS*_*0.827*_**			**51.8**	**0.037**	**0.973**	***42.4%***	**0.038**	**0.988**	***87.9%***

Comparison made based on Set T (1,093 siRNA experiments targeting 744 genes). Default settings were used for the 15 online predicting tools. Two sets of parameters (sensitivity, specificity and PPV) were calculated for each predicting tool or rule set. One was for > 90% efficacy (that is, a siRNA experiment was considered as truly effective if it achieved > 90% efficacy), the other one was for > 70% (considered truly effective if > 70% efficacy was reached in the experiment).

**Table 4:** Comparison in performance between 15 online siRNA design tools and 4 DRM rule sets based on independent subset of Set T.

**Design Program**	**Institution/Company**	**# Predicted effective siRNAs**	**# Predicted ineffective siRNAs**	**Sensitivity**	**Specificity**	***PPV (%)***
Ambion siRNA Target Finder	Ambion, Inc.	144	80	0.645	0.362	*74.3*
Jack Lin's siRNA Sequence Finder	Cold Spring Harbor Laboratory	44	180	0.229	0.897	*86.4*
siDESIGN Center	Dharmacon, Inc.	7	217	0.036	0.983	*85.7*
siRNA Target Finder	GenScript Corp.	6	218	0.024	0.966	*66.7*
Imgenex sirna Designer	Imgenex Corp.	24	200	0.114	0.914	*79.2*
EMBOSS sirna	Institute Pasteur	180	44	0.801	0.190	*73.8*
IDT RNAi Design (SciTools)	Integrated DNA Technologies, Inc.	4	220	0.012	0.966	*50.0*
BLOCK-iT RNAi Designer	Invitrogen Corp.	2	222	0.012	1.000	*100*
siSearch	Karolinska Institutet	0	224	N/A	N/A	*N/A*
SiMAX	MWG-Biotech, Inc.	48	176	0.235	0.845	*81.3*
BIOPREDsi	Novartis Institutes for BioMedical Research	4	220	0.018	0.983	*75.0*
Promega siRNA Target Designer	Promega Corp.	26	198	0.127	0.914	*80.8*
QIAGEN siRNA Design Tool	QIAGEN, Inc.	33	191	0.151	0.862	*75.8*
SDS/MPI	University of Hong Kong	151	73	0.663	0.293	*72.8*
WI siRNA Selection Program	Whitehead Institute	12	212	0.072	1.000	*100*
**DRM *RS*_*0.951*_**		**1**	**223**	**0.006**	**1**	***100***
**DRM *RS*_*0.895*_**		**4**	**220**	**0.024**	**1**	***100***
**DRM *RS*_*0.845*_**		**5**	**219**	**0.030**	**1**	***100***
**DRM *RS*_*0.827*_**		**9**	**215**	**0.048**	**0.983**	***88.9***

Comparison made based on the independent subset of Set T (224 siRNA experiments targeting 197 genes). Default settings were used for the 15 online predicting tools. A siRNA experiment was considered effective if it achieved > 70% efficacy (was rated "high" or "very high" efficacy).

**Table 5:** Contingency table for the outcome of prediction tasks.

	**Truly Effective**	**Truly Ineffective**
**Predicted Effective**	*N*_*A*_	*N*_*B*_
**Predicted Ineffective**	*N*_*C*_	*N*_*D*_

Of the features examined, we found 34 that were associated with a significant improvement in the efficacy distribution (P < 0.01, Wald test of monotone trend; FDR controlled at 0.056 by the q-value technique [36]); among which, 26 significantly elevated the chance of achieving > 90% efficacies (P < 0.01, odds ratio permutation test, FDR controlled at 0.038), and 27 significantly enhanced the probability of achieving > 70% efficacies (P < 0.01, odds ratio permutation tests, FDR controlled at 0.044; see Supplementary Tables 1-5  in Additional file 1). There are several cases of sub-feature – super-feature relationships among these significant features. For example, the features *the 6th nucleotide = A*, and *the 6th nucleotide *≠ *C *were both significant features, however, the former is a sub-feature of the latter since when the former feature is present, the latter must also be present. In each occurrence of sub-feature – super-feature relationship, we eliminated all but the one feature determined to be the most significant by the Wald test. The feature *the 6th nucleotide = A *was thus eliminated because the Wald test P value of this feature was higher than that of the feature *the 6th nucleotide *≠ *C*. G/C content related features were treated as a special case. Several different G/C content ranges were suggested in previous studies as being possibly associated with high RNAi effectiveness (32–79%, 30–70%, 30–52%, 35–60%, 20–50% and 31.6–57.9%) [11,12,18,24,30-32]. All these features were tested. Although they do not constitute sub-feature – super-feature relationships, we treated these features as redundant features, and retained only one of them (*G/C content is between 35 and 60%*) because it yielded the lowest P value (0.00018) in the Wald test. The resulting list of non-redundant significant features is shown in Table 1. Detailed discussions about these significant features, and comparisons of our analyses with previous findings can be found in the Additional file 1.

### Combined effects of multiple significant features

The presence of any single significant feature was not sufficient to improve the efficacy distribution substantially. When present alone, the significant features listed in Table 1 increased the probability of achieving > 90% efficacies by an average of only 2.5% (from 34.1% to 36.6%), and they increased the chance of achieving > 70% efficacies by an average of merely 2.2% (from 68.7% to 70.9%). To achieve substantially improved efficacies, the concurrent presence of several significant features is required.

When multiple features are co-present, we cannot assume that their contributions to the effectiveness of the RNAi experiments are additive, since features are not always independent of one another. For instance, the presence of the feature *the 19th nucleotide = (A/U)*, clearly increases the probability that the feature *there are at least three (A/U)'s in the seven nucleotides on the 3' end of the siRNA *to be true. Indeed, these two features exhibited negative cooperativity: when present alone, they increased the chances of achieving > 90% efficacies by 2.6% and 2.4%, respectively; when co-present, these two features resulted in merely a 2.7% increase in the chance of achieving > 90% efficacy, much smaller than the sum of the effects of the two features (see Additional file 1 for discussions about cooperativity and additive effects of multiple features).

**Figure 3:**
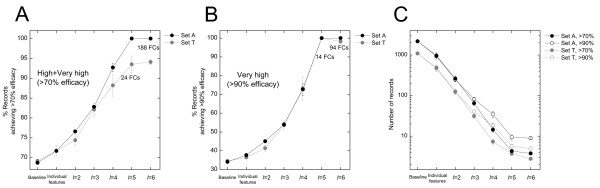
Highly effective siRNA design rules were obtained by selecting the top *l*-feature combinations, i.e., the combination of *l *non-redundant significant features that exhibited the highest percentages of records achieving > 70% or > 90% efficacies on Set A. ***A. ***For *l *= 2 through 6, the subpopulations of Set A records that carry all combinations of *l *features were examined, and the 10 feature combinations (FCs) that resulted in the highest percentages of records achieving > 70% efficacies were selected. When there was a tie of more than 10 FCs, all of them were considered (marked in the graph). The mean percentages of the top FCs are presented in black filled circles. These FCs were used to select siRNA experiments in the Set T, and the results are shown in grey filled circles. Error bars indicate standard errors. The first two data points in the graphs represent the base line levels (the percentage of records achieving > 70% efficacies for the entire Set A or Set T), and the mean levels for top-10 individual features (the 10 individual features that led to highest percentages of records achieving > 70% efficacies), respectively. ***B. ***Similarly to ***A***, the top FCs selected with > 90% efficacies are plotted, together with the baseline levels and the mean levels for top individual features. ***C. ***The numbers of records selected in the top *l*-feature combinations dropped sharply as *l *increased. The mean numbers of selected records for Set A (with error bars indicating standard errors) are presented in black filled circles and black open circles for > 70% and > 90% efficacies, respectively. The numbers of selected records for Set T are presented in corresponding grey symbols. Again, the first two data points represent the baseline levels (numbers of records in entire Set A and Set T), and the numbers of records selected with the top-10 individual features, respectively.

In seeking effective siRNA design rules, we should try to identify combinations of features that exhibit positive cooperativity. The large size and diverse origins of the records in the *siRecords *dataset allowed us to systematically analyze how features jointly influence siRNA efficacies. Three significant features: *Cell line = HeLa*, *Test method = Western blot *and *Test object *≠ *mRNA *were excluded from joint effect analyses because they are based on experimental settings, which are typically chosen independent of siRNA design. For the remaining 17 significant features, we looked at all possible combinations of a fixed number (*l *= 2,3,4,5 and 6) of features. For each combination of *l *features, we examined the number of records in Set A that concurrently carry all *l *features, and the percentages of these records that achieved > 90% and > 70% efficacies. For every given *l*, we focused on the top-10 feature combinations, i.e., the 10 combinations that exhibited the highest percentage of records achieving > 90% or > 70% efficacies. When there was a tie of more than 10 feature combinations, all tied combinations were considered. As we expected, as *l *– the number of features in the combinations increased, the number of records concurrently carrying all *l *features declined sharply (Figure 3C). Meanwhile, the percentage of experiments achieving > 90% and > 70% efficacies increased steadily as *l*, the number of features included in the feature combinations, increased (Figure 3A and 3B).

The sigmoid shape of the two ascending curves is an indication of positive cooperativity (see discussion in Additional file 1). This suggests that by simply retaining the feature combinations that led to the highest percentages of records achieving efficacies of > 90% or > 70%, we were, in effect, exploiting the positive cooperativity, or favorable interaction, among these features. At *l *= 5, 24 feature combinations had a 100% chance of having efficacies > 70%, that is, every experiment in which the siRNA used had all features contained in any one of the 24 feature combinations exhibited efficacies of > 70%. Similarly, 14 feature combinations had 100% probabilities of having efficacies > 90% at *l *= 5, meaning that all siRNA experiments having these feature combinations demonstrated efficacies > 90%. At *l *= 6, 188 feature combinations had 100% probabilities of having efficacies of > 70%, and 94 feature combinations had 100% probabilities of achieving efficacies of > 90%.

### Integrated rule sets for effective siRNA design

A disjunction of the top feature combinations described above (across *l *= 2 through 6; a feature combination is also called a *rule *thereafter) defines a *rule set *for designing effective siRNA experiments. Rule sets defined in this way are likely to contain redundancies, because if a rule consisting of l^
 MathType@MTEF@5@5@+=feaafiart1ev1aaatCvAUfKttLearuWrP9MDH5MBPbIqV92AaeXatLxBI9gBaebbnrfifHhDYfgasaacH8akY=wiFfYdH8Gipec8Eeeu0xXdbba9frFj0=OqFfea0dXdd9vqai=hGuQ8kuc9pgc9s8qqaq=dirpe0xb9q8qiLsFr0=vr0=vr0dc8meaabaqaciaacaGaaeqabaqabeGadaaakeaacuWGSbaBgaqcaaaa@2E1D@ features {*f*_1_, *f*_2_,..., fl^
 MathType@MTEF@5@5@+=feaafiart1ev1aaatCvAUfKttLearuWrP9MDH5MBPbIqV92AaeXatLxBI9gBaebbnrfifHhDYfgasaacH8akY=wiFfYdH8Gipec8Eeeu0xXdbba9frFj0=OqFfea0dXdd9vqai=hGuQ8kuc9pgc9s8qqaq=dirpe0xb9q8qiLsFr0=vr0=vr0dc8meaabaqaciaacaGaaeqabaqabeGadaaakeaacqWGMbGzdaWgaaWcbaGafmiBaWMbaKaaaeqaaaaa@2F9E@} is one of the best l^
 MathType@MTEF@5@5@+=feaafiart1ev1aaatCvAUfKttLearuWrP9MDH5MBPbIqV92AaeXatLxBI9gBaebbnrfifHhDYfgasaacH8akY=wiFfYdH8Gipec8Eeeu0xXdbba9frFj0=OqFfea0dXdd9vqai=hGuQ8kuc9pgc9s8qqaq=dirpe0xb9q8qiLsFr0=vr0=vr0dc8meaabaqaciaacaGaaeqabaqabeGadaaakeaacuWGSbaBgaqcaaaa@2E1D@ -feature combinations, then a rule consisting of (l^
 MathType@MTEF@5@5@+=feaafiart1ev1aaatCvAUfKttLearuWrP9MDH5MBPbIqV92AaeXatLxBI9gBaebbnrfifHhDYfgasaacH8akY=wiFfYdH8Gipec8Eeeu0xXdbba9frFj0=OqFfea0dXdd9vqai=hGuQ8kuc9pgc9s8qqaq=dirpe0xb9q8qiLsFr0=vr0=vr0dc8meaabaqaciaacaGaaeqabaqabeGadaaakeaacuWGSbaBgaqcaaaa@2E1D@+1) features {*f*_1_, *f*_2_,..., fl^
 MathType@MTEF@5@5@+=feaafiart1ev1aaatCvAUfKttLearuWrP9MDH5MBPbIqV92AaeXatLxBI9gBaebbnrfifHhDYfgasaacH8akY=wiFfYdH8Gipec8Eeeu0xXdbba9frFj0=OqFfea0dXdd9vqai=hGuQ8kuc9pgc9s8qqaq=dirpe0xb9q8qiLsFr0=vr0=vr0dc8meaabaqaciaacaGaaeqabaqabeGadaaakeaacqWGMbGzdaWgaaWcbaGafmiBaWMbaKaaaeqaaaaa@2F9E@, *f*_0_}, where *f*_0 _is any other feature, is likely to be one of the best (l^
 MathType@MTEF@5@5@+=feaafiart1ev1aaatCvAUfKttLearuWrP9MDH5MBPbIqV92AaeXatLxBI9gBaebbnrfifHhDYfgasaacH8akY=wiFfYdH8Gipec8Eeeu0xXdbba9frFj0=OqFfea0dXdd9vqai=hGuQ8kuc9pgc9s8qqaq=dirpe0xb9q8qiLsFr0=vr0=vr0dc8meaabaqaciaacaGaaeqabaqabeGadaaakeaacuWGSbaBgaqcaaaa@2E1D@+1)-feature combinations thus is also selected into the rule set. A *disjunctive rule merging *(DRM) algorithm can be applied to remove redundancies of the rule sets, in the mean time allowing the control over the stringency of the resulting rule sets (see Methods). This algorithm takes in a user-provided stringency parameter *α *(which has a range of [0, 1]), and produces a non-redundant set of disjunctive rules, each rule in the set resulting in ≥ *α *proportion of the records in Set A reaching efficacies > 90%. The rule set rendered for the highest *α *level (*α *= 0.951, denoted as *RS*_*0.951*_) contains seven rules (Table 2). Generally speaking, the lower *α *level, the larger number of rules are included in the rule set (see Supplementary Table 6 in Additional file 1).

### Performance comparison between DRM rule sets and existing design tools

**Figure 4:**
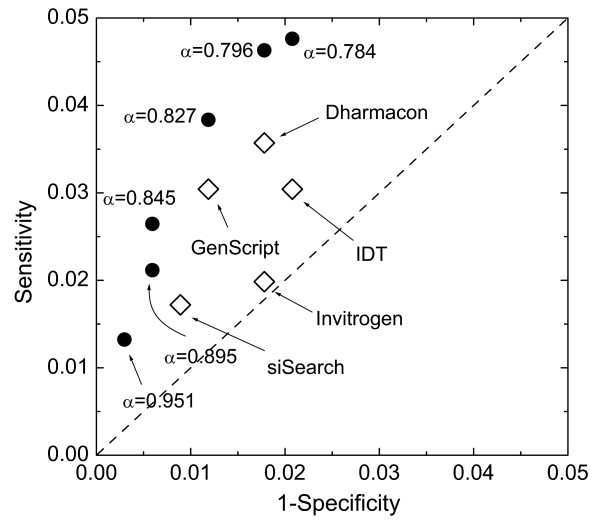
The ROC graph shows the performance the DRM rule sets of several *α *levels (filled circles) and that of several existing online predicting tools (open diamonds, "Dharmacon" denotes Dharmacon Inc.'s siDesign Center, "GenScript" denotes GenScript Corp.'s siRNA Target Finder, "IDT" denotes Integrated DNA Technologies Inc.'s RNAi Design (SciTools), "Invitrogen" denotes Invitrogen Corp.'s BLOCK-iT RNAi Designer, and "siSearch" stands for the siSearch tool by CGB, Karolinska Institutet). A siRNA experiment was considered effective if it achieved > 70% efficacy (was rated "high" or "very high" efficacy). The dotted line denotes the diagonal of the ROC. Unlike the diagonal line in a ROC of a common training task which represents the performance of a random guesser, the diagonal line shown in this graph represents the general siRNA design practice, because this is where the *siRecords *data were obtained. Symbols to the left-upper side of the diagonal line represent design rules that perform better than the general design practice. The farther away a symbol is from the dotted line, the better performance the corresponding design tool presents.

We assessed the performance of the DRM rule sets, and compared it with that of 15 existing online design tools commonly used in siRNA design practice, using the Set T data reserved for this purpose (Table 3 and Figure 4). Set T includes the records of 1,093 siRNA experiments, representing 1,014 unique target sites on 744 genes. How do we assess the performance of a siRNA design program? A good siRNA design program should (*a*) provide a sufficient number of candidate siRNAs for a given gene; and (*b*) offer a high PPV (positive predictive value), or a low false positive rate (see Methods).

On the number of candidate siRNAs predicted, the DRM rule set with the highest stringency level (*RS*_*0.951*_) produced on average 18.9 predicted effective siRNAs per gene. This indicates that this rule set offers sufficient candidate siRNAs in an ordinary siRNA design task for a gene of an average length. However, the smallest number of predicted effective siRNAs for a gene is 1. This suggests that for genes of the shortest lengths, the number of candidate siRNAs offered by this rule set may not be enough. There are considerations other than achieving high efficacy (e.g., avoiding cross-reactivity with other genes) in the design of siRNA experiments, thus it is desirable to have multiple candidate siRNAs designed for every gene. For genes of the shortest lengths, we resort to DRM rule sets of lower stringency levels. For example, *RS*_*0.845 *_produced at least 3 potentially effective siRNAs for each gene, and an average of 38.1 potentially effective siRNAs per gene (see Supplementary Figure 3 in Additional file 1). The online design tools varied greatly in the numbers of candidate siRNAs they provided. The highest number of predicted effective siRNAs was offered by EMBOSS sirna by Institute Pasteur (639.4 siRNAs per gene). IDT RNAi Design by IDT, Inc. produced the lowest number of predicted effective siRNAs (5.8 siRNAs per gene). Among the 15 online design tools, 10 offered larger numbers of candidate siRNAs than DRM *RS*_*0.951*_, and 4 provided larger numbers of candidate siRNAs than DRM *RS*_*0.845*_.

Given that a sufficient number of candidate siRNAs are provided, the most important parameter that measures the performance of a design tool is the PPV. Only a small proportion of possible siRNA sites have been experimentally tested for effectiveness (1,014 sites among 2,453,510 possible 19-mer sites on the 744 genes). Based on these experimentally tested siRNA sites, we compared the PPVs of the DRM rule sets to those of 15 existing online design tools. For the "> 90% efficacy" setting and "> 70% efficacy" setting, DRM *RS*_*0.951 *_showed PPVs of 72.7% and 90.9%, respectively. In other words, 72.7% of the predicted effective siRNAs by DRM *RS*_*0.951 *_had > 90% efficacy, and 90.9% of the predicted effective siRNAs showed > 70% efficacy. This rule set and two others with lower *α *level, *RS*_*0.895 *_and *RS*_*0.845 *_surpassed all online design tools in PPVs on both settings. Among the 15 online design tools, the three that offered the highest PPVs for the "> 90% efficacy" setting were WI siRNA Selection Program by Whitehead Institute (53.8%), siDESIGN Center by Dharmacon Inc. (48.5%) and BLOCK-iT RNAi Designer by Invitrogen Corp. (47.6%), respectively; and the four that offered the highest PPVs for the "> 70% efficacy" setting were siRNA Target Finder by GenScript Corp. (85.2%), WI siRNA Selection Program by Whitehead Institute (84.6%), siDESIGN Center by Dharmacon. Inc. (81.8%) and siRNA Target Designer by Promega Corp. (81.8%), respectively.

Set T is a fair dataset to be used for the purpose of performance comparison between the DRM rule sets and the online design tools, because it contains no overlapping records with Set A, based on which the DRM rule sets were derived. However, Set T might not be considered as a completely independent dataset, because (*a*), there are records in Set T that originated from the same studies as some records in Set A; and (*b*), there are records of siRNA experiments in Set T that target the same genes as some experiments in Set A. To rule out the possibility that these two factors might contribute to better performance of the DRM rule sets for unforeseen reasons and unfairly favor the DRM rule sets in the performance comparison, we compiled an "independent subset" of Set T, eliminating all records that share the same origins of any records in Set A, and all records that target the same genes that are also targeted by any records in Set A. We compared the performance of the DRM rule sets with that of the 15 online design tools using this independent subset (including 224 siRNAs targeting 197 different genes, see Table 4). Because of the reduced size of the dataset (by nearly 80%), the sensitivity, specificity and PPVs for all tools and rule sets showed higher levels of variability. The three DRM rule sets with the highest *α *levels: *RS*_*0.951*_, *RS*_*0.895 *_and *RS*_*0.845 *_achieved 100% PPV. Two online design tools, BLOCK-iT by Invitrogen Corp. and WI siRNA Selection Program by Whitehead Institute also achieved 100% PPV, but the other online design tools achieved lower PPVs that range between 50.0% and 86.4%. Although the small size of the independent subset prevented this analysis from being completely conclusive, it is fair to state that the comparison made based on the independent subset is generally in agreement with the comparison made based on the entire Set T.

## Discussion

It has been recognized that many existing siRNA design criteria (and the design tools in which they are implemented) failed to provide promised levels of performance when tested with unseen data largely due to the "overfitting" problem in their development [20,24]. Practically, the key to countering this problem is to make use of a large siRNA efficacy data from diverse origins when developing siRNA design rules. In this study, we took advantage of the recent *siRecords *collection in our development of the DRM rule sets. First, we conducted a survey on the *siRecords *dataset of all known "features" previously implicated to influence siRNA knock-down efficacy. This survey resulted in a list of features that significantly boosted the chance of achieving higher siRNA efficacies. Then, we examined quantitatively how these significant features interact with one another in their joint effects on achieving higher efficacies. The combinations of features that give rise to the highest levels of boosting to siRNA efficacies were picked and reorganized using the DRM algorithm, producing the rule sets. Finally, the performance of these rule sets was verified on a reserved dataset (Set T, also from *siRecords*) and was compared with that of 15 online siRNA design tools commonly used in current siRNA design practice.

The survey of features influencing RNAi effectiveness conducted in this study is the largest scale survey of this type ever reported by far (276 features were examined on a siRNA efficacy dataset consisted of 2,184 records of experiments originated from 1,141 independent studies). Among the significant features identified in the survey (Table 1) are several that have been implicated in multiple previous studies to influence the siRNA efficacy. They include a few features related to weaker binding on the 3' end (*the 17th nucleotide = A, the 18th nucleotide ≠ C, the 19th nucleotide = (A/U), At least three (A/U)s in the seven nucleotides at the 3' end, Binding energy of N16–N19 > -9 KCal/Mol*, and *Binding energy of N16–N19 – binding energy of N1–N4 is between 0 and 1 KCal/Mol*), one feature about a lower G/C range (*G/C content is between 35% and 60%*), two features related to unusual sequence patterns (*No occurrences of four or more identical nucleotides in a row *and *No occurrences of G/C stretches of length 7 or longer*), one feature related to melting temperature (*T*_*m *_*is between 20 and 60°C*), and one feature related to the target location (*Target site is on CDS*). However, there are also a small number of features that were not reported to be significant in any previous studies, e.g., *the 4th nucleotide = C *and *the 9th nucleotide = C*. It appears that there are higher levels of disagreements for sequence related features (Categories 1 and 2) than for features defined based on thermodynamics of the siRNAs and on target mRNA sites (Categories 3 and 4) between our survey results and previous findings, with the exception of the 3-nucleotide segment on the 3' end (N17–N19, the lower G/C content in this segment is correlated to lower binding energy on the 3' end). Notably, three Category 5 features (defined based on experimental settings) *Cell line = HeLa*, *Test method = Western blot *and *Test object *≠ *mRNA *were among those found to be most significant. Although there have been reports about siRNA efficacy being influenced by cell lines and test methods [37-40], this is the first quantitative analysis about how strong these influences are. More details about the significant features found in the survey, and comparisons of our analyses with previous findings are presented in the Additional file 1.

In a recently published review article, several considerations for selecting effective siRNAs were proposed resulting from summarization and integration of major recent findings in the field of siRNA design [41]. Comparison of these considerations with the survey results obtained in this study indicates that they generally agree with each other (see Supplementary Table 8 in Additional file 1). Of the 34 features pertinent to the considerations proposed by Pei and Tuschl, 29 were found to be significant in boosting the siRNA efficacy. Among the remaining 5 features, the feature *G/C content is between 30 and 52% *was found to be associated with a commensurate, though not significant improvement in the efficacy distribution (*P*_*70 *_= 0.082 and *P*_*wald *_= 0.056). Two related features, *G/C content is between 35 and 60% *and *G/C content is between 31.6 and 57.9%*, however, were found to be highly significant in boosting the siRNA efficacy, agreeing with the common understanding that the effective siRNAs prefer a low-to-medium G/C content. Two features pertinent to the considerations proposed by Pei and Tuschl that are related to target accessibility, *siRNA passes the repelling loop filter *and *Anti-sense siRNA binding energy > -10 KCal/Mol *were not found to be significant in our survey. Yet, two other features closely related to them, *H-b index < 28.8 *and *Local free energy of the most stable structure ≥ -20.9 KCal/Mol*, were found to be significant. The remaining two features pertinent to the considerations proposed by Pei and Tuschl, *Binding energy of N6–N11 ≥ -13 KCal/Mol *and *10th nucleotide = (A/U)*, were not found to be significant in our survey.

Since the *siRecords *collection is compiled from published siRNA studies, there is the concern that it may be biased towards higher efficacy siRNAs, because researchers are probably less inclined to report lower efficacy experiments in their research articles. We can assess how much this bias is by comparing the efficacy distribution of the *siRecords *collection with that of published randomly designed siRNAs. In two published studies [11,22], moderately large numbers (180) of randomly designed siRNAs were tested for knock-down efficacies. The percentages of siRNAs resulting in < 50% efficacies in these two studies were 22.2% and 23.3%, respectively. In the *siRecords *data used in this study, the percentage of records receiving "low" efficacy rating (i.e., produced < 50% knock-down efficacies) is 14.3%. In one of these previous studies [22], the percentage of siRNAs resulting in > 90% efficacies was reported to be 29.4%. In the *siRecords *collection, the percentage of records receiving "very high" efficacy rating (i.e., produced > 90% efficacies) is 34.3%. Therefore, the *siRecords *collection is indeed biased towards the higher efficacy experiments, likely because researchers are less ready to report lower efficacy experiments. However, this bias is not severe, because nearly 2/3 of the low efficacy siRNA experiments are still included in *siRecords*. Furthermore, the analyses conducted in this study – in particular, the results of the survey of features influencing the siRNA efficacy – are not influenced by the reduced number of low efficacy siRNAs in the dataset. These analyses are reliable as long as the dataset includes sufficiently large number of low efficacy records (the number of records bearing "low" efficacy used in this study is 467).

Another concern over the using of the siRNA data compiled from published siRNA studies is that the design of siRNA experiments in these published studies might be dominated by one or two design tools used in the performance comparison (Table 3), compromising the objectiveness of this comparison. An analysis of the relative utility of the 15 online siRNA design tools (see Supplementary Table 7 in Additional file 1) suggested that these design tools had varied levels of utility, yet none of them had dominated the current siRNA design practice (see discussion in Additional file 1).

It is desirable to validate the DRM rule sets obtained in this study using a dataset independent of *siRecords*. However, it is considerably difficult to find a separate siRNA efficacy dataset that is as large and diverse as the *siRecords *collection. In a recent report by Huesken et al., a genome-wide human siRNA library was constructed, in which 2,431 randomly selected siRNAs targeting 34 fusion mRNAs were tested for efficacy [42]. There were concerns when this library of siRNAs was considered as a validation dataset for the DRM rule sets, because, firstly, this dataset is of a singular origin; and secondly, fusion mRNAs were used against which the siRNA efficacies were tested. This is considered as a somewhat questionable practice because the native secondary structures may not be well preserved in the fusion mRNAs. Although Huesken et al. performed control experiments which suggested that fusion mRNAs and endogenous mRNAs produced similar efficacy estimates in the setting they adopted, and argued that sequence features, rather than secondary structure related features were the main determinants of the siRNA efficacy, there have been multiple recent reports about secondary structures playing important roles in determining the siRNA efficacy [17,25], which are backed up by the finding in our survey that at least one secondary structure related feature (*Local folding potential (mean) *≥ *-22.72 KCal/Mo*l) significantly boosts the chance of achieving higher siRNA efficacy. Nevertheless, we examined the performance of the DRM rule sets using the 2,431 siRNA dataset provided by Huesken et al. The three DRM rule sets with the highest stringency (*RS*_*0.951*_, *RS*_*0.895 *_and *RS*_0.845_) identified 23, 32 and 48 effective siRNAs, respectively, in this dataset. These selected siRNAs had average "normalized inhibitory activity" of 0.80, 0.78 and 0.76, respectively. When tested using the 249-siRNA test dataset specified in that study, the same three DRM rule sets identified 3, 4 and 6 effective siRNAs, respectively, and the average "normalized inhibitory activity" of these siRNAs were 0.96, 0.80 and 0.78, respectively. In Huesken et al., the average "normalized inhibitory activity" of the entire dataset was 0.69, and they recommended to use 0.75 or 0.80 as cut-offs for selecting effective siRNAs. These results suggest that generally speaking, the DRM rule sets were capable of identifying effective siRNAs in this completely independent siRNA efficacy dataset.

As more data becomes available in *siRecords*, we will perform updated analyses on this data collection with the aim of obtaining more accurate and more reliable siRNA design rules. In addition, as there is indication that the DRM rule sets behave differently for subpopulations of siRNAs tested under different experimental settings (e.g., for those validated with Western blot technique and those validated with PCR and other techniques, see Supplementary Figure 4 in Additional file 1), we will refine our analyses and develop separate rule sets for these different subpopulations of siRNAs.

## Conclusion

In this study, we identified a bundle of highly effective and generally applicable rule sets for siRNA design. This was accomplished by applying a simple strategy in which we analyzed a large number of candidate features for association with increased siRNA efficacies, then used quantitative analyses of the joint effects of these significant features to identify positive cooperativity among these features. The key to our approach was the use of the large set of siRNA efficacy data available in *siRecords*. The availability of this dataset not only made the execution of this strategy possible, but also curbed the overfitting problem that many rules generated by other design protocols suffer from. We expect that the design rules revealed in this study, together with improving RNAi lab techniques, will make siRNAs a more useful tool for molecular genetics, functional genomics, and drug discovery studies.

## Methods

### Data preparation

All records of 19-mer siRNAs (not counting the overhanging nucleotides on the 3' end) were retrieved from the *siRecords *database. The records that failed to meet the following criteria were excluded from further analyses: (1) had complete annotations of cell line types, test methods, transfection methods and efficacy classification; (2) had target mRNA lengths ≤ 16,000 nucleotides (this is a limit set by the Mfold program for calculation of thermodynamics features, see below); (3) the siRNA sequence had no mismatches with the targeted site by pair-wise Blast (NCBI bl2seq v.2.2.9, parameters "-p blastn -W 7 -q -1 -F F"). For studies where more than 30 siRNA experiments were reported, we randomly chose 30 to include in our analyses. The cell line types and test methods were grouped based on ATCC (American Type Culture Collection) [43] and Protocol Online [44], respectively.

### Features

We define a *feature *as a binary property of a siRNA experiment concerning a factor potentially influencing the efficacy of the experiment. For a given siRNA experiment, any defined feature is either present or absent. Some example features are listed below:

*(1) The 6th nucleotide of the siRNA sequence *(counting from the 5' end on the sense strand) *is an adenine (A)*.

*(2) The 17th nucleotide of the siRNA sequence is not a guanine (G)*.

*(3) There are at least three (A/U)'s in the seven nucleotides on the 3' end*.

*(4) The G/C content of the siRNA sequence is between 30 and 52%*.

For Features (1) and (2), the concerning factors potentially influencing the siRNA efficacy are the identities of the 6th and the 17th nucleotides of the siRNA sequence, respectively. For Feature (3), the concerning factor is the seven nucleotides as a whole on the 3' end of siRNA sequence. For Feature (4), the concerning factor is the G/C content of the siRNA sequence.

Each feature has a *complementary feature*, that is, the alternative property concerning the same factor. For instance, the complementary feature of Feature (1) is *the 6th nucleotide of the siRNA sequence *≠ *A*; and the complementary feature of Feature (3) is *there are at most 2 (A/U)'s in the seven nucleotides on the 3' end*. For any given siRNA experiment and any given feature, either the feature holds true for the experiment, or the complementary feature must hold true. A feature and its complementary feature constitute a *feature pair*.

For a given factor, there are multiple ways of formulating features. In some cases, the so-called *sub-feature *– *super-feature *relationships can result. For example, the following four features are all concerned with same factor – the identity of the 6th nucleotide of the siRNA sequence:

*(5) The 6th nucleotide *= *A*.

*(6) The 6th nucleotide *≠ *A*.

*(7) The 6th nucleotide *=*C*.

*(8) The 6th nucleotide *≠ *C*.

Wherever Feature (5) is present, Feature (8) must also be present. Thus, Feature (5) is a sub-feature of Feature (8), and Feature (8) is a super-feature of Feature (5). Similarly, Features (7) and (6) also constitute a pair of sub-feature – super-feature relationship.

### Feature definitions

We surveyed 276 features (constituting 138 feature pairs) in this study. These features can be classified into the following five categories:

#### Category 1: Direct sequence features

We defined 152 direct sequence features (76 pairs) based on the positional specific nucleotide identity in the siRNA sequence (on the sense strand). For each position in the 19-mer siRNA sequence, 8 features were defined based on whether or not the nucleotide at the position is an adenine (A), a cytosine (C), a guanine (G), or a uracil (U), respectively. Among these features, 24 were previously claimed to favorably influence the siRNA efficacy (see Supplementary Table 1 in Additional file 1).

#### Category 2: Sequence-derived features

We defined 38 sequence-derived features (19 pairs) that are related to either the sequence compositions or the G/C content of the siRNA (see Supplementary Table 2 in Additional File 1). All these features have been previously claimed to have impact on the siRNA efficacy. Among them, 24 features were defined based on (*a*) whether or not the 1st nucleotide is a G/C [13,31,45], (*b*) whether or not the 10th nucleotide is an A/U [45], (*c*) whether the 11th nucleotide is a G/C [14], (*d*) whether the 19th nucleotide is an A/U [13,31,45], (*e*) whether there are at least 5 (A/U)'s in the last 7 nucleotides at the 3' end [13], (*f*) whether there are at least 3 (A/U)'s in the last 7 nucleotides in the 3' end [45], (*g*) whether there are at least 3 (A/U)'s in the 5 nucleotides at the 3' end [11], (*h*) whether the siRNA contains G/C stretches longer than 9 [13,46], (*i*) whether the siRNA contains G/C stretches longer than 7 [18,19], (*j*) whether there are occurrences of 3 or more identical nucleotides in a row [18,32], (*k*) whether there are occurrences of 4 or more identical nucleotides in a row [16,18,19,47], and (*l*) whether there are at least 3 (A/U)'s in the 5 nucleotides at the 5' end [11], respectively. In addition, 14 features (7 pairs) were defined based on whether the G/C content of the siRNA falls into the following reported optimal G/C ranges: (*a*) 30 – 52% [11], (*b*) 32 – 79% [12], (*c*) 30 – 70% [30], (*d*) 35 – 60% [18], (*e*) 20 – 50% [32], (*f*) 31.6 – 57.9% [31] and (*g*) 30 – 79% [16].

#### Category 3: Features defined based on thermodynamics of the siRNA

*Features on T_*m*_, folding energy of the sense strand and total hairpin energy*. Ten features (5 pairs) were defined that are related to the melting temperature (T_m_) of the siRNA, the folding energy of sense strand, or the total hairpin energy of the siRNA. Among them, 6 features were defined based on whether or not the T_m _falls into the following three ranges < 60°C, < 20°C, and between 20 and 60°C [11]. Two features were defined based on whether or not the folding energy of sense strand is equal to or greater than -5 KCal/Mol [18]. Two features were defined based on whether the absolute value of total hairpin energy is less than 1 KCal/Mol [24]. The DINAMelt server [48] was used in the calculation of T_m _and hairpin energy [29,49]. The total hairpin energy was calculated as the absolute value of the sum of hairpin energies of siRNA sense and anti-sense strand in units of KCal/Mol [24] (Chalk, A., personal communication).

*Features on binding energy*. Sixteen features (8 pairs) related to the binding energy of siRNA sequences were defined. On the 5' end binding energy, we defined the feature *5' binding energy is between -9 and -5 KCal/Mol *and its complementary feature [24]. On mid-sequence binding energy, we defined 6 features associated with three nucleotide ranges: N6–N11 [22], N7–N11 [15] and N7–N12 [24]. For the nucleotide range N7–N12, we used the reported threshold -13KCal/Mol in the feature definition [24]. For the nucleotide range N7–N11, we defined the feature based on whether or not the average free energy profiles fall into the reported optimized range between -1.97 and -1.65 KCal/Mol [15]. For the binding energy of the range N6–N11 for which no threshold was explicitly reported, we took the median value (-13 KCal/Mol) of all siRNAs in the dataset as the threshold. On 3' end binding energy, we defined a feature *binding energy of N16–N19 > -9 KCal/Mol *and its complementary feature [24]. In addition, 6 features (3 pairs) were defined that are associated with the difference between the 5' binding energy and 3' binding energy. They are defined based on: (*a*) whether or not the difference between the binding energy of N1–N4 and N16–N19 is greater than 0 [22,24], (*b*) whether or not the difference between the binding energy of N1–N4 and N16–N19 is between 0 and 1 KCal/Mol [24], and (*c*) whether or not the difference between the binding energy of N1–N5 and N15–N19 is greater than 0 [15], respectively (see Supplementary Table 3 in Additional file 1).

The nearest neighbor model parameters described in Xia, T. et al. [50] were used for binding energy calculation [29]. The binding energy of N1–N4 and N16–N19 were computed as the sum of free energies for 4 base-pair stacks starting from position 1 in the sense strand and one single base stacking energy [21,51] (Chalk, A., personal communication). Calculations of binding energies for N1–N5 and N15–N19 were performed similarly to those done for N1–N4 and N16–N19, except that 5 base-pair stacks were used. Binding energies for N6–N11 and N7–N12 were computed as the sum of free energies for 6 base-pair stacks within positions 6–11 and positions 7–12 in the sense strand. The average free energy profiles of N7–N11 was computed as the average base pair energy of consecutive five pentamer subsequences starting from positions 7 to 11 in the sense strand (Poliseno, L., personal communication).

#### Category 4: Features defined based on target mRNA sites

*Features on the mRNA target location*. Sixteen features (8 pairs) related to the siRNA target location on mRNA were defined, based on whether or not the target region is within (*a*) 5' UTR [12,16], (*b*) 3' UTR [16], (*c*) CDS[18], (*d*) the first 100 nucleotides of CDS [12,16], (*e*) the first quartile of CDS, (*f*) the second quartile of CDS, (*g*) the third quartile of CDS[14], and (*h*) the fourth quartile of CDS, respectively. The mRNA sequences were obtained from NCBI GenBank. The target region was determined by using a BLAST search (NCBI bl2seq v.2.2.9 with parameter "-W 7 -q -1 -F F"). The targeted site was assigned to a sub-region if the entire target site lied within that sub-region.

*Feature on the secondary structures of the target mRNA*. Fourteen features (7 pairs) that are associated with the secondary structures of the target mRNA were defined, based on (*a*) whether or not the calculated hydrogene bond (H-b) index is less than 28.8 [25], (*b*) whether or not the siRNA target region is filtered by repelling loop filter [52], (*c*) whether or not *the local free energy of the most stable structure (LFE_mss) *is equal to or greater than -20.9 KCal/Mol [53], (*d*) whether *the average local free energy of the ten most stable structures (LFE_average) *is equal to or greater than -20.85 KCal/Mol [53], (*e*) whether or not the mean local folding potential (LFP) is equal to or greater than -22.72 KCal/Mol, (*f*) whether or not a non-zero *accessibility score *was obtained for the siRNA target site [54], (*g*) whether or not the anti-sense siRNA binding energy is equal to or less than -10KCal/Mol [47], respectively (see Supplementary Table 4 in Additional file 1).

The hydrogen bond (H-b) index measures the average number of hydrogen bonds formed between nucleotides in the target region and the rest of mRNA, and it was calculated according to Luo et al. [25]. We used the median value of all siRNAs in the dataset (28.8) as the threshold since no threshold was explicitly given in the original report. The repelling loop filter was proposed by Yiu et al. for determining the accessibility of the mRNA target region [52]. If in at least three of the five most stable structures of the whole-length mRNA (calculated with Mfold), the 19-mer target site was contained by at least one "big repelling loop", or by at least two "repelling loops", the target region was identified to be invalid by the repelling loop filter. The LFE (local free energy) was calculated according to Schubert, S., et.al[53], with predicted mRNA secondary structures calculated using Mfold 3.2 [29,55]. The free energy contribution of each *sequence local element *in a structure was extracted from the output .*det *files by Mfold; local elements include helices, bulges, and loops among others. The LFE of the targeted site was computed as the sum of the free energy contribution of all *sequence local elements *containing one or more nucleotides in the siRNA target site (Schubert, S., personal communication). The ten most stable secondary structures in the mRNA sequence were also used in our calculations. For each siRNA target site, we calculated the LFE for the lowest free energy structure of the site (*LFE_mss*) and the average LFE of the ten most stable secondary structures (*LFE_average*). Since no thresholds were explicated provided in the original report, the medians of all LFE values in the dataset (-20.9 KCal/Mol for *LFE_mss *and -20.85 for the *LFE_average*) were used as thresholds in the feature definitions.

The local folding potential (LFP) is a measurement of the RNA local thermodynamic stability [56-58]. We postulated that the thermodynamic stability of the siRNA target site may influence the RNAi effectiveness. We calculated the structure with the lowest free energy for the 100 nucleotide region on the mRNA centering around each of the 19 nucleotides in the siRNA target site. The LFP was calculated as the mean of the 19 free energy values obtained. In cases when the target site was close to either end of the mRNA, so that the 100-nucleotide regions could not be obtained for certain nucleotides in the 19-mer target site, a shorter mRNA segment was used that was truncated at the end of mRNA. The median value calculated for the entire dataset (-22.72 KCal/Mol) was used as the threshold in feature definition.

The accessibility of the siRNA target region was recently raised as an important factor influencing the siRNA efficacy [59]. We conducted the *Iterative computational analysis *(ICA) using a window size = 800 nucleotides and a step size = 100 nucleotides [59,60]. To generate the largest number of windows that overlap the siRNA target region, the central base of the siRNA target region was used as the central point of the first window; subsequent windows were extended in both directions to cover the entire mRNA sequence. For each window, the five most stable structures predicted by Mfold were used. It turned out, however, that the ICA routine produced a filter that is too stringent for practical use. Of the 2,600 siRNA target regions in our dataset, only 6 were determined to be assessable by this routine. We then took an alternative approach, and conducted the *accessibility score *analysis [54], which produced a similar but less stringent filter. In calculating the *accessibility score*, a region receives a non-zero score as long as the most stable structure in each window covering the siRNA target region contains a single-strand segment of length ≥ 10 nucleotides. Of the 2,600 siRNA target regions in our dataset, 456 received non-zero accessibility scores. Two features were defined based on this accessibility score filter.

The anti-sense siRNA binding energy was proposed as a measurement of mRNA accessibility [47]. We used the Sirna module of the Sfold server to calculate the *anti-sense siRNA binding energy *[47]. For each siRNA target sequence, a 200 nucleotide mRNA segment centering around the 19 nucleotide target site was extracted. In cases when the target site was close to either end of the mRNA sequence, so that a 200-nucleotide regions centering around the target site could not be obtained, a shorter mRNA segment (truncated at the close end of the mRNA) was used. These segments were sent to the Sirna server for calculation [61]. The results were parsed and the anti-sense siRNA binding energies were extracted.

#### Category 5: Features defined based on experimental conditions

The experimental conditions considered in our analysis include cell line types, test methods, transfection methods and test objects. Twelve features (6 pairs) were defined based on whether or not the RNAi experiment is conducted any of the 6 most frequently used cell lines: (*a*) HeLa, (*b*) HEK293, (*c*) MCF7, (*d*) CV-1 and derivatives, (*e*) 3T3 and (*f*) T24. Twelve features (6 feature pairs) were defined based on whether or not the test method is one of the six most frequently used test methods: (*a*) Western blot, (*b*) PCR (including RT-PCR, real-time PCR etc.), (*c*) bDNA, (*d*) Northern blot, (*e*) Luciferase assay, and (*f*) Flow cytometry. Two features were defined based on whether the transfection method is synthetic siRNAs or transcription of hairpin precursors. Four features (2 feature pairs) were defined based on whether or not the tested object is (*a*) mRNA or (*b*) protein (see Supplementary Table 5 in Additional file 1).

### Statistical tests of features influencing siRNA efficacy

Determined by the four-level scheme used to rate the efficacy of siRNA experiments, proper categorical analysis techniques were needed to analyze these data. For any given feature, we calculated the efficacy distribution (among the four levels – very high, high, medium and low) of all siRNA experiments carrying this feature, and compared it with the efficacy distribution of all siRNA experiments carrying the complementary feature of this feature. Chi-square (*χ*^2^) test of independence is a commonly used test that finds evidence of difference between two discrete distributions. However, this test assumes that the dependent variable (efficacy rating) is a nominal variable rather than an ordinal variable, thus it is not able to tell us whether the presence of a feature results in higher or lower efficacy. A more appropriate test will find evidence of monotone trend, that is, whether the presence of a feature is associated with a significant up-shift or down-shift of the efficacy distributions among the four levels. Consider the joint probability distribution {*π*_*i*, *j*_} between the presence/absence of a particular feature (which defines *i*: *i *= 1 if the feature is present, and *i *= 0 if the feature is absent), and the four-level efficacy ratings (which defines *j*: *j *= 3 if efficacy rating = "very high", *j *= 2 if efficacy rating = "high", *j *= 1 if efficacy rating = "medium", and *j *= 0 if efficacy rating = "low"). We calculate the probabilities of concordance and discordance:

Πc=2∑i∑jπij(∑h>i∑k>jπhk),Πd=2∑i∑jπij(∑h>i∑k<jπhk).
 MathType@MTEF@5@5@+=feaafiart1ev1aaatCvAUfKttLearuWrP9MDH5MBPbIqV92AaeXatLxBI9gBaebbnrfifHhDYfgasaacH8akY=wiFfYdH8Gipec8Eeeu0xXdbba9frFj0=OqFfea0dXdd9vqai=hGuQ8kuc9pgc9s8qqaq=dirpe0xb9q8qiLsFr0=vr0=vr0dc8meaabaqaciaacaGaaeqabaqabeGadaaakqaabeqaaiabfc6aqnaaBaaaleaacqWGJbWyaeqaaOGaeyypa0JaeGOmaiZaaabuaeaadaaeqbqaaGGaciab=b8aWnaaBaaaleaacqWGPbqAcqWGQbGAaeqaaaqaaiabdQgaQbqab0GaeyyeIuoaaSqaaiabdMgaPbqab0GaeyyeIuoakiabcIcaOmaaqafabaWaaabuaeaacqWFapaCdaWgaaWcbaGaemiAaGMaem4AaSgabeaaaeaacqWGRbWAcqGH+aGpcqWGQbGAaeqaniabggHiLdGccqGGPaqkcqGGSaalaSqaaiabdIgaOjabg6da+iabdMgaPbqab0GaeyyeIuoaaOqaaiabfc6aqnaaBaaaleaacqWGKbazaeqaaOGaeyypa0JaeGOmaiZaaabuaeaadaaeqbqaaiab=b8aWnaaBaaaleaacqWGPbqAcqWGQbGAaeqaaaqaaiabdQgaQbqab0GaeyyeIuoaaSqaaiabdMgaPbqab0GaeyyeIuoakiabcIcaOmaaqafabaWaaabuaeaacqWFapaCdaWgaaWcbaGaemiAaGMaem4AaSgabeaaaeaacqWGRbWAcqGH8aapcqWGQbGAaeqaniabggHiLdGccqGGPaqkcqGGUaGlaSqaaiabdIgaOjabg6da+iabdMgaPbqab0GaeyyeIuoaaaaa@73D9@

Then we calculate the *γ *difference between these two probabilities:

γ=Πc−ΠdΠc+Πd.
 MathType@MTEF@5@5@+=feaafiart1ev1aaatCvAUfKttLearuWrP9MDH5MBPbIqV92AaeXatLxBI9gBaebbnrfifHhDYfgasaacH8akY=wiFfYdH8Gipec8Eeeu0xXdbba9frFj0=OqFfea0dXdd9vqai=hGuQ8kuc9pgc9s8qqaq=dirpe0xb9q8qiLsFr0=vr0=vr0dc8meaabaqaciaacaGaaeqabaqabeGadaaakeaaiiGacqWFZoWzcqGH9aqpdaWcaaqaaiabfc6aqnaaBaaaleaacqWGJbWyaeqaaOGaeyOeI0IaeuiOda1aaSbaaSqaaiabdsgaKbqabaaakeaacqqHGoaudaWgaaWcbaGaem4yamgabeaakiabgUcaRiabfc6aqnaaBaaaleaacqWGKbazaeqaaaaakiabc6caUaaa@3E33@

The sample *γ *has approximately a normal distribution, with standard error calculated using the Delta method

σ2=16(Πc+Πd)4∑i∑jπij[Πdπij(c)−Πcπij(d)]2,
 MathType@MTEF@5@5@+=feaafiart1ev1aaatCvAUfKttLearuWrP9MDH5MBPbIqV92AaeXatLxBI9gBaebbnrfifHhDYfgasaacH8akY=wiFfYdH8Gipec8Eeeu0xXdbba9frFj0=OqFfea0dXdd9vqai=hGuQ8kuc9pgc9s8qqaq=dirpe0xb9q8qiLsFr0=vr0=vr0dc8meaabaqaciaacaGaaeqabaqabeGadaaakeaaiiGacqWFdpWCdaahaaWcbeqaaiabikdaYaaakiabg2da9maalaaabaGaeGymaeJaeGOnaydabaGaeiikaGIaeuiOda1aaSbaaSqaaiabdogaJbqabaGccqGHRaWkcqqHGoaudaWgaaWcbaGaemizaqgabeaakiabcMcaPmaaCaaaleqabaGaeGinaqdaaaaakmaaqafabaWaaabuaeaacqWFapaCdaWgaaWcbaGaemyAaKMaemOAaOgabeaaaeaacqWGQbGAaeqaniabggHiLdaaleaacqWGPbqAaeqaniabggHiLdGccqGGBbWwcqqHGoaudaWgaaWcbaGaemizaqgabeaakiab=b8aWnaaDaaaleaacqWGPbqAcqWGQbGAaeaacqGGOaakcqWGJbWycqGGPaqkaaGccqGHsislcqqHGoaudaWgaaWcbaGaem4yamgabeaakiab=b8aWnaaDaaaleaacqWGPbqAcqWGQbGAaeaacqGGOaakcqWGKbazcqGGPaqkaaGccqGGDbqxdaahaaWcbeqaaiabikdaYaaakiabcYcaSaaa@62D1@

where

πij(c)=∑i>h∑j>kπhk+∑h>i∑k>jπhk,πij(d)=∑i>h∑j<kπhk+∑h>i∑k<jπhk.
 MathType@MTEF@5@5@+=feaafiart1ev1aaatCvAUfKttLearuWrP9MDH5MBPbIqV92AaeXatLxBI9gBaebbnrfifHhDYfgasaacH8akY=wiFfYdH8Gipec8Eeeu0xXdbba9frFj0=OqFfea0dXdd9vqai=hGuQ8kuc9pgc9s8qqaq=dirpe0xb9q8qiLsFr0=vr0=vr0dc8meaabaqaciaacaGaaeqabaqabeGadaaakqaabeqaaGGaciab=b8aWnaaDaaaleaacqWGPbqAcqWGQbGAaeaacqGGOaakcqWGJbWycqGGPaqkaaGccqGH9aqpdaaeqbqaamaaqafabaGae8hWda3aaSbaaSqaaiabdIgaOjabdUgaRbqabaaabaGaemOAaOMaeyOpa4Jaem4AaSgabeqdcqGHris5aaWcbaGaemyAaKMaeyOpa4JaemiAaGgabeqdcqGHris5aOGaey4kaSYaaabuaeaadaaeqbqaaiab=b8aWnaaBaaaleaacqWGObaAcqWGRbWAaeqaaaqaaiabdUgaRjabg6da+iabdQgaQbqab0GaeyyeIuoaaSqaaiabdIgaOjabg6da+iabdMgaPbqab0GaeyyeIuoakiabcYcaSaqaaiab=b8aWnaaDaaaleaacqWGPbqAcqWGQbGAaeaacqGGOaakcqWGKbazcqGGPaqkaaGccqGH9aqpdaaeqbqaamaaqafabaGae8hWda3aaSbaaSqaaiabdIgaOjabdUgaRbqabaaabaGaemOAaOMaeyipaWJaem4AaSgabeqdcqGHris5aaWcbaGaemyAaKMaeyOpa4JaemiAaGgabeqdcqGHris5aOGaey4kaSYaaabuaeaadaaeqbqaaiab=b8aWnaaBaaaleaacqWGObaAcqWGRbWAaeqaaaqaaiabdUgaRjabgYda8iabdQgaQbqab0GaeyyeIuoaaSqaaiabdIgaOjabg6da+iabdMgaPbqab0GaeyyeIuoakiabc6caUaaaaa@8321@

Let

z2=γ2σ2,
 MathType@MTEF@5@5@+=feaafiart1ev1aaatCvAUfKttLearuWrP9MDH5MBPbIqV92AaeXatLxBI9gBaebbnrfifHhDYfgasaacH8akY=wiFfYdH8Gipec8Eeeu0xXdbba9frFj0=OqFfea0dXdd9vqai=hGuQ8kuc9pgc9s8qqaq=dirpe0xb9q8qiLsFr0=vr0=vr0dc8meaabaqaciaacaGaaeqabaqabeGadaaakeaacqWG6bGEdaahaaWcbeqaaiabikdaYaaakiabg2da9maalaaabaacciGae83SdC2aaWbaaSqabeaacqaIYaGmaaaakeaacqWFdpWCdaahaaWcbeqaaiabikdaYaaaaaGccqGGSaalaaa@3706@

then *z*^2 ^is a Wald statistics that has a chi-squared null distribution with 1 degree of freedom, based on which a Wald test can be conducted to find significant monotone trend [35].

The monotone trend test finds evidence about whether the presence of a particular feature is associated with significant up-shift of the four-level efficacy distribution. If the evidence of such association is found, however, this test alone is not able to tell us where the up-shift takes place. In RNAi experiments, we are most concerned with the chances of achieving higher efficacies. Thus, we also conducted permutation tests of odds ratios for achieving > 90% and > 70% efficacies. In the *siRecords *data, the chance of achieving > 90% (or > 70%) efficacies can be approximated by the proportion of records bearing "very high" (or "high"/"very high") efficacy ratings. For a given feature, the odds ratio for > 90% efficacies, *θ*_90_, is defined as

θ90=π1,>90/(1−π1,>90)π0,>90/(1−π0,>90),
 MathType@MTEF@5@5@+=feaafiart1ev1aaatCvAUfKttLearuWrP9MDH5MBPbIqV92AaeXatLxBI9gBaebbnrfifHhDYfgasaacH8akY=wiFfYdH8Gipec8Eeeu0xXdbba9frFj0=OqFfea0dXdd9vqai=hGuQ8kuc9pgc9s8qqaq=dirpe0xb9q8qiLsFr0=vr0=vr0dc8meaabaqaciaacaGaaeqabaqabeGadaaakeaaiiGacqWF4oqCdaWgaaWcbaGaeGyoaKJaeGimaadabeaakiabg2da9maalaaabaGae8hWda3aaSbaaSqaaiabigdaXiabcYcaSiabg6da+iabiMda5iabicdaWaqabaGccqGGVaWlcqGGOaakcqaIXaqmcqGHsislcqWFapaCdaWgaaWcbaGaeGymaeJaeiilaWIaeyOpa4JaeGyoaKJaeGimaadabeaakiabcMcaPaqaaiab=b8aWnaaBaaaleaacqaIWaamcqGGSaalcqGH+aGpcqaI5aqocqaIWaamaeqaaOGaei4la8IaeiikaGIaeGymaeJaeyOeI0Iae8hWda3aaSbaaSqaaiabicdaWiabcYcaSiabg6da+iabiMda5iabicdaWaqabaGccqGGPaqkaaGaeiilaWcaaa@5639@

where *π*_1, > 90 _is the proportion of records bearing "very high" efficacy rating (i.e., with > 90% efficacies) in the subset of the experiments carrying the feature, and *π*_0, > 90_is the proportion of records bearing "very high" efficacy ratings in the subset of the experiments carrying the complementary feature of the feature concerned. To generate a null distribution of the odds ratio, Set A was randomly split into two subsets, one of which was arbitrarily marked with "feature present", the other marked with "complementary feature present", and an odds ratio was calculated accordingly. This process was repeated 100000 times, and the 100000 resampled odds ratios constituted the null distribution. Given any feature to be tested, the P value was calculated as

*P*_90 _= (*i*|θ90i
 MathType@MTEF@5@5@+=feaafiart1ev1aaatCvAUfKttLearuWrP9MDH5MBPbIqV92AaeXatLxBI9gBaebbnrfifHhDYfgasaacH8akY=wiFfYdH8Gipec8Eeeu0xXdbba9frFj0=OqFfea0dXdd9vqai=hGuQ8kuc9pgc9s8qqaq=dirpe0xb9q8qiLsFr0=vr0=vr0dc8meaabaqaciaacaGaaeqabaqabeGadaaakeaacqaH4oqCdaqhaaWcbaGaeGyoaKJaeGimaadabaGaemyAaKgaaaaa@31D8@ > *θ*_90_)/100000,

where θ90i
 MathType@MTEF@5@5@+=feaafiart1ev1aaatCvAUfKttLearuWrP9MDH5MBPbIqV92AaeXatLxBI9gBaebbnrfifHhDYfgasaacH8akY=wiFfYdH8Gipec8Eeeu0xXdbba9frFj0=OqFfea0dXdd9vqai=hGuQ8kuc9pgc9s8qqaq=dirpe0xb9q8qiLsFr0=vr0=vr0dc8meaabaqaciaacaGaaeqabaqabeGadaaakeaacqaH4oqCdaqhaaWcbaGaeGyoaKJaeGimaadabaGaemyAaKgaaaaa@31D8@ is the *i*th resampled odds ratio, and *θ*_90 _is the true odds ratio of the feature. The odds ratio permutation test for > 70% efficacies was conducted similarly, with the proportion of records bearing "very high" or "high" efficacy ratings substituted for that of records bearing "very high" ratings in the above description.

Meaningful statistics tests require the use of sufficiently large datasets. All features were subject to a "dataset size filter" using an arbitrarily set threshold of 30 records: if a given feature was carried by fewer than 30 records in Set A, then this feature and the complementary feature of this feature were excluded from the statistics tests and following analyses. Four features – *GC stretches of length *≥ *9*, *G/C content is not between 30 and 79%, Cell line = T24 *and *Test method = Flow cytometry*, as well as their complementary features were excluded for this reason.

### Control of false discovery rate (FDR)

The simultaneous testing of the large number of hypotheses requires the curbing of the type I error rate with the consideration of the "multiple testing" problem. We chose to control the FDR by taking the q-value approach [36], because of its ability to adapt to the true distribution of the input p-values. We used the "bootstrap" method, rather than the default "smoother" method (which is equivalent to Benjamini and Hochberg's FDR controlling method [62]) in estimating the FDR, because U-shape distributions were observed for the input p-values for both the Wald test and the odds ratio permutation tests, likely introduced by the fact that one-sided tests were conducted when two-sided signals were present [63].

### Rules, rule sets and the disjunctive rule merging (DRM) algorithm

We define a *rule *as a conjunction of (*l*) features. An *l*-feature rule is also called an *l*-feature combination. A *rule set *is defined as a disjunction of (*m*) rules. Generally speaking, the larger *m *is, the higher sensitivity the rule set achieves, in the mean time, the lower specificity the rule set has to offer.

The *disjunctive rule merging (DRM) algorithm *was developed to remove the redundancy in the rule sets resulting from the combined effect analysis of multiple features, in the mean time exerting control over the stringency of the rule sets. The listing of the DRM algorithm is as follows.

**Input**: Θ: a set of disjunctive rules that contains redundancy; each rule, *r*_*i*_, is a conjunction of *m*_*i *_features: *r_i _*= {*f_i_*, *f*_2_,..., fmi
 MathType@MTEF@5@5@+=feaafiart1ev1aaatCvAUfKttLearuWrP9MDH5MBPbIqV92AaeXatLxBI9gBaebbnrfifHhDYfgasaacH8akY=wiFfYdH8Gipec8Eeeu0xXdbba9frFj0=OqFfea0dXdd9vqai=hGuQ8kuc9pgc9s8qqaq=dirpe0xb9q8qiLsFr0=vr0=vr0dc8meaabaqaciaacaGaaeqabaqabeGadaaakeaacqWGMbGzdaWgaaWcbaGaemyBa02aaSbaaWqaaiabdMgaPbqabaaaleqaaaaa@3123@}, and is labeled with *P_i _*= the proportion of records reaching > 90% efficacy in the subset of Set A records satisfying *r_i_*.

*α*: stringency factor, with a range of [0, 1].

**Initialization: **Create rule set *RS *= *φ*.

**Step 1:**For every *r*_*i *_∈ Θ satisfying *P*_*i *_≥ *α*, add *r*_*i *_into *RS*.

**Step 2: **For *j *= 2,3,...,5

      For any rule *r*_*p *_∈ *RS *where *m*_*p *_= *j*

         For any rule *r*_*q *_∈ *RS *where *m*_*q *_> *j*

            if *r*_*p *_⊂ *r*_*q*_, then remove *r*_*q *_from *RS*.

         End For

      End For

   End For

**Output: ***RS*: non-redundant set of disjunctive rules with stringency >*α*.

It is easy to see that given any *α*, the rule set resulting from the DRM algorithm (thus called a DRM rule set) is fixed. The reverse, however, is not true. A DRM rule set does not correspond to a single *α *value, but rather, a range of different *α*'s. For example, the DRM rule sets for any *α *between 0.901 and 1 are exactly the same (containing 7 rules). We note this rule set as *RS*_*0.951*_, where 0.951 is the mid-point of the range of *α *for which the rule sets are produced.

Naturally, the higher *α *level, the higher specificity the DRM rule set possesses; meanwhile, the lower sensitivity the rule set has to offer. Therefore, the DRM algorithm with variable *α *values allows us to choose the proper combination of sensitivity and specificity that suits our needs. In the siRNA design of a typical setting, we are most concerned with achieving high specificity, and can often tolerate lower sensitivity, since there is a large pool of possible target sites to choose from – for a mRNA of length *w*, in theory there are (*w*-19+1) target sites to pick from. Therefore, we are most concerned with the behavior of the rule sets with high (close to 1) *α *values.

### Performance comparison between DRM rule sets and existing online design tools

Design tasks were performed for the 744 genes in Set T using the following 15 online siRNA design tools with the default settings.

*Ambion siRNA Target Finder (Ambion, Inc.) *[64]. We used the mRNA sequence as the input. By default, no restriction of the ending dinucleotides was specified, and no restriction of the G/C content was specified. Occurrences of 4 or more identical nucleotides in a row were allowed.

*Jack Lin's siRNA Sequence Finder (Cold Spring Harbor Laboratory) *[65]. We used the full-length mRNA sequence as the input. The spacer length was set as to be 19.

*siDESIGN Center (Dharmacon, Inc.) *[66]. We used the mRNA sequence as the input. No restriction of the leading sequences was specified. The target region was limited to the ORF (open reading frame), the G/C content range was set as 30–52%, and the patterns "GGG" and "CCC" were excluded. The BLAST filtering option was turned on by default.

*siRNA Target Finder (GenScript Corp.) *[67]. We provided the GenBank accession of the mRNA as the input. The length of siRNA was set to be 19. By default, the G/C content range was set to be between 30% and 60%, and sequence selection region was restricted to the ORF.

*Imgenex sirna Designer (Imgenex Corp.) *[68]. The target mRNA was specified using the GenBank accession. The siRNA length was set to be 19. The parameter "nucleotide target" was set to be 50 by default. The parameter "first nucleotide target for siRNA" was set as "AA". The G/C content range was set to be between 45% and 51%. Occurrences of 4 identical A's or T's in a row, or 3 identical (C/G)'s in a row were not allowed. By default, the BLAST search was not performed.

*EMBOSS siRNA (Institute Pasteur) *[69]. We used the full-length mRNA sequence as the input. By default, no restriction of the leading or ending dinucleotides was specified. Occurrences of 4 identical nucleotides in a row were allowed.

*IDT RNAi Design (SciTools) (Integrated DNA Technologies, Inc.) *[70]. The mRNA sequence was provided as the input, and the "21mer" option was selected. The "Unified RNAi Rule Set" was used in the design. The G/C content range was set to be between 30% and 70%. The asymmetrical end stability base pair length was set to be 5. The 5' antisense asymmetrical end stability weight was set to be 0.5, and the 3' overhang was set to be "TT" by default. The default setting was also used for all motifs preferences.

*BLOCK-iT RNAi Designer (Invitrogen Corp.) *[71]. We provided the mRNA sequence as the input. By default, the search in the target region was limited to the ORF. The minimum/maximum allowed G/C contents were set to be 35% and 55%, respectively. The BLAST search option was turned on by default.

*siSearch (Karolinska Institutet) *[72]. We provided the mRNA sequence as the input. By default, the G/C content range was set to be between 30% and 60%. The candidate sites with scores of 6 or above were obtained. The minimum energy difference between two ends of the siRNA was set to be 0. Occurrences of 4 (A/U)'s in a row were not allowed, and the siRNAs containing immunostimulatory motifs were removed. The repeat masking was turned on by default.

*SiMAX (MWG-Biotech, Inc.) *[73]. We used the Genbank accession to specify the target. By default, occurrences of > 3 identical nucleotides in a row in the siRNA sequences, or U's at the 3' end were not allowed. The G/C content range was set to be between 30% and 53%. The search range was restricted to the region between the 100th nucleotide downstream of the start codon and the 100th nucleotide upstream of the end codon. By default, BLAST filtering or secondary structure analysis was not performed.

*BIOPREDsi (Novartis Institutes for BioMedical Research) *[74]. We used the mRNA sequence as the input. The number of predicted siRNAs was set to be 10.

*Promega siRNA Target Designer (Promega Corp.) *[75]. We used the mRNA sequence as the input. The RNAi system was set to be the "T7 RiboMAX Express RNAi system". By default, the target length was set to be 19, and the search region was set to be the whole input sequence.

*QIAGEN siRNA Design Tool (QIAGEN, Inc.) *[76]. We specified the mRNA sequence as the input. The option "Start siRNA sequence with AA" was turned on by default. The BLAST search was not performed.

*SDS/MPI (University of Hong Kong) *[77]. We used the full-length mRNA sequence as the input. The option "MPI Principles" was selected. The filtering of ineffective siRNAs based on secondary structures was not performed. By default, the G/C content range was set to be between 30% and 70%, and the search region was restricted to ≥ 100 nucleotides downstream of the CDS.

*Whitehead WI siRNA Selection Program (Whitehead Institute for Biomedical Research) *[78]. We used the mRNA sequence as the input. By default, the sequence pattern "AAN19TT" was searched for. The G/C content range was set to be between 30% and 70%. Occurrences of 4 or more identical T's, A's or G's in a row were not allowed. Occurrences of 7 or more consecutive (G/C)'s in a row were also not allowed. By default, the checking with BLAST was not performed.

The performance of a siRNA design rule set, or an online siRNA design tool, can be assessed by several parameters. Two of the most often used ones are specificity and sensitivity, as illustrated in Table 5. Specificity is defined as N_D_/(N_B_+ N_D_); and sensitivity is defined as N_A_/(N_A_+ N_C_). An ROC (Receiver Operative Characteristic) curve can be used to visually depict the overall performance of a rule set. The ROC curve is the plot of sensitivity vs. (1-specificity). Another parameter is the positive predictive value (PPV), defined as N_A_/(N_A_+ N_B_). The PPV is a very important parameter in siRNA design practice, because it describes out of the siRNAs predicted to be effective, how big proportion turn out to be truly effective. The value (1-PPV) is sometimes called the "false positive rate".

## Authors' contributions

WG carried out most of the analyses, drafted some proportions of the manuscript and the supplementary text, and helped YR with the construction of the siDRM server. YR worked together with WG to design and implement the siDRM server, and participated in the data compilation and pre-processing work. QX, YW, DL and HZ participated in data compiling and pre-processing work. TL designed the project, carried out some analyses, drafted some proportions of the manuscript and supplementary text, and improved and finalized the writing. All authors read and approved the final manuscript.

## Supplementary Material

Additional File 1 Supplementary results and discussions. Discussions of cooperativity between features in their joint effects, performance of DRM rule sets in subsets divided by confounding factors, utility of online siRNA design tools, rationale of DRM procedure, and survey results of features significant associated with high siRNA efficacy. Click here for file
