# Supplementary material for: Integrated siRNA design based on surveying of features associated with high RNAi effectiveness
Source: BMC Bioinformatics. 2006 Nov 27;7:516. doi: 10.1186/1471-2105-7-516 (PMC1698580; doi:10.1186/1471-2105-7-516)
Supplement: Additional File 1 — Supplementary results and discussions. Discussions of cooperativity between features in their joint effects, performance of DRM rule sets in subsets divided by confounding factors, utility of online siRNA design tools, rationale of DRM procedure, and survey results of features significant associated with high siRNA efficacy. [file 1471-2105-7-516-S1.pdf]

Supplementary material for

Integrated siRNA design based on surveying of features associated with high RNAi effectiveness

Wuming Gong, Yongliang Ren, Qiqi Xu, Yejun Wang,

Dong Lin, Haiyan Zhou and Tongbin Li*

Department of Neuroscience, University of Minnesota, Minneapolis, MN 55455

*To whom correspondence should be addressed.

Email: toli@biocompute.umn.edu.

Tel: 612-626-3481

Fax: 612-626-5009

Word count: 15,906

Supplementary Discussion

1. Cooperativity between features in their joint effects

Here, we derive how interaction, or cooperativity, between features influences the chances of achieving higher efficacies. Without loss of generality, we consider how the chance of achieving >90% efficacies, P_{90}, are affected by a pair of features – F_1 and F_2 – both of which significantly increase the chance of achieving >90% efficacies. We look at three scenarios: (*a*) the two features are independent of each other; (*b*) the two features have positive cooperativity; and (*c*) the two features have negative cooperativity.

Suppose in the general population of siRNA experiments, the chance or achieving >90% efficacies is $P_{90}^{(0)}$. In the subpopulation of experiments carrying F_1, the chance of achieving >90% efficacies is $P_{90}^{(1)}$, and in the subpopulation of experiments carrying F_2, the chance of achieving >90% efficacies is $P_{90}^{(2)}$. Let

$$p_1 = P_{90}^{(1)} - P_{90}^{(0)}, \tag{1.1}$$

and

$$p_2 = P_{90}^{(2)} - P_{90}^{(0)}. \tag{1.2}$$

The chance of achieving >90% efficacies is boosted by

$$s_1 = \frac{P_{90}^{(1)}}{P_{90}^{(0)}} = \frac{P_{90}^{(0)} + p_1}{P_{90}^{(0)}} = 1 + \frac{p_1}{P_{90}^{(0)}} \tag{1.3}$$

times by F_1, and the chance of achieving >90% efficacies is boosted by

$$s_2 = \frac{P_{90}^{(2)}}{P_{90}^{(0)}} = \frac{P_{90}^{(0)} + p_2}{P_{90}^{(0)}} = 1 + \frac{p_2}{P_{90}^{(0)}} \tag{1.4}$$

times by F_2. If F_1 and F_2 are independent of each other (having no cooperativity), then the chance of achieving >90% efficacy should be boosted by

$$s_{12} = s_1 s_2 = (1 + \frac{p_1}{P_{90}^{(0)}})(1 + \frac{p_2}{P_{90}^{(0)}}) = 1 + \frac{p_1}{P_{90}^{(0)}} + \frac{p_2}{P_{90}^{(0)}} + \frac{p_1 p_2}{(P_{op}^{(0)})^2} \tag{1.5}$$

times. When $p_1 << P_{op}^{(0)}, p_2 << P_{90}^{(0)}$ (for our dataset, $P_{90}^{(0)} = 0.34$, and p_1, p_2 are about 0.02-0.03), the last term $\frac{p_1 p_2}{(P_{op}^{(0)})^2}$ can be omitted. Therefore,

$$s_{12} \approx 1 + \frac{p_1}{P_{90}^{(0)}} + \frac{p_2}{P_{90}^{(0)}}, \tag{1.6}$$

and the chance of achieving >90% efficacies is

$$P_{90}^{(12)} = P_{90}^{(0)} \bullet s_{12} \approx P_{90}^{(0)}(1 + \frac{p_1}{P_{90}^{(0)}} + \frac{p_2}{P_{90}^{(0)}}) = P_{90}^{(0)} + p_1 + p_2. \tag{1.7}$$

In other words, F_1 and F_2 are *additive* in their joint effect in boosting the chance of achieving >90% efficacies.

If F_1 and F_2 have positive cooperativity, then we have

$$s_{12} > s_1 s_2 \approx 1 + \frac{p_1}{P_{90}^{(0)}} + \frac{p_2}{P_{90}^{(0)}}, \qquad (1.8)$$

and

$$P_{90}^{(12)} > (P_{90}^{(0)} + p_1 + p_2). \qquad (1.9)$$

In other words, the combined effect of the two features exceeds the sum of the individual effects of the two features. In contrast, if F_1 and F_2 have negative cooperativity, then we have

$$P_{90}^{(12)} < (P_{90}^{(0)} + p_1 + p_2). \qquad (1.10)$$

In this case, the combined effect of the two features is less than the sum of the effects of the two individual features.

Note about the sigmoid shape of the ascending curves in the analysis of feature combinations (Figure 3A and B)

Consider that there are *n* significant features, $F_1, F_2, ..., F_n$. For simplicity, we assume that each of these features leads to equal amount of increase in the chance of achieving >90% efficacy, that is, $p_1 = p_2 = ... = p_n = p$. Following derivations similar to shown above, we can see easily that if these features are independent of each other (showing no cooperativity), then the co-presence of any two features will lead to 2*p* amount of increase in the chance of achieving >90% efficacy; the co-presence of any three features will lead to 3*p* amount of increase in the chance of achieving >90% efficacy; and so on. This will result in a linear relationship between (%records achieving >90% efficacy) and *l*, as illustrated in Supplementary Figure 2 (dotted lines with arrowheads). What we actually see, however, are sigmoid curves (Figure 3A and 4B in the main text, the latter copied to Supplementary Figure 2). At the earlier phase, the curve goes steeper and steeper with increasing *l*, indicating that the features selected had positive cooperativity. At the later phase, the slope decreases and the curve flattens, apparently due to the approaching and reaching of the maximal percentage (100%).

There is an alternative explanation to this observation. Because, in fact, different features did not lead to the same amount of increase in the chance of achieving >90% efficacy when present alone, the increasing slope at the earlier phase of the curve could be due to more features of higher *p*'s being included in the feature combinations. If this is so, we would expect to see that features with higher *p*'s appearing more frequently for higher *l*. However, this is not the case. Features with different *p* ranges are rather equally spread for different *l*'s (results not shown). Therefore, the increasing slope in the early phase of the sigmoid curve is likely due to positive cooperativity among the features.

2. Survey of features significant associated with high siRNA efficacy

In this section we discuss the significant features found in our survey with the three statistical tests: the Wald test of monotone trend, and the odds ratio permutation tests for two different efficacy levels (>70% and >90%), respectively. We loosely call a feature "significant" if the P value of one of the three tests was below 0.05. The false discovery

rate (FDR)[1] was controlled at slightly different levels for the three tests at this P value threshold: for the Wald test of monotone trend, FDR was controlled at 0.18; for the odds ratio permutation tests for >70% and >90% efficacies, the FDR was controlled at 0.15 and 0.16, respectively. By using a loose P value cut-off, more extensive comparisons could be made between our results and previous findings. The FDR could be controlled much more effectively if a lower P value cut-off level. For instance, at P value threshold 0.01, the FDR for the three tests could be controlled at the level of 0.056, 0.044 and 0.038, respectively. This higher stringency level was forced when we selected features for the "feature combination analyses" for the purposes of finding effective siRNA design rule sets.

Category 1: direct sequence features

For the 1st nucleotide of siRNA sequence (throughout this study, we specify nucleotide positions on the sense strand, counting from the 5' end), Takasaki et al. suggested that nucleotide A and U had a lower probability of occurrence and nucleotide G had a higher probability of occurrence than other nucleotides in functional siRNAs[2]. Amarzguioui and Prydz also suggested that absence of nucleotide U at position 1 was strongly correlated with functionality[3]. In our analysis, there was evidence that both features *1st nucleotide ≠U* and *1st nucleotide=G* significantly boosted the chance of achieving >70% efficacies (P_{70} = 0.0014 and 0.0024, respectively), and they were associated with significant up-shift of efficacy distribution (P_{wald} = 0.0495 and 0.022, respectively). We found no evidence that the absence of nucleotide A at position 1 was associated with significant up-shift of the efficacy distribution (P_{wald} = 0.32) or significantly boosted the chance of achieving higher efficacies (P_{70} = 0.12, P_{90} = 0.54).

For the 3rd nucleotide of siRNA sequence, Reynolds et al. suggested that nucleotide A at this position was correlated with siRNA functionality[4]. However, we found no evidence that *3rd nucleotide=A* was associated with significant up-shift of the efficacy distribution (P_{wald} = 0.18), or boosted the chances of achieving >70% or >90% efficacies (P_{70} = 0.32, P_{90} = 0.21). Our results suggested that the super-feature of this feature *3rd nucleotide ≠G* can significantly boost the chance of achieving >90% efficacy (P_{90} = 0.018).

For the 6th nucleotide of siRNA sequence, two groups of researchers suggested that nucleotide A at this position was a positive determinant of siRNA functionality[2, 3]. Hsieh et al. suggested that there was a selection against C at this position for functional siRNA[5]. Our analyses indicated that both features *6th nucleotide ≠C* and *6th nucleotide=A* were associated with significant up-shift of the efficacy distribution (P_{wald} = 0.0052 and 0.0058, respectively), and they significantly boosted the chances of reaching >70% and >90% efficacies (P_{70} = 0.00066 and 0.0024; P_{90} = 0.0089 and 0.0058, respectively). Moreover, our results suggested that the feature *6th nucleotide=U,* the sub-feature of 6th nucleotide ≠C, and the feature *6th nucleotide ≠G,* the super-feature of *6th nucleotide=A,* were associated with significant up-shift of the efficacy distributions (P_{wald} = 0.042 and P_{wald} = 0.018 respectively), and boosted the chance of reaching >90% efficacy (P_{90} = 0.035 and P_{90} = 0.018 respectively). The feature *6th nucleotide ≠G* was also found to boost the chance of achieving >70% efficacy (P_{70} = 0.048).

For the 7th nucleotide of siRNA sequence, Takasaki et al. suggested that nucleotide U had a lower probability of occurrence, and nucleotide G had a higher probability of occurrence in functional siRNAs[2]. Our results indicated that *7th nucleotide ≠U* was associated with significant up-shift of efficacy distribution (P_{wald} = 0.0091) and elevated the chance of achieving >70% (P_{70} = 0.010) and >90% (P_{90} = 0.0043) efficacies. The feature *7th nucleotide=G*, though, was not found to be significant (P_{70} = 0.069, P_{90} = 0.32, P_{wald} = 0.17). Our result also indicated that the feature *7th nucleotide=A,* which is the sub-feature of *7th nucleotide ≠U*, was associated with the up-shift of the efficacy distribution (P_{wald} = 0.016), and elevated the chance of reaching >90% efficacy (P_{90} = 0.0014).

For the 8th nucleotide of siRNA sequence, Takasaki et al. suggested that nucleotide A had a higher probability of occurrence and nucleotide G had a lower probability of occurrence in functional siRNAs[2]. In our analysis, the feature *8th nucleotide=A* was not found to be associated with significant up-shift of the efficacy distribution (P_{wald} = 0.27) or boosted the chances of achieving higher siRNA efficacies (P_{70} = 0.12, P_{90} = 0.77). Instead, we found the feature *8th nucleotide=G* significantly boosted the chance of achieving >90% efficacy (P_{90} = 0.0054), contrary to the observation by Takasaki et al. Moreover, we found that the feature *8th nucleotide ≠C*, the super-feature of the features *8th nucleotide=A* and *8th nucleotide=G*, significantly boosted the chance of achieving >90% efficacy (P_{90} = 0.023).

For the 9th nucleotide of siRNA sequence, Takasaki et al. suggested that nucleotide U has a higher probability and nucleotide G has a lower probability of occurrence in functional siRNA sequences[2]. We found no evidence that the feature *9th nucleotide ≠G* was significant (P_{70} = 0.68, P_{90} = 0.36, P_{wald} = 0.48). Instead, the feature *9th nucleotide ≠U* was found to be significant in elevating the chance of achieving >90% efficacy (P_{90} = 0.035). We also found the feature *9th nucleotide=C*, the sub-feature of two above features *9th nucleotide ≠G* and *9th nucleotide ≠U*, was associated with significant up-shift of the efficacy distribution (P_{wald} = 0.00053), and was strongly associated with >70% and >90% efficacies (P_{70} = 0.0080 and P_{90} = 0.00021). Another feature *9th nucleotide ≠A*, was associated with up-shift of efficacy distribution (P_{wald} = 0.021), and boosted the chance of reaching >70% efficacy (P_{70} = 0.0061).

There were disagreements in previous studies about how the occurrence of nucleotide U at position 10 influences siRNA functionality. Reynolds et al. suggested that a U at position 10 was associated with higher efficacies[4], whereas Amarzguioui and Prydz showed that the nucleotide U at position 10 was associated with lack of functionality[3]. We found no evidence that either the feature *10th nucleotide=U* or *10th nucleotide ≠U* was associated with significant up-shift of the efficacy distribution, or elevated the chances of achieving higher efficacies (P_{70} = 0.20, P_{90} = 0.062, P_{wald} = 0.13 for the feature *10th nucleotide=U*; P_{70} = 0.80, P_{90} = 0.94, P_{wald} = 0.87 for the feature *10th nucleotide ≠U*).

For the 11th nucleotide of siRNA sequence, Hsieh et al. suggested that there was a negative selection against nucleotide A and preference for nucleotide C at this position for functional siRNAs[5]. However, in our analyses, there was no evidence that feature *11th nucleotide≠A* was associated with significant up-shift of efficacy distribution (P_{wald} 0.86) or boosted the efficacies to higher levels (P_{70} = 0.86, P_{90} = 0.86). Moreover, the feature *11th nucleotide≠C* was found to slightly boost the chance of achieving >90% efficacies (P_{90} = 0.029).

For the 13th nucleotide of siRNA sequence, Hsieh et al. suggested that there was enrichment for nucleotide A at this position for functional siRNA[5]. Amarzguioui and Prydz suggested that nucleotide U at position 13 was a positive determinant of siRNA functionality[3]. Reynolds et al. suggested that the absence of nucleotide G at position *13* contributed to the siRNA functionality[4]. Our analysis showed that the feature *13th nucleotide=A* was associated with significant up-shift of efficacy distribution (P_{wald} = 0.020) and significantly elevated the chance of achieving >70% efficacy, and slightly boosted the chance of achieving >90% efficacies (P_{70} = 0.008, P_{90} = 0.052). There was no evidence that other two features *13th nucleotide=U* and *13th nucleotide ≠G* was associated with significant up-shift of distribution (P_{wald} = 0.71 for the feature *13th nucleotide=U;* P_{wald} = 0.31 for the feature *13th nucleotide ≠G*), or elevated the chances of achieving high efficacies (P_{70} = 0.84, P_{90} = 0.64 for the feature *13th nucleotide=U;* P_{70} = 0.43, P_{90} = 0.29 for the feature *13th nucleotide ≠G*).

For the 15th position of siRNA nucleotide, Takasaki et al. indicated that nucleotide U has a higher probability of occurrence than other nucleotides in this position of functional siRNA sequences[2]. However, in our analysis, there was no evidence that *15th nucleotide=U* was associated with significant up-shift of the efficacy distribution (P_{wald} = 0.10), or boosted the chances of achieving higher efficacies (P_{70} = 0.23, P_{90} = 0.10). Rather, our results suggested that the super-feature *15th nucleotide ≠C*, and the feature *15th nucleotide=G*, both boosted the chances of achieving >70% efficacies (P_{70} = 0.0045 for the feature 15th nucleotide ≠C; P_{70} = 0.010 for the feature *15th nucleotide=G*). The feature *15th nucleotide ≠C* was also associated with up-shift of efficacy distribution (P_{wald} = 0.023).

For the 16th position of siRNA nucleotide, Amarzguioui and Prydz suggested that the presence of nucleotide C at this position was strongly correlated with siRNA functionality[3], and Hsieh et al. suggested that there was enrichment for nucleotide G at this position for functional siRNA[5]. Our analysis indicated that the feature *16th nucleotide=C* significantly boosted the chance of achieving the >70% efficacy (P_{70} = 0.026, yet P_{90} = 0.10, P_{wald} = 0.058). But no evidence was found to support the significance of the feature *16th nucleotide=G* (P_{70} = 0.74, P_{90} = 0.71, P_{wald} = 0.68).

For the 19th position of siRNA nucleotides, Hsieh et al. indicated that there was a strong preference for nucleotide U at position 19 of functional siRNA[5]. Several groups suggested that absence of nucleotide G at this position was strongly correlated with siRNA functionality[2, 3, 5]. Our result agreed well with the widely accepted selection against G at position 19 – the feature *19th nucleotide ≠G* was associated with a

significant up-shift of efficacy distribution (P_{wald} = 0.0012) and strongly boosted the chances of achieving >70% and >90% efficacies (P_{70} = 0.00049, P_{90} = 0.0043). There was no evidence that the presence of nucleotide U at position 19 was associated with significant up-shift of efficacy distribution (P_{wald} = 0.086) or elevated the chances of achieving higher efficacies (P_{70} = 0.098, P_{90} = 0.23). Moreover, our result suggested that the feature *19th nucleotide=A*, the sub-feature of *19th nucleotide ≠G,* was associated with significant up-shift of efficacy distribution (P_{wald} = 0.0014), and boosted the chance of reaching both >70% and >90% efficacies (P_{70} = 0.00066 and P_{90} = 0.0054).

Aside from aforementioned features reported in previous studies, the features *2nd nucleotide=A* (P_{70} = 0.010, P_{90} = 0.0026, P_{wald} = 0.0019), *5th nucleotide=A* (P_{70} = 0.0080, P_{90} = 0.0023, P_{wald} = 0.010), *5th nucleotide ≠G* (P_{70} = 0.032, P_{90} = 0.023, P_{wald} = 0.035), *12th nucleotide=G* (P_{70} = 0.013, P_{90} = 0.023, P_{wald} = 0.027) and *18th nucleotide ≠C* (P_{70} = 0.010, P_{90} = 0.00071, P_{wald} = 0.0048) were found to be associated with significant up-shift of the efficacy distribution and significantly boosted the chances of achieving >70% and >90% efficacies. The features *4th nucleotide=C* (P_{90} = 0.00036, P_{wald} = 0.0075), *14th nucleotide ≠C* (P_{90} = 0.00053, P_{wald} = 0.019) and *17th nucleotide ≠C* (P_{90} = 0.012, P_{wald} = 0.044) were found to be associated with significant up-shift of the efficacy distribution and boosted the chance of achieving >90% efficacies. The features *17th nucleotide=A* (P_{70} = 0.00049, P_{wald} = 0.0049), *17th nucleotide ≠G* (P_{70} = 0.0018, P_{wald} = 0.041) and *18th nucleotide=A* (P_{70} = 0.048, P_{wald} = 0.041) were found to be associated with significant up-shift of the efficacy distribution and significantly boosted the chance of achieving >70% efficacies. In addition, the feature *5th nucleotide ≠U* (P_{70} = 0.026) significantly boosted the chance of achieving >70% efficacies, and the features *4th nucleotide ≠U* (P_{90} = 0.043) and *14th nucleotide=U* (P_{90} = 0.035) significantly boosted the chance of achieving >90% efficacies.

Category 2: sequence derived features

It was suggested by several groups that G/C was positive determinant of siRNA functionality at this position[3, 6, 7]. In our analysis, we found evidence that the feature *1st nucleotide=(G/C)* was associated with significant up-shift of the efficacy distribution (P_{wald} = 0.049) and significantly elevated the chance of achieving >70% efficacy (but not for >90% efficacy) (P_{70} = 0.0061, P_{90} = 0.43).

For the 10th nucleotide of siRNA sequence, Jagla et al. suggested that A/U at this position was related to siRNA functionality[6]. We found no evidence that *10th nucleotide=A/U* was associated with significant up-shift of the efficacy distribution (P_{wald} = 0.19), or elevated the chances of achieving higher efficacies (P_{70} = 0.54, P_{90} = 0.062).

For the 11th nucleotide of siRNA sequence, Hsieh et al. suggested that there was a strong preference for G/C at this position for functional siRNA[5]. However, we found no evidence that the feature *11th nucleotide=(G/C)* was associated with up-shift of the efficacies distribution (P_{wald} = 0.92), or elevated the chances of achieving higher efficacies (P_{70} = 0.80, P_{90} = 0.88).

For the 19th nucleotide of siRNA sequence, Several groups suggested that A/U at this position was associated with siRNA functionality[3, 6, 7]. This observation was confirmed by our analysis. The feature *19th nucleotide=(A/U)* significantly boosted the chance of achieving >70% and >90% efficacies (P_{70} = 0.00029, P_{90} = 0.0043) and was strikingly associated with an up-shift of the efficacy distribution (P_{wald} = 0.000058).

Ui-Tei et al. suggested that in highly effective siRNA, at least five (A/U)s should be contained in the 3' end one-third of the sense strand[7]. Jagla et al. suggested that more than three (A/U)s between position 13 and 19 was critical for siRNA functionality[6]. Reynolds et al. suggested that the occurrence of three or more (A/U)s in nucleotides 15-19 could be a criterion for selecting functional siRNA[4]. Our results indicated that both features *at least three (A/U)s in the seven nucleotides at the 3' end* and *at least three (A/U)s in the five nucleotides at the 3' end* were strongly associated with an up-shift of the efficacy distribution (P_{wald} = 2.5E-9, and 0.000022, respectively), in addition to strongly boosted the chances of achieving both >70% (P_{70} = 0.00001, and 0.0018, respectively) and >90% efficacies (P_{90} = 0.00001, and 0.00016, respectively). There was evidence that the feature *at least five (A/U)s in the seven nucleotides at the 3' end* significantly boosted the chance of achieving >70% efficacy (P_{70} = 0.026), but no evidence was found that this feature boosted the chance of >90% efficacy (P_{90} = 0.26), or was associated with up-shift of the efficacy distribution (P_{wald} = 0.10).

About long G/C stretches and siRNA functionality, two groups suggested that *siRNAs with G/C stretches longer than 9* should be excluded for their lack of functionality[7, 8], and another two groups suggested that *no occurrences of G/C stretches of length 7 or longer* should not be allowed in siRNA design[9, 10]. Our results indicated that, indeed, the feature *no occurrences of G/C stretches of length 7 or longer* significantly boosted the chances of achieving >70% and >90% efficacies (P_{70} = 0.00001, P_{90} = 0.00001), and were associated with significant up-shifts of the efficacy distribution (P_{wald} = 0.000015). The feature *no occurrences of G/C stretches of length 9 or longer* was not tested because too few records (<30) carried this feature in our dataset.

About stretches of identical nucleotides and siRNA functionality, Several groups suggested that consecutive 3 or 4 identical nucleotides should be avoided in siRNA design to reduce the RNA duplex internal stability[9-13]. In our analysis, both two features *no occurrences of three or more identical nucleotides in a row and no occurrences of four or more identical nucleotides in a row* were found to be associated with a significant up-shift of the efficacy distribution (P_{wald} = 0.0067, and 0.0014, respectively), in addition to significantly boost the chances of achieving >70% (P_{70} = 0.0061, and 0.00001, respectively) and >90% efficacies (P_{90} = 0.043, and 0.012, respectively).

About G/C content and siRNA functionality, seven different G/C content ranges reported in previous studies were tested: 30 – 52% [4], 32 – 79% [14], 30 – 70% [15], 35 – 60% [9], 20 – 50% [13], 31.6 – 57.9% [3] and 30 – 79% [11]. Our results indicated that the G/C content ranges 35 – 60%, 31.6 – 57.9% and 30 – 70% were associated with significant up-shifts of the efficacy distribution (P_{wald} = 0.00018, 0.00018, and 0.00028,

respectively), and significantly boosted chances of achieving > 70% (P_{70} = 0.00001 for all three features) and >90% efficacies (P_{90} = 0.0019, 0.0019, and 0.00001, respectively). The feature *G/C content is between 20 and 50%* had a weaker, yet still significant effect in associating with up-shift of the efficacy distribution (P_{wald} = 0.037).

Category 3: thermodynamic features

About 5' end binding energy and siRNA efficacy, Chalk et al. suggested that *sense 5' binding energy between -9 and-5 Kcal/Mol* was associated with higher siRNA efficacies[16]. We tested this feature but found no evidence that it was associated with an up-shift of the efficacy distribution or boosted the chances of achieving higher efficacies (P_{70} = 0.71, P_{90} = 0.90, P_{wald} = 0.87).

About mid-sequence binding energy, Khvorova et al. suggested that *lower internal energy (N6 – N11)* was strongly associated with higher siRNA functionality[17]. Chalk et al. suggested that the feature *binding energy of N7-N12 > -13 KCal/Mol* was strongly associated with higher siRNA efficacy[16]. Poliseno et al. made similar observations, and suggested that the energy of N7-N11 was correlated with functional siRNA[18]. We found that the feature *binding energy of N7-N12 ≤ -13KCal/Mol* significantly boosted the chance of achieving >70% efficacy (P_{70} = 0.032, yet P_{90} = 0.10, P_{wald} = 0.054). However, we did not find any evidence that the features *binding energy of N6-N11 < -13 KCal/Mol* and *mean of free energy profile of N7-N11 is between 1.97 and -1.65 KCal/Mol (inclusive)* associated with higher siRNA efficacy distribution, or higher chances of achieving >70% or >90% efficacies (P_{wald} = 0.62, P_{70} = 0.46, P_{90} = 0.61 for the feature *binding energy of N6-N11 < -13 KCal/Mol*; P_{wald} = 0.32, P_{70} = 0.46, P_{90} = 0.43 for the feature *mean of free energy profile of N7-N11 is between 1.97 and -1.65 KCal/Mol (inclusive)*).

About 3' end binding energy and siRNA functionality, Chalk et al. suggested that the feature *binding energy of N16-N19 > -9 KCal/Mol* was associated with higher siRNA effectiveness[16]. We found that indeed, this feature was associated with a significant up-shift of the efficacy distribution (P_{wald} = 0.0025), and boosted the chance of achieving >70% and >90% efficacies (P_{70}= 0.010, P_{90} = 0.0026).

About binding energy difference between the 3' end and the 5' end of the siRNA, Khvorova et al. suggested that 5' terminal of anti-sense strand had enhanced flexibility for functional siRNA[17]. Poliseno et al. suggested that for functional siRNA the five terminal nucleotides of 5' end of the anti-sense strand had a higher free energy than that of 5' end of sense strand[18]. Chalk et al. observed that a siRNA was more effective if the free energy of 5' end of anti-sense strand was higher than that of 5' side of sense strand, and if their difference was less than 1 KCal/Mol[16]. Our analysis indicated that the feature *binding energy of N16-N19 ≥ binding energy of N1-N4* was associated with a significant up-shift of the efficacy distribution (P_{wald} = 0.0043), and boosted the chances of achieving >70% and >90 efficacies significantly (P_{70} = 0.013, P_{90} = 0.018). The feature *binding energy of N16-N19– binding energy of N1-N4 is between 0 and 1 KCal/Mol* showed even higher levels of significance (P_{wald} = 0.010, P_{70} = 0.00036 and P_{90} = 0.0078). These results agreed with the observation by Chalk et al. very well. However, when one more nucleotide was included in the calculations of terminal energy,

the evidence for significance weakened. The feature *binding energy of N15-N19 > binding energy of N1-N5* was not found to be associated with an up-shift of the efficacy distribution (P_{wald} = 0.12) or higher chances of achieving >70% or >90% efficacies (P_{70} = 0.20, P_{90} = 0.26).

About internal folding potential and siRNA functionality, Wang and Mu suggested that functional siRNA sequences should have minimum free energy higher than -5 KCal/Mol[9]. Chalk et al. observed that absolute value of the total hairpin energy need to be less than 1 KCal/Mol for functional siRNAs[16]. Reynolds et al. found there was no functional siRNA with T_m > 60°C in their data set and suggested that T_m < 20°C was a feature associated with higher siRNA efficacies[4]. Our analysis indicated that the feature *folding energy of sense strand ≥ -5 KCal/Mol* was associated with a significant up-shift of the efficacy distribution (P_{wald} = 0.023), and strongly boosted the chances of achieving >70% and >90% efficacies (P_{70} = 0.00004, P_{90} = 0.00002). There was no evidence found to associate the feature *absolute value of total hairpin energy < 1 KCal/Mol* with higher siRNA efficacies (P_{70} = 0.18, P_{90} = 0.23, P_{wald} = 0.19). The feature *T_m < 60°C* was found to be associated with significant up-shift of efficacy distribution (P_{wald} = 0.0057), and significantly boosted chance of achieving >70% efficacy (P_{70} = 0.0014). However, no evidence was found to associate the feature *T_m < 20°C* with higher efficacies. Instead, the complementary feature of this feature (*Tm ≥ 20°C*) was found to boost the chance of achieving >90% efficacy (P_{90} = 0.0026). Additionally, our results suggested that the feature *Tm is between 20 and 60°C* was associated with a significant up-shift of the efficacy distribution (P_{wald} = 0.003), and boosted the chance of achieving >70% and >90% efficacies (P_{70} = 0.0045, P_{90} = 0.023).

Category 4: features defined based on target mRNA sites

About the location of the siRNA target site on the mRNA, it was suggested that the first 100 nucleotides of CDS, 5' UTR and 3'UTR should not be targeted by siRNAs since they may contain regulatory protein binding sites[11, 14], an argument agreed with by Wang and Mu, who suggested that only the CDS region be used when designing siRNA experiments[9]. However, Hsieh et al. observed that siRNAs targeting the 3'UTR were equally effective as siRNAs targeting the CDS[5]. In addition, they observed that siRNAs targeting outside of the third quartile of CDS yielded higher knockdown effectiveness. In our analysis, three features *target site is not on the 5'UTR*, *target site is not on the 3'UTR* and *target site is on CDS* were found to quite strongly boost the chances of achieving both >70% and >90% efficacies (P_{70} = 0.00001 and P_{90} = 0.00001 for three features), and associate with significant up-shift of efficacy distribution (P_{wald} = 0.016, 0.00052, and 0.000055, respectively). These results generally agreed with previous observations[9, 11, 14]. Contrary to the observation made by Hsieh et al., we found the target site on the 4th quartile rather than on the 3rd quartile of CDS had negative effect for siRNA functionality. The feature *target site is not on the 4th quartile of CDS* quite strongly boosted the chance of achieving >70% efficacy (P_{70} = 0.00015, yet P_{90} = 0.088, P_{wald} = 0.025). In addition, we observed that when the target site was on the first three quartiles, RNAi achieved significantly higher efficacies. Indeed, feature *target site is on the 3rd quartile of CDS* had significant effect in boosting the chance of achieving >70% and >90% efficacies (P_{70} = 0.017, P_{90} = 0.029), and associating with up-

shift of efficacy distribution (P_{wald} = 0.031), while feature *target site is on the 1st quartile of CDS* and *target site is on the 2nd quartile of CDS* significantly boosted the chance of achieving >70% (P_{70} = 0.0024), and >90% (P_{90} = 0.023) efficacy, respectively. The odds ratio permutation tests and Wald test did not yield significant determinants for feature *target site is on the first 100 nucleotides of CDS* (P_{70} = 0.82, P_{90} = 0.57, P_{wald} = 0.59).

About the features on accessibility of mRNA region targeted by siRNA, Scheer et al. suggested that mRNA region gaining high accessibility score was related to high cleavage efficacy[19]. Ding et al. suggested that the anti-sense siRNA binding energy should be less than -10 KCal/Mol for functional siRNA[12]. Yiu et al. suggested functional siRNA should pass their filtering algorithm that filtered out mRNA target region deemed as inaccessible[20]. Schubert et al. suggested that the siRNA silencing efficacy was positively correlated with local energy of target structure which was measured by LFE[21]. Luo and Chang suggested that H-b index was highly correlated with the gene-silencing efficiency of siRNA[22]. In our analysis, the feature *Anti-sense siRNA binding energy ≤ -10 KCal/Mol* significantly boosted the chance of achieving >70% efficacy (P_{70} = 0.026, yet P_{90} = 0.23, P_{wald} = 0.072). The feature *LFE_mss ≥ -20.9 KCal/Mol* was found to be associated with a weak, yet significant up-shift of efficacy distribution (P_{wald} = 0.048). The feature *H-b index < 28.8* was found to significantly boost the chance of achieving >70% efficacy, as well as associate with up-shift of efficacy distribution (P_{70} = 0.032, P_{wald} = 0.012, yet P_{90} = 0.12). But odds ratio permutation tests and Wald test did not yield significant determinations for feature *Accessibility score > 0* (P_{70} = 0.57, P_{90} = 0.43, P_{wald} = 0.37). The feature *Does not pass Repelling Loop Filter* was found to significantly boost the chance of achieving >90% efficacy (P_{90} = 0.010), contrary to previous observation. The feature *Local folding potential (mean) ≥ -22.72 KCal/Mol* was found to strongly elevate the chance of reaching >70% and >90% efficacies (P_{70} = 0.00001, P_{90} = 0.00001), and associate with very significant up-shift of efficacy distribution (P_{wald} = 9.3E-09). These observations generally agreed with previous suggestions that the accessibility of mRNA region targeted by siRNA influenced RNAi effectiveness.

Category 5: features based on experimental settings

Several groups reported that efficiency of transfection was typically higher for synthetic siRNA than for plasmid DNA[23, 24]. Our survey confirmed the positive effect of synthetic siRNA methods in achieving higher knockdown efficacies. The feature *Transfection method = Synthesized oligos* was found to significantly boost the chance of achieving >70% efficacy (P_{70} = 0.026), and associate with significant up-shift of efficacy distribution (P_{wald} = 0.028).

It is known that cell line types were correlated with siRNA efficacies. Several groups showed that the transfectability of cells is the limiting step in siRNA mediated gene silencing and differs between different cell types[25-27]. For example, HeLa cells were well-known for their ease of transfection[24], and primary cells had lower transfection efficacy than cancer cells[24, 28]. Moreover, there was evidence that the genetic context in each individual tumor cell lines also had effected in RNAi[28, 29]. Our survey indicated that the feature *Cell line = HeLa* strikingly boosted the chances of achieving

>70% and >90% efficacies (P_{70} = 0.00001, P_{90} = 0.00016), and was associated with quite significant up-shift of efficacy distribution (P_{wald} = 4.0E-09). Other four common cell lines HEK293, MCF7, CV-1, and 3T3 had significantly negative effect in achieving higher knockdown percentages (P_{70} = 0.021 for feature *Cell line ≠ HEK293*; P_{90} = 0.00016 for feature *Cell line ≠ MCF7*; P_{70} = 0.0018, P_{90} = 0.00001; P_{wald} = 0.027 for feature *Cell line ≠ CV-1 and derivatives*; P_{70} = 0.026, P_{90} = 0.00001 for feature *Cell line ≠ 3T3*).

It has been reported that the efficacy ratings depended on the test objects (protein or mRNA) and test methods (Western blot, or PCR-related). The turnover of proteins has been implicated in the relationship between knockdown percentage between proteins and mRNAs[30]. In our analysis, there was evidence that when protein levels were tested, the efficacy ratings tended to go significantly higher (P_{70} = 0.00001, P_{90} = 0.00001, P_{wald} = 2.2E-09). On other hand, when mRNA levels were measured, the efficacy ratings were significantly lower (P_{70} = 0.00001, P_{90} = 0.00001, P_{wald} = 9.3E-10 for feature *Test object ≠ mRNA*). Similarly, the feature *Test method = Western blot* led to significantly higher efficacy ratings (P_{70} = 0.00001, P_{90} = 0.00001, P_{wald} = 3.8E-14). The feature *Test method = PCR-related* led to significantly lower efficacy ratings (P_{70} = 0.00001, P_{90} = 0.00001, and P_{wald} = 2.6E-08). The features *Test method ≠ Northen blot* and *Test method ≠ Luciferase assay* were also associated with a significant up-shift of efficacy distribution (P_{wald} = 0.010 and 4.7E-08, respectively), and have very significant chances to achieve >70% and >90% efficacies (P_{70} = 0.00001, and P_{90} = 0.00001, for both features). The feature *Test method = bDNA* was found to have a strong chance to reach >90% efficacy (P_{90} = 0.0019).

3. Performance of DRM rule sets in subsets divided by confounding factors

The factors regarding experimental settings, e.g., *test method* and *test object* are considered as confounding factors for our purpose of developing siRNA design rules, because although they influence the siRNA efficacy, we do not want to include them in the siRNA design criteria, as that would restrict the applicability of the resulting design rules. Yet, the high level of significance of features concerning these factors (*Test method = Western blot* and *Test object ≠ mRNA*) prompted us to examine the performance of the DRM rule sets for the subsets of siRNA experiments separated by these features. Supplementary Figure 4 shows how the PPVs of the four subsets of records carrying the features *Test method=Western blot*, *Test method ≠ Western blot*, *Test object=mRNA* and *Test object ≠ mRNA*, respectively, changed with the stringency level α for the DRM rule sets. It appears that at higher α levels, the DRM rule sets are more effective in selecting good siRNAs for "the Western subset" and "the non-mRNA subset", and less effective for "the non-Western subset" and "the mRNA subset". As α decreases, they become less effective for "the Western subset" and "the non-mRNA subset", but more effective for "the non-Western subset" and "the mRNA subset". The PPVs for the four subsets become roughly equal at α <0.8.

Due to the small sample size problem, this analysis should not be considered as conclusive. Yet, it suggests that DRM rule sets behave differently for subpopulations of

siRNAs tested under different experimental settings. We will examine this issue further as more data becomes available through the *siRecords* effort. When there is enough data, we will try to develop design rule sets for these different subpopulations of siRNAs separately.

4. Utility of online siRNA design tools

In this section we discuss the issue of relative utility of existing siRNA design tools. A large number of siRNA design tools are now available online. It is interesting to assess which of them are used more frequently than others in the current siRNA design practice. Another reason why we look at this issue is that if the current siRNA design practice is dominated by one or two design tools that are most frequently used, the objectiveness of the performance comparison (shown in Table 3) would be compromised. A straightforward way to analyze the utility of these siRNA design tools is to perform a statistics analysis of those original siRNA studies of what design tools that were used in their siRNA design. However, a large proportion of these original studies (~80%) did not have descriptions about what tools were used in their design. Thus, we seek to develop a method of assessing the utility of the design tools approximately by directly analyzing the *siRecords* data.

We assume that there are N siRNA design tools from which a user can pick to help his/her siRNA design, and that the user picks only one tool to assist the design of any single siRNA experiment (this is a simplifying assumption for the ease of discussion – it is conceivable that the user may seek help from multiple design tools in a real siRNA design task). The design tool chosen is used to make predictions of a pool of candidate siRNA sites, and a proportion of the candidate sites that are predicted to be effective by that design tool are chosen to be tested experimentally. It is not hard to conceive that the higher utility a tool possesses, the better chance that a candidate siRNA predicted to be effective by this tool is picked to be tested. Considering that existing design tools have overlaps in their predictions (that is, some siRNA sites are predicted to be effective by multiple design tools), we focus on the "uniquely predicted effective" (or UPE) sites only, i.e. the candidate siRNA sites that are predicted to be effective by only one tool. Thus, we have

$$Utility(i) \propto P(T \mid UPE(i)), \qquad i \in [1, N]. \qquad (4.1)$$

That is, the utility of a design tool, tool i, is proportional to the conditional probability that the UPE sites of this tool are picked to be tested experimentally. By Bayes' Theorem, we have

$$P(T \mid UPE(i)) = \frac{P(UPE(i) \mid T) \cdot P(T)}{P(UPE(i))} \propto \frac{P(UPE(i) \mid T)}{P(UPE(i))}, \qquad (4.2)$$

where $P(T)$, the probability for a candidate siRNA site to be tested, is considered as a constant. From (4.1) and (4.2), we get

$$Utility(i) \propto \frac{P(UPE(i) \mid T)}{P(UPE(i))}. \qquad (4.3)$$

In other words, the utility of a given design tool is proportional to the ratio of the probability that a tested site is a UPE of this tool, and the probability that any site (tested

or untested) is a UPE of the tool. We will call this ratio the "uniquely positive testing ratio", or UPTR.

We estimated the UPTR values of the 15 online siRNA design tools. The Set T data involves 774 genes, on which there are 2,453,510 19-mer candidate sites. Among these candidate sites, 1,014 were experimentally tested. We calculated the number of UPE sites, n_{UPE}^{T}, among the 1,014 tested sites for each siRNA design tool. Then, we randomly picked 10,000 sites from the 2,453,510 candidate site pool, and calculated the number UPE sites for among these 10,000 sites, n_{UPE}. The UPTR of a design tool was calculated as

$$UPTR = \frac{n_{UPE}^{T} / 1014}{n_{UPE} / 10000}. \qquad (4.4)$$

Four online design tools, EMBOSS sirna by Institute Pasteur, SDS/MPI by University of Hong Kong, Ambion siRNA Target Finder by Ambion, Inc. and Jack Lin's siRNA Sequence Finder by Cold Spring Harbor Laboratory had fairly large coverage of predicted effective sites: among the 1,014 tested sites, 765, 642, 564 and 229 sites were predicted to be effective by these tools respectively. This resulted in 0 UPE site found in the tested set for several of the other design tools. To counter this problem, we loosened the definition of UPE for the remaining 11 tools, in that a site was deemed as a UPE if it was predicted to be effective by this tool, but not predicted to be effective by any of the other lower coverage tools. This compromise was deemed proper because we were only making approximate estimate of these tools' relative utility. Without making this compromise, this utility comparison of these tools would not be possible.

Supplementary Table 7 shows the n_{UPE}^{T}, n_{UPE} and the UPTR of the 15 siRNA design tools. Five tools, Imgenex sirna Designer by Imgenex Corp., WI siRNA Selection Program by Whitehead Institute, QIAGEN siRNA Design Tool by QIAGEN, Inc., SiMAX by MWG-Biotech, Inc. and IDT RNAi Design by Integrated DNA Technologies, Inc. had the highest levels of utility (with UPTR between 8 and 18). These tools are followed by siDESIGN Center by Dharmacon, Inc., Promega siRNA Target Designer by Promega Corp., BLOCK-iT RNAi Designer by Invitrogen Corp., BIOPREDsi by Novartis Institutes for BioMedical Research and siRNA Target Finder by GenScript Corp. (with UPTR between 2 and 8), then by siSearch by Karolinska Institutet, Ambion siRNA Target Finder by Ambion, Inc., SDS/MPI by University of Hong Kong, EMBOSS sirna by Institute Pasteur and Jack Lin's siRNA Sequence Finder by Cold Spring Harbor Laboratory (UPTR <2). These results suggest that the current siRNA design practice is not dominated by one or two individual tools; rather, many tools are being used with varied levels of utility.

5. Rationale of the DRM procedure

Finally, we discuss the considerations underlying the development of the procedure (what we term *the DRM procedure*) through which the siRNA design criteria were obtained in this study. In essence, this procedure can be described as follows: First, we construct *rules*, or conjunctions (combinations) of features that lead to strong boosting of siRNA

efficacy; the positive cooperativity between rules is exploited at this step. Second, we merge the rules, remove redundancy, and formulate *rule sets*, or disjunctions of rules, the stringency (or specificity) of which is controlled at prescribed levels. All previous studies in siRNA design criteria focused on the first of these two steps, i.e., constructing conjunctive rules from interesting features. What is the advantage of taking the second step of making disjunctive rule sets? The answer is, simply put, by constructing disjunctive rule sets, while maintaining a good level of specificity, we can achieve a higher level of sensitivity, because sensitivities of all rules in the disjunctive rule set add up to produce the sensitivity of the disjunctive rule set.

Suppose we are looking at a single rule, or a conjunction of l features. As l increases, while the specificity of this rule is increasing (given that the features included in the rule are truly helping the selection of effective siRNAs), the sensitivity of the rule will be decreasing exponentially, because whenever a new feature is added into the conjunction, a proportion of the remaining experiments will fail to carry this new feature. This is demonstrated in Supplementary Figure 5, where the sensitivity and specificity of single conjunctive rules are plotted against the number of features l (also see Figure 3C). We need to consider the balance between the gain in specificity and the loss in sensitivity, to determine if the including of the new feature is worthwhile. When we look at a rule set (or a disjunction of m rules), as m increases, the specificity of the rule sets also decreases; meanwhile the sensitivity of the rule set will increase. The simultaneous changes in specificity and sensitivity with m for the disjunctive rule sets are different from the simultaneous changes in specificity and sensitivity with l for a single conjunctive rule, in that the changes for the rule sets are approximately linear (Supplementary Figure 6); and the slope of the rising curve of the sensitivity is greater than the slope of the falling curve of the specificity. Therefore, generally speaking, the higher m is, the better performance the rule sets will achieve, given that the specificity of each of the m rules is well controlled.

Supplementary Figure 1 A permutation test of odds ratios was used to determine the significance of a feature in its association with higher chances to achieve >70% (and >90%) efficacies. The odds ratio between the feature *the 6th nucleotide=A* and complementary feature *the 6th nucleotide ≠ A* for >70% efficacies (records with efficacy rating "high" or "very high") was 1.289, smaller than 243 of 100,000 odds ratios in the null distribution. Thus, the permutation test rendered P= 243/100,000=0.00243 for >70% efficacies for the feature *the 6th nucleotide=A*.

Supplementary Figure 2 The sigmoid shape of the ascending curves – (%records achieving >90% or >70% efficacy) vs. l relationships – suggests that there is positive cooperativity between features included in the selected feature combinations. The ascending curves would be straight lines (illustrated by dotted lines with arrowheads) if no cooperativity existed among the features.

Supplementary Figure 3 The relationships between the number of effective siRNAs predicted and the gene length, for two DRM rule sets, $RS_{0.951}$ and $RS_{0.845}$, plotted on log-log scale.

Supplementary Figure 4 The PPVs for the entire Set T and the four subsets carrying the features *Test method=Western blot*, *Test method ≠ Western blot*, *Test object=mRNA* and *Test object ≠ mRNA* respectively for 6 DRM rule sets with decreasing α. A siRNA experiment was considered effective if it achieved >70% efficacy (was rated "high" or "very high" efficacy).

Supplementary Figure 5 Plot of sensitivity and specificity of single rules (conjunctions of l features) vs. l. A siRNA experiment was considered effective if it achieved >70% efficacy (was rated "high" or "very high" efficacy). The sensitivity shows an approximately exponential decay. The features used in conjunctions are the most frequently occurring features included in DRM $RS_{0.951}$ (Table 2). The rule with l=1 consists of the most frequently occurring feature in $RS_{0.951}$ (F_{15}); the rule with l=2 consists of the two most frequently occurring features; and so on. In case of ties (e.g., both F_2 and F_5 occurred 6 times), the tied features are included in the rules separately, and the mean sensitivity and specificity of the resulting rules are used.

Supplementary Figure 6 Plot of sensitivity and specificity of rule sets (disjunctions of m rules) vs. m. A siRNA experiment was considered effective if it achieved >70% efficacy (was rated "high" or "very high" efficacy). The sensitivity rises in an approximately linear manner with increasing m. The 7 rules included in DRM $RS_{0.951}$ are used. For a given m, all possible rule sets, or disjunctions of m rules were constructed, and the average sensitivity and specificity of these rule sets (when applied to Set T) are shown. Error bars denote standard errors.

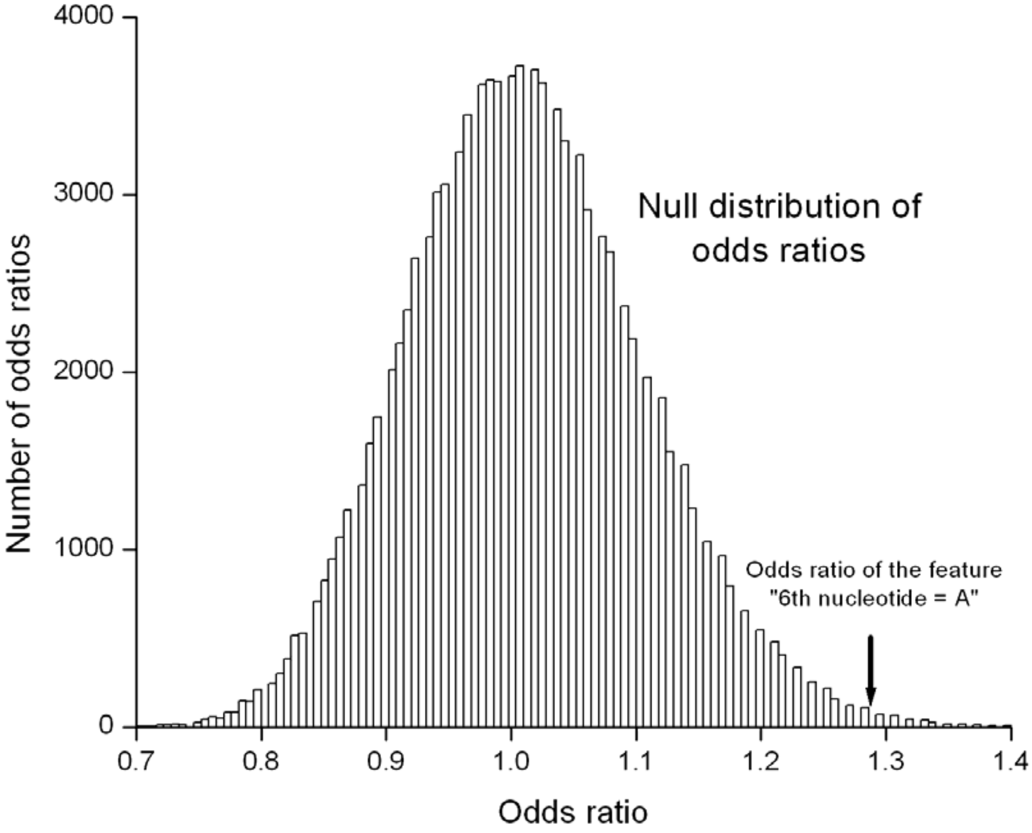

Supplementary Figure 1

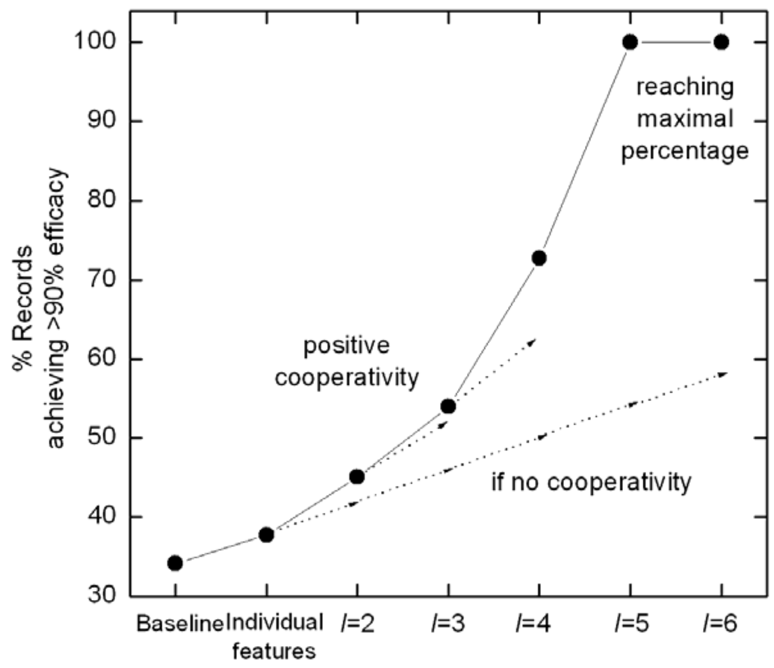

Supplementary Figure 2

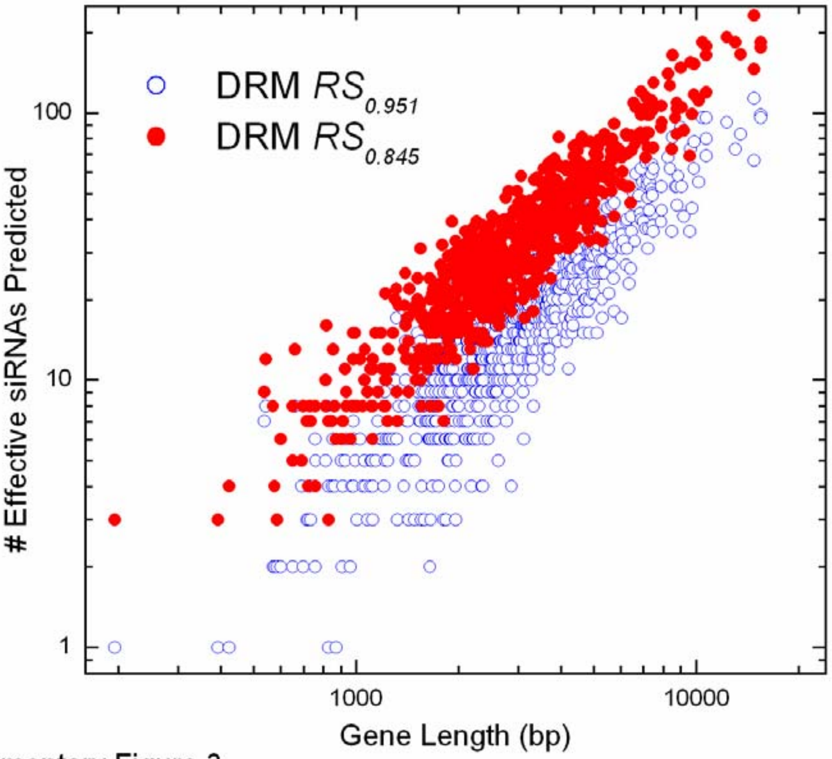

Supplementary Figure 3

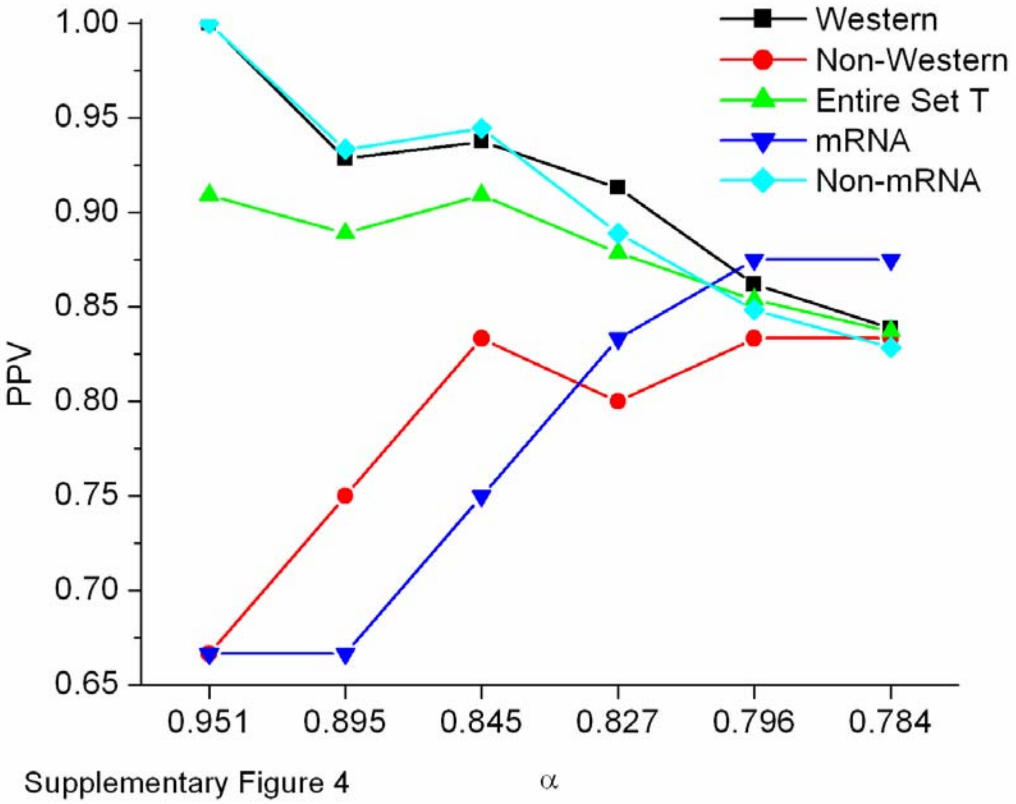

Supplementary Figure 4

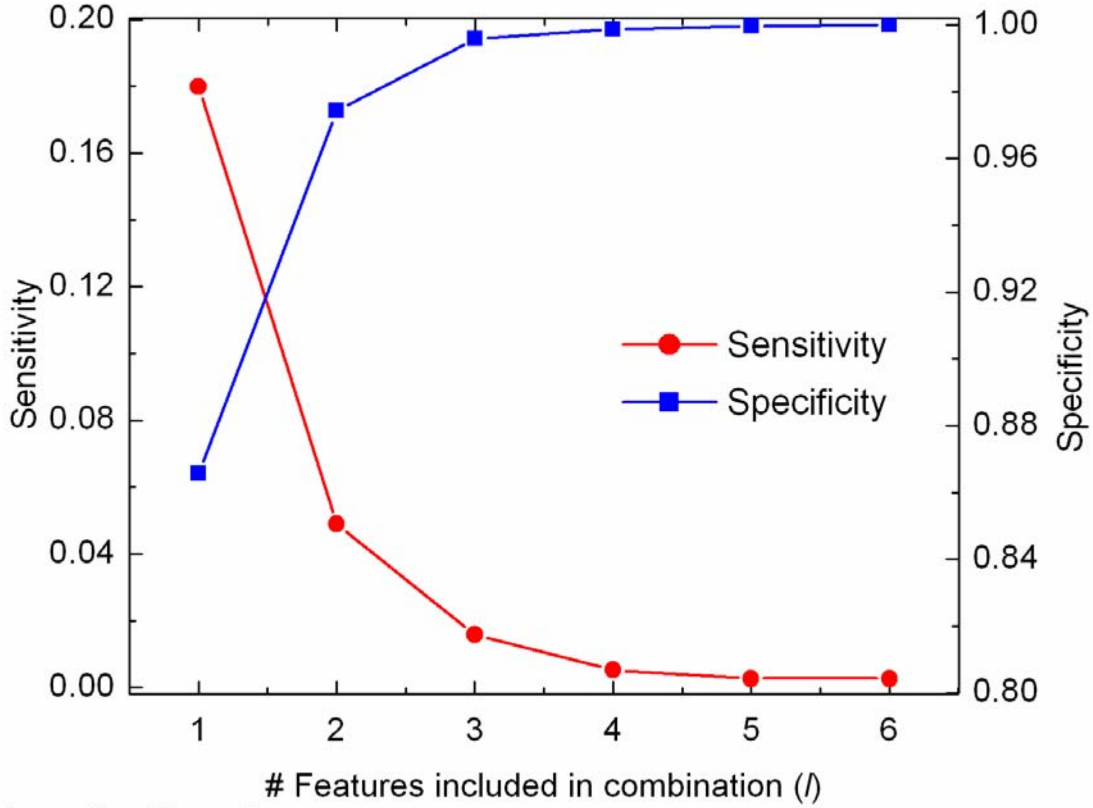

Supplementary Figure 5

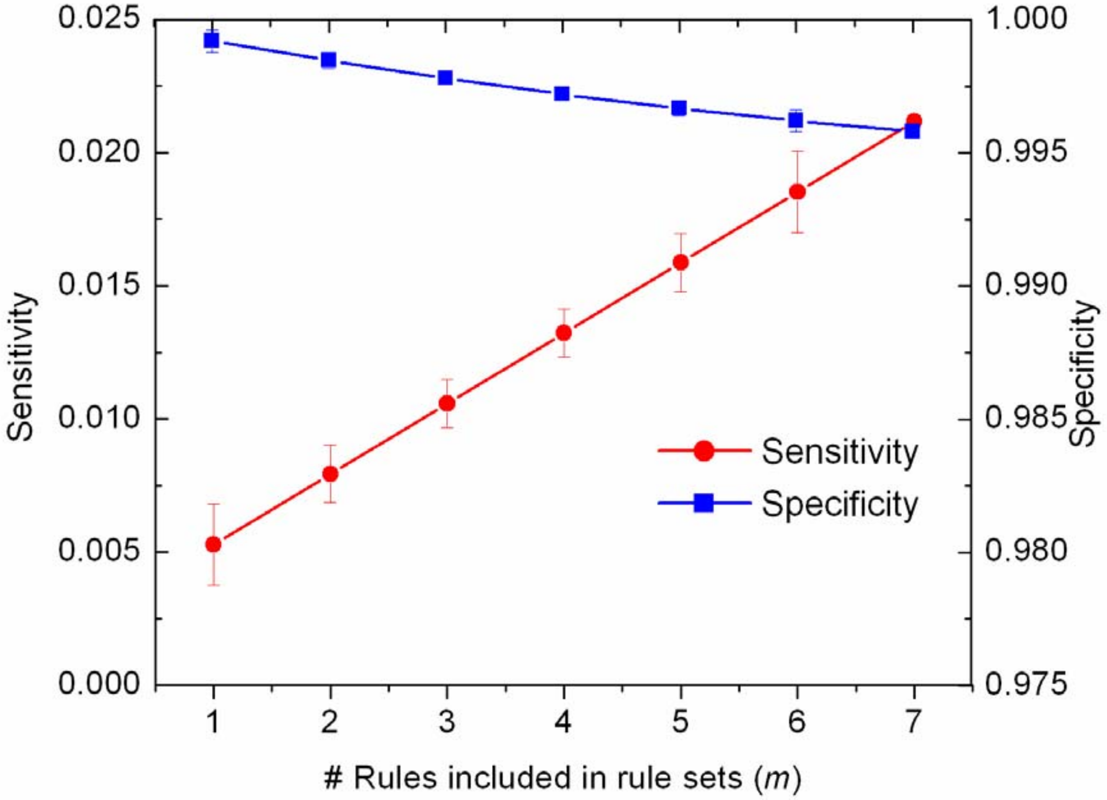

Supplementary Figure 6

Supplementary Table 1 Direct sequence features examined in this study and their significance in the survey.

Feature name	Ref	% Low	% Medium	% High	% Very high	# Low	# Medium	# High	# Very high	P_{70}	P_{90}	P_{wald}	Significance
1st nucleotide of the siRNA sequence=A		16.7	16.7	32.3	34.3	50	50	97	103	0.88	0.46	0.68	
1st nucleotide of the siRNA sequence≠A	[2]	14.6	16.3	35.0	34.1	276	307	659	642	0.12	0.54	0.32	
1st nucleotide of the siRNA sequence=U		21.3	15.2	30.1	33.4	63	45	89	99	1	0.64	0.95	
1st nucleotide of the siRNA sequence≠U	[2, 3]	13.9	16.5	35.3	34.2	263	312	667	646	0.0014	0.36	0.0495	**
1st nucleotide of the siRNA sequence=C		15.2	17.7	33.5	33.5	74	86	163	163	0.84	0.64	0.73	
1st nucleotide of the siRNA sequence≠C		14.8	16.0	34.9	34.3	252	271	593	582	0.16	0.36	0.27	
1st nucleotide of the siRNA sequence=G	[2]	12.6	16.0	36.9	34.5	139	176	407	380	0.0024	0.36	0.022	**
1st nucleotide of the siRNA sequence≠G		17.3	16.7	32.3	33.7	187	181	349	365	1	0.64	0.98	
2nd nucleotide of the siRNA sequence=A		12.1	16.0	33.8	38.1	77	102	215	243	0.010	0.0026	0.0019	***
2nd nucleotide of the siRNA sequence≠A		16.1	16.5	35.0	32.4	249	255	541	502	0.99	1	1	
2nd nucleotide of the siRNA sequence=U		17.4	14.0	37.2	31.4	60	48	128	108	0.54	0.95	0.84	
2nd nucleotide of the siRNA sequence≠U		14.5	16.8	34.1	34.6	266	309	628	637	0.46	0.052	0.16	
2nd nucleotide of the siRNA sequence=C		15.5	17.3	34.8	32.4	84	94	189	176	0.84	0.86	0.85	
2nd nucleotide of the siRNA sequence≠C		14.7	16.0	34.6	34.7	242	263	567	569	0.16	0.14	0.15	

2nd nucleotide of the siRNA sequence=G		15.9	17.1	33.9	33.0	105	113	224	218	0.90	0.77	0.86	
2nd nucleotide of the siRNA sequence≠G		14.5	16.0	34.9	34.6	221	244	532	527	0.098	0.23	0.14	
3rd nucleotide of the siRNA sequence=A	[4]	13.5	17.2	34.0	35.3	94	120	237	246	0.32	0.21	0.18	
3rd nucleotide of the siRNA sequence≠A		15.6	15.9	34.9	33.6	232	237	519	499	0.68	0.79	0.82	
3rd nucleotide of the siRNA sequence=U		14.8	15.2	34.0	36.0	72	74	165	175	0.20	0.12	0.19	
3rd nucleotide of the siRNA sequence≠U		15.0	16.7	34.8	33.6	254	283	591	570	0.80	0.88	0.81	
3rd nucleotide of the siRNA sequence=C		15.5	17.4	33.3	33.9	75	84	161	164	0.84	0.57	0.70	
3rd nucleotide of the siRNA sequence≠C		14.8	16.1	35.0	34.2	251	273	595	581	0.16	0.43	0.30	
3rd nucleotide of the siRNA sequence=G		16.4	15.3	37.3	30.9	85	79	193	160	0.61	0.98	0.92	
3rd nucleotide of the siRNA sequence≠G		14.5	16.7	33.8	35.1	241	278	563	585	0.39	0.018	0.085	*
4th nucleotide of the siRNA sequence=A		16.4	16.2	35.6	31.7	83	82	180	160	0.82	0.94	0.91	
4th nucleotide of the siRNA sequence≠A		14.5	16.4	34.3	34.8	243	275	576	585	0.18	0.062	0.091	
4th nucleotide of the siRNA sequence=U		14.7	16.6	37.3	31.4	70	79	178	150	0.50	0.96	0.79	
4th nucleotide of the siRNA sequence≠U		15.0	16.3	33.9	34.9	256	278	578	595	0.50	0.043	0.21	*
4th nucleotide of the siRNA sequence=C		14.1	15.4	31.5	39.0	88	96	197	244	0.098	0.00036	0.0075	***
4th nucleotide of the siRNA sequence≠C		15.3	16.7	35.9	32.1	238	261	559	501	0.90	1	0.99	
4th nucleotide of the siRNA		14.7	17.3	34.8	33.1	85	100	201	191	0.71	0.77	0.70	

sequence=G													
4th nucleotide of the siRNA sequence≠G		15.0	16.0	34.5	34.5	241	257	555	554	0.29	0.23	0.30	
5th nucleotide of the siRNA sequence=A		12.9	15.1	34.9	37.1	77	90	209	222	0.008	0.023	0.010	**
5th nucleotide of the siRNA sequence≠A		15.7	16.8	34.5	33.0	249	267	547	523	0.99	0.98	0.99	
5th nucleotide of the siRNA sequence=U		17.7	16.6	33.1	32.6	84	79	157	155	0.97	0.82	0.94	
5th nucleotide of the siRNA sequence≠U		14.2	16.3	35.0	34.5	242	278	599	590	0.026	0.18	0.064	*
5th nucleotide of the siRNA sequence=C		14.2	15.2	35.4	35.2	75	80	187	186	0.098	0.23	0.17	
5th nucleotide of the siRNA sequence≠C		15.2	16.7	34.4	33.8	251	277	569	559	0.90	0.77	0.83	
5th nucleotide of the siRNA sequence=G		15.4	18.5	34.8	31.2	90	108	203	182	0.97	0.98	0.97	
5th nucleotide of the siRNA sequence≠G		14.7	15.6	34.5	35.2	236	249	553	563	0.032	0.023	0.035	**
6th nucleotide of the siRNA sequence=A	[2, 3]	12.2	15.4	35.8	36.6	81	102	238	243	0.0024	0.043	0.0058	***
6th nucleotide of the siRNA sequence≠A		16.1	16.8	34.1	33.0	245	255	518	502	1	0.96	0.99	
6th nucleotide of the siRNA sequence=U		13.1	16.3	33.7	36.9	76	94	195	213	0.098	0.035	0.042	**
6th nucleotide of the siRNA sequence≠U		15.6	16.4	34.9	33.1	250	263	561	532	0.90	0.97	0.96	
6th nucleotide of the siRNA sequence=C		17.3	19.0	33.3	30.4	79	87	152	139	1	0.99	0.99	
6th nucleotide of the siRNA sequence≠C	[5]	14.3	15.6	35.0	35.1	247	270	604	606	0.00066	0.0089	0.0052	***
6th nucleotide of the siRNA sequence=G		18.6	15.3	35.3	30.9	90	74	171	150	0.95	0.98	0.98	

6th nucleotide of the siRNA sequence≠G		13.9	16.7	34.4	35.0	236	283	585	595	0.048	0.018	0.018	**
7th nucleotide of the siRNA sequence=A		14.2	15.4	31.8	38.7	82	89	184	224	0.12	0.0014	0.016	**
7th nucleotide of the siRNA sequence≠A		15.2	16.7	35.6	32.5	244	268	572	521	0.88	1	0.98	
7th nucleotide of the siRNA sequence=U		16.9	18.2	35.0	29.9	76	82	158	135	0.99	1	0.99	
7th nucleotide of the siRNA sequence≠U	[2]	14.4	15.9	34.5	35.2	250	275	598	610	0.010	0.0043	0.0091	***
7th nucleotide of the siRNA sequence=C		15.2	17.3	35.7	31.9	80	91	188	168	0.77	0.93	0.86	
7th nucleotide of the siRNA sequence≠C		14.8	16.1	34.3	34.8	246	266	568	577	0.23	0.074	0.14	
7th nucleotide of the siRNA sequence=G	[2]	14.0	15.2	36.0	34.8	88	95	226	218	0.069	0.32	0.17	
7th nucleotide of the siRNA sequence≠G		15.3	16.8	34.0	33.8	238	262	530	527	0.93	0.67	0.83	
8th nucleotide of the siRNA sequence=A	[2]	11.5	18.0	37.4	33.0	66	103	214	189	0.12	0.77	0.27	
8th nucleotide of the siRNA sequence≠A		16.1	15.8	33.6	34.5	260	254	542	556	0.88	0.23	0.73	
8th nucleotide of the siRNA sequence=U		16.5	16.1	33.5	33.9	78	76	158	160	0.80	0.54	0.72	
8th nucleotide of the siRNA sequence≠U		14.5	16.4	34.9	34.2	248	281	598	585	0.20	0.46	0.28	
8th nucleotide of the siRNA sequence=C		16.5	15.0	37.5	31.1	87	79	198	164	0.54	0.98	0.90	
8th nucleotide of the siRNA sequence≠C		14.4	16.8	33.7	35.1	239	278	558	581	0.46	0.023	0.098	*
8th nucleotide of the siRNA sequence=G		15.5	16.2	30.4	37.9	95	99	186	232	0.61	0.0054	0.13	*
8th nucleotide of the siRNA	[2]	14.7	16.4	36.3	32.6	231	258	570	513	0.39	0.99	0.87	

sequence≠G													
9th nucleotide of the siRNA sequence=A		17.3	17.6	32.9	32.2	106	108	202	198	0.99	0.90	0.98	
9th nucleotide of the siRNA sequence≠A		14.0	15.9	35.3	34.8	220	249	554	547	0.0061	0.10	0.021	**
9th nucleotide of the siRNA sequence=U	[2]	15.6	15.8	37.3	31.4	87	88	208	175	0.54	0.97	0.86	
9th nucleotide of the siRNA sequence≠U		14.7	16.5	33.7	35.1	239	269	548	570	0.46	0.035	0.14	*
9th nucleotide of the siRNA sequence=C		11.1	16.6	32.6	39.6	59	88	173	210	0.008	0.00021	0.00053	***
9th nucleotide of the siRNA sequence≠C		16.1	16.3	35.2	32.3	267	269	583	535	0.99	1	1	
9th nucleotide of the siRNA sequence=G		15.4	15.1	35.9	33.6	74	73	173	162	0.32	0.64	0.52	
9th nucleotide of the siRNA sequence≠G	[2]	14.8	16.7	34.3	34.3	252	284	583	583	0.68	0.36	0.48	
10th nucleotide of the siRNA sequence=A		15.2	17.7	32.2	34.9	73	85	155	168	0.84	0.29	0.56	
10th nucleotide of the siRNA sequence≠A		14.9	16.0	35.3	33.9	253	272	601	577	0.16	0.71	0.44	
10th nucleotide of the siRNA sequence=U	[4]	14.9	15.2	33.5	36.4	87	89	196	213	0.20	0.062	0.13	
10th nucleotide of the siRNA sequence≠U	[3]	14.9	16.8	35.0	33.3	239	268	560	532	0.80	0.94	0.87	
10th nucleotide of the siRNA sequence=C		13.4	16.4	37.3	32.9	76	93	212	187	0.16	0.79	0.41	
10th nucleotide of the siRNA sequence≠C		15.5	16.3	33.7	34.5	250	264	544	558	0.84	0.21	0.59	
10th nucleotide of the siRNA sequence=G		16.4	16.4	35.1	32.2	90	90	193	177	0.84	0.90	0.89	
10th nucleotide of the siRNA sequence≠G		14.4	16.3	34.5	34.8	236	267	563	568	0.16	0.10	0.11	

11th nucleotide of the siRNA sequence=A		14.4	15.2	34.6	35.8	81	86	195	202	0.13	0.14	0.14	
11th nucleotide of the siRNA sequence≠A	[5]	15.1	16.7	34.6	33.5	245	271	561	543	0.86	0.86	0.86	
11th nucleotide of the siRNA sequence=U		12.2	19.2	33.9	34.7	58	91	161	165	0.54	0.36	0.29	
11th nucleotide of the siRNA sequence≠U		15.7	15.6	34.8	33.9	268	266	595	580	0.46	0.64	0.71	
11th nucleotide of the siRNA sequence=C	[5]	16.3	16.3	36.3	31.2	86	86	192	165	0.80	0.97	0.93	
11th nucleotide of the siRNA sequence≠C		14.5	16.4	34.1	35.0	240	271	564	580	0.20	0.029	0.07	*
11th nucleotide of the siRNA sequence=G		16.4	15.3	33.8	34.6	101	94	208	213	0.61	0.36	0.57	
11th nucleotide of the siRNA sequence≠G		14.3	16.8	34.9	33.9	225	263	548	532	0.39	0.64	0.43	
12th nucleotide of the siRNA sequence=A		13.1	17.8	35.7	33.3	84	114	228	213	0.43	0.71	0.46	
12th nucleotide of the siRNA sequence≠A		15.7	15.7	34.2	34.4	242	243	528	532	0.57	0.29	0.54	
12th nucleotide of the siRNA sequence=U		16.3	16.7	34.9	32.1	94	96	201	185	0.88	0.91	0.91	
12th nucleotide of the siRNA sequence≠U		14.4	16.2	34.5	34.8	232	261	555	560	0.12	0.088	0.086	
12th nucleotide of the siRNA sequence=C		17.0	15.8	32.7	34.5	87	81	168	177	0.82	0.39	0.70	
12th nucleotide of the siRNA sequence≠C		14.3	16.5	35.2	34.0	239	276	588	568	0.18	0.61	0.30	
12th nucleotide of the siRNA sequence=G		13.4	14.5	34.9	37.3	61	66	159	170	0.013	0.023	0.027	**
12th nucleotide of the siRNA sequence≠G		15.3	16.8	34.5	33.3	265	291	597	575	0.99	0.98	0.97	
13th nucleotide of the siRNA	[5]	13.1	14.8	35.6	36.5	80	90	217	222	0.008	0.052	0.020	**

sequence=A													
13th nucleotide of the siRNA sequence≠A		15.6	17.0	34.2	33.2	246	267	539	523	0.99	0.95	0.98	
13th nucleotide of the siRNA sequence=U	[3]	14.9	18.0	33.5	33.5	71	86	160	160	0.84	0.64	0.71	
13th nucleotide of the siRNA sequence≠U		14.9	15.9	34.9	34.3	255	271	596	585	0.16	0.36	0.29	
13th nucleotide of the siRNA sequence=C		15.7	17.5	34.0	32.8	93	104	202	195	0.90	0.82	0.86	
13th nucleotide of the siRNA sequence≠C		14.7	15.9	34.8	34.6	233	253	554	550	0.098	0.18	0.14	
13th nucleotide of the siRNA sequence=G		16.3	15.3	35.1	33.3	82	77	177	168	0.57	0.71	0.69	
13th nucleotide of the siRNA sequence≠G	[4]	14.5	16.7	34.5	34.3	244	280	579	577	0.43	0.29	0.31	
14th nucleotide of the siRNA sequence=A		13.2	16.3	35.9	34.6	81	100	220	212	0.12	0.36	0.17	
14th nucleotide of the siRNA sequence≠A		15.6	16.4	34.1	33.9	245	257	536	533	0.88	0.64	0.83	
14th nucleotide of the siRNA sequence=U		14.6	16.3	32.0	37.1	69	77	151	175	0.43	0.035	0.16	*
14th nucleotide of the siRNA sequence≠U		15.0	16.4	35.3	33.3	257	280	605	570	0.57	0.97	0.84	
14th nucleotide of the siRNA sequence=C		15.6	17.2	38.0	29.2	80	88	195	150	0.82	1	0.98	
14th nucleotide of the siRNA sequence≠C		14.7	16.1	33.6	35.6	246	269	561	595	0.18	0.00053	0.019	**
14th nucleotide of the siRNA sequence=G		16.4	15.7	32.4	35.5	96	92	190	208	0.71	0.18	0.48	
14th nucleotide of the siRNA sequence≠G		14.4	16.6	35.4	33.6	230	265	566	537	0.29	0.82	0.52	
15th nucleotide of the siRNA sequence=A		14.8	16.9	35.0	33.2	98	112	232	220	0.64	0.74	0.67	

15th nucleotide of the siRNA sequence≠A		15.0	16.1	34.4	34.5	228	245	524	525	0.36	0.26	0.33	
15th nucleotide of the siRNA sequence=U	[2]	12.6	17.6	33.8	36.0	70	98	188	200	0.23	0.10	0.10	
15th nucleotide of the siRNA sequence≠U		15.7	15.9	34.9	33.5	256	259	568	545	0.77	0.90	0.90	
15th nucleotide of the siRNA sequence=C		16.9	18.4	33.1	31.6	83	90	162	155	1	0.95	0.98	
15th nucleotide of the siRNA sequence≠C		14.3	15.8	35.1	34.8	243	267	594	590	0.0045	0.052	0.023	**
15th nucleotide of the siRNA sequence=G		15.8	12.0	36.6	35.7	75	57	174	170	0.010	0.16	0.12	*
15th nucleotide of the siRNA sequence≠G		14.7	17.6	34.1	33.7	251	300	582	575	0.99	0.84	0.88	
16th nucleotide of the siRNA sequence=A		14.5	16.5	35.5	33.5	80	91	196	185	0.43	0.64	0.52	
16th nucleotide of the siRNA sequence≠A		15.1	16.3	34.3	34.3	246	266	560	560	0.57	0.36	0.48	
16th nucleotide of the siRNA sequence=U		16.9	16.9	32.7	33.5	84	84	162	166	0.95	0.67	0.86	
16th nucleotide of the siRNA sequence≠U		14.3	16.2	35.2	34.3	242	273	594	579	0.048	0.32	0.14	*
16th nucleotide of the siRNA sequence=C	[3]	13.8	14.7	35.6	35.9	81	86	209	211	0.026	0.10	0.058	*
16th nucleotide of the siRNA sequence≠C		15.3	17.0	34.3	33.4	245	271	547	534	0.97	0.90	0.94	
16th nucleotide of the siRNA sequence=G	[5]	14.8	17.5	34.4	33.3	81	96	189	183	0.74	0.71	0.68	
16th nucleotide of the siRNA sequence≠G		15.0	16.0	34.7	34.4	245	261	567	562	0.26	0.29	0.32	
17th nucleotide of the siRNA sequence=A		11.4	15.5	37.1	35.9	75	102	244	236	0.00049	0.10	0.0049	***
17th nucleotide of the siRNA		16.4	16.7	33.5	33.3	251	255	512	509	1	0.90	1	

sequence≠A												
17th nucleotide of the siRNA sequence=U		14.8	16.3	33.1	35.9	79	87	177	192	0.43	0.12	0.25	
17th nucleotide of the siRNA sequence≠U		15.0	16.4	35.1	33.5	247	270	579	553	0.57	0.88	0.75	
17th nucleotide of the siRNA sequence=C		16.9	15.9	36.6	30.7	83	78	180	151	0.82	0.99	0.96	
17th nucleotide of the siRNA sequence≠C		14.4	16.5	34.0	35.1	243	279	576	594	0.18	0.012	0.044	**
17th nucleotide of the siRNA sequence=G		17.8	18.0	31.0	33.2	89	90	155	166	1	0.71	0.96	
17th nucleotide of the siRNA sequence≠G		14.1	15.9	35.7	34.4	237	267	601	579	0.0018	0.29	0.041	**
18th nucleotide of the siRNA sequence=A		13.5	15.6	34.7	36.1	96	111	247	257	0.048	0.074	0.041	**
18th nucleotide of the siRNA sequence≠A		15.6	16.7	34.6	33.1	230	246	509	488	0.95	0.93	0.96	
18th nucleotide of the siRNA sequence=U		14.3	16.2	33.4	36.0	84	95	196	211	0.32	0.10	0.17	
18th nucleotide of the siRNA sequence≠U		15.1	16.4	35.0	33.4	242	262	560	534	0.68	0.90	0.83	
18th nucleotide of the siRNA sequence=C		17.0	18.1	35.8	29.1	74	79	156	127	0.99	1	1	
18th nucleotide of the siRNA sequence≠C		14.4	15.9	34.3	35.4	252	278	600	618	0.010	0.00071	0.0048	***
18th nucleotide of the siRNA sequence=G		16.0	16.0	34.8	33.3	72	72	157	150	0.64	0.71	0.70	
18th nucleotide of the siRNA sequence≠G		14.7	16.4	34.6	34.3	254	285	599	595	0.36	0.29	0.30	
19th nucleotide of the siRNA sequence=A		12.7	14.2	35.3	37.8	80	90	223	239	0.00066	0.0054	0.0014	***
19th nucleotide of the siRNA sequence≠A		15.9	17.2	34.3	32.6	246	267	533	506	1	0.99	1	

19th nucleotide of the siRNA sequence=U	[5]	11.1	18.2	35.4	35.2	55	90	175	174	0.098	0.23	0.086	
19th nucleotide of the siRNA sequence≠U		16.0	15.8	34.4	33.8	271	267	581	571	0.90	0.77	0.91	
19th nucleotide of the siRNA sequence=C		17.2	16.2	34.1	32.5	96	90	190	181	0.92	0.86	0.92	
19th nucleotide of the siRNA sequence≠C		14.1	16.4	34.8	34.7	230	267	566	564	0.082	0.14	0.075	
19th nucleotide of the siRNA sequence=G		19.0	17.4	33.5	30.1	95	87	168	151	1	1	1	
19th nucleotide of the siRNA sequence≠G	[2, 3, 5]	13.7	16.0	34.9	35.3	231	270	588	594	0.00049	0.0043	0.0012	***

Features marked by *: at least one of the three p-values is less than 0.05.
Features marked by **: at least two of the three p-values are less than 0.05.
Features marked by ***: (P_{wald} <0.01) and (P_{70}<0.01 or P_{90}<0.01).
At P<0.05, the FDR is controlled at 0.18, 0.15 and 0.16 for the Wald test of monotone trend, the odds ratio permutation test (>70%), and the odds ratio permutation test (>90%), respectively. At P<0.01, the FDR is controlled at 0.056, 0.044 and 0.038 for the three tests, respectively.

Supplementary Table 2 Sequence-derived features examined in this study and their significance in the survey

**Feature name**	**Ref**	**% Low**	**% Medium**	**% High**	**% Very high**	**# Low**	**# Medium**	**# High**	**# Very high**	P_{70}	P_{90}	P_{wald}	**Significance**
1st nucleotide of the siRNA sequence=(G/C)	[3, 6, 7]	13.4	16.5	35.9	34.2	213	262	570	543	0.0061	0.43	0.049	**
1st nucleotide of the siRNA sequence≠(G/C)		19.0	15.9	31.2	33.9	113	95	186	202	0.99	0.57	0.95	
10th nucleotide of the siRNA sequence=(A/U)	[6]	15.0	16.3	32.9	35.7	160	174	351	381	0.54	0.062	0.19	
10th nucleotide of the siRNA sequence≠(A/U)		14.8	16.4	36.2	32.6	166	183	405	364	0.46	0.94	0.81	
11th nucleotide of the siRNA	[5]	16.3	15.7	34.9	33.0	187	180	400	378	0.80	0.88	0.92	

sequence=(G/C)													
11th nucleotide of the siRNA sequence≠(G/C)		13.4	17.0	34.3	35.3	139	177	356	367	0.20	0.12	0.078	
19th nucleotide of the siRNA sequence=(A/U)	[3, 6, 7]	12.0	16.0	35.3	36.7	135	180	398	413	0.00029	0.0043	0.000058	***
19th nucleotide of the siRNA sequence≠(A/U)		18.1	16.7	33.8	31.4	191	177	358	332	1	1	1	
There are at least five (A/U)s in the seven nucleotides at the 3' end	[7]	13.1	15.4	36.5	35.0	70	82	195	187	0.026	0.26	0.10	*
There are four or fewer (A/U)s in the seven nucleotides at the 3' end		15.5	16.7	34.0	33.8	256	275	561	558	0.97	0.74	0.90	
There are at least three (A/U)s in the seven nucleotides at the 3' end	[6]	13.4	16.4	33.7	36.5	246	300	618	668	0.00001	0.00001	2.5e-09	***
There are two or fewer (A/U)s in the seven nucleotides at the 3' end		22.7	16.2	39.2	21.9	80	57	138	77	1	1	1	
There are occurrences of three or more identical nucleotides in a row		16.5	17.4	33.8	32.4	173	183	355	340	0.99	0.96	0.99	
There are no occurrences of three or more identical nucleotides in a row	[9, 13]	13.5	15.4	35.4	35.7	153	174	401	405	0.0061	0.043	0.0067	***
There are occurrences of four or more identical nucleotides in a row		22.5	21.0	26.5	30.0	45	42	53	60	1	0.99	1	
There are no occurrences of four or more identical nucleotides in a row	[9-12]	14.2	15.9	35.4	34.5	281	315	703	685	0.00001	0.012	0.0014	***
There are at least three (A/U)s in the five nucleotides at the 5'	[4]	16.4	15.4	34.9	33.3	131	123	279	266	0.64	0.74	0.80	

end													
There are two or fewer (A/U)s in the five nucleotides at the 5' end		14.1	16.9	34.4	34.6	195	234	477	479	0.36	0.26	0.20	
There are at least three (A/U)s in the five nucleotides at the 3' end	[4]	12.6	16.4	34.0	37.0	164	214	443	483	0.0018	0.00016	0.000022	***
There are two or fewer (A/U)s in the five nucleotides at the 3' end		18.4	16.3	35.6	29.8	162	143	313	262	1	1	1	
There are occurrences of G/C stretches of length 7 or longer	[9, 10]	42.6	14.9	23.4	19.1	20	7	11	9	1	1	1	
There are no occurrences of G/C stretches of length 7 or longer		14.3	16.4	34.9	34.4	306	350	745	736	0.00001	0.00001	0.000015	***
G/C content is between 32 and 79%	[14]	14.8	16.3	35.0	33.9	310	340	730	708	0.058	0.99	0.56	
G/C content is not between 32 and 79%		16.7	17.7	27.1	38.5	16	17	26	37	0.94	0.012	0.44	*
G/C content is between 30 and 70%	[15]	14.5	16.2	34.9	34.4	307	344	739	728	0.00001	0.00001	0.0028	***
G/C content is not between 30 and 70%		28.8	19.7	25.8	25.8	19	13	17	17	1	1	1	
G/C content is between 30 and 52%	[4]	13.8	16.1	34.7	35.4	146	171	368	375	0.082	0.12	0.056	
G/C content is not between 30 and 52%		16.0	16.5	34.5	32.9	180	186	388	370	0.92	0.88	0.94	
G/C content is between 35 and 60%	[9]	13.3	16.7	35.1	35.0	245	308	649	647	0.00001	0.0019	0.00018	***
G/C content is not between 35 and 60%		24.2	14.6	31.9	29.3	81	49	107	98	1	1	1	
G/C content is between 20 and 50%	[13]	13.8	16.2	34.4	35.7	148	174	370	384	0.082	0.062	0.037	*
G/C content is not between 20		16.1	16.5	34.8	32.6	178	183	386	361	0.92	0.94	0.96	

and 50%													
G/C content is between 31.6 and 57.9%	[3]	13.3	16.7	35.1	35.0	245	308	649	647	0.00001	0.0019	0.00018	***
G/C content is not between 31.6 and 57.9%		24.2	14.6	31.9	29.3	81	49	107	98	1	1	1	

Features marked by *: at least one of the three p-values is less than 0.05.
Features marked by **: at least two of the three p-values are less than 0.05.
Features marked by ***: (P_{wald} <0.01) and (P_{70}<0.01 or P_{90}<0.01).
At P<0.05, the FDR is controlled at 0.18, 0.15 and 0.16 for the Wald test of monotone trend, the odds ratio permutation test (>70%), and the odds ratio permutation test (>90%), respectively. At P<0.01, the FDR is controlled at 0.056, 0.044 and 0.038 for the three tests, respectively.

Supplementary Table 3 Thermodynamic features examined in this study and their significance in the survey

Feature name	Ref	% Low	% Medium	% High	% Very high	# Low	# Medium	# High	# Very high	P_{70}	P_{90}	P_{wald}	Significance
$T_m \geq 60°C$		19.5	16.2	32.3	31.9	107	89	177	175	1	0.93	0.99	
$T_m < 60°C$	[4]	13.4	16.4	35.4	34.8	219	268	579	570	0.0014	0.074	0.0057	***
T_m is between 20 and 60°C		13.2	16.5	35.0	35.3	200	249	529	534	0.0045	0.023	0.003	***
T_m is not between 20 and 60°C		18.8	16.1	33.8	31.4	126	108	227	211	1	0.98	1	
$T_m < 20°C$	[4]	15.3	15.3	40.3	29.0	19	19	50	36	0.36	1	0.76	
$T_m \geq 20°C$		14.9	16.4	34.3	34.4	307	338	706	709	0.64	0.0026	0.24	*
Binding energy of N16-N19 > -9 KCal/Mol	[16]	11.8	17.1	34.0	37.1	124	179	357	389	0.010	0.0026	0.00025	***
Binding energy of N16-N19 ≤ -9 KCal/Mol		17.8	15.7	35.2	31.4	202	178	399	356	0.99	1	1	
Binding energy of N6-N11 ≥ -13 KCal/Mol		14.1	17.3	34.3	34.4	157	192	381	382	0.54	0.39	0.38	
Binding energy of N6-N11 < -13 KCal/Mol	[17]	15.8	15.4	35.0	33.9	169	165	375	363	0.46	0.61	0.62	
Binding energy of N7-N12 > -13 KCal/Mol		15.3	17.9	34.0	32.8	158	185	352	339	0.97	0.90	0.95	

Binding energy of N7-N12 ≤ -13 KCal/Mol	[16]	14.6	15.0	35.1	35.3	168	172	404	406	0.032	0.10	0.054	*
Mean of free energy profile of N7-N11 is between -1.97 and -1.65 KCal/Mol (inclusive)	[18]	12.7	18.5	34.4	34.4	66	96	179	179	0.46	0.43	0.32	
Mean of free energy profile of N7-N11 is not between -1.97 and -1.65 KCal/Mol (inclusive)		15.6	15.7	34.7	34.0	260	261	577	566	0.54	0.57	0.68	
Binding energy of N1-N4 is between -9 and -5 KCal/Mol (exclusive)	[16]	16.4	15.7	35.6	32.3	99	95	215	195	0.71	0.90	0.87	
Binding energy of N1-N4 is not between -9 and -5 KCal/Mol (exclusive)		14.4	16.6	34.2	34.8	227	262	541	550	0.29	0.10	0.13	
Binding energy of N16-N19 ≥ binding energy of N1-N4	[16, 17]	13.4	16.3	34.7	35.6	191	233	495	507	0.013	0.018	0.0043	**
Binding energy of N16-N19 < binding energy of N1-N4		17.8	16.4	34.4	31.4	135	124	261	238	0.99	0.98	1	
Binding energy of N16-N19 – binding energy of N1-N4 is between 0 and 1 KCal/Mol	[16]	12.6	14.9	32.5	39.9	44	52	113	139	0.010	0.00036	0.0078	***
Binding energy of N16-N19 – binding energy of N1-N4 is not between 0 and 1 KCal/Mol		15.4	16.6	35.0	33.0	282	305	643	606	0.99	1	0.99	
Binding energy of N15-N19 > binding energy of N1-N5	[18]	13.5	17.1	34.8	34.6	181	229	465	463	0.20	0.26	0.12	
Binding energy of N15-N19 ≤ binding energy of N1-N5		17.1	15.1	34.4	33.3	145	128	291	282	0.80	0.74	0.88	
Absolute value of total hairpin energy < 1 KCal/Mol	[16]	12.7	17.0	35.0	35.3	48	64	132	133	0.18	0.23	0.19	
Absolute value of total hairpin energy ≥ 1 KCal/Mol		15.4	16.2	34.5	33.9	278	293	624	612	0.82	0.77	0.81	
Folding energy of sense strand ≥	[9]	14.6	16.4	34.6	34.4	307	345	729	724	0.00004	0.00002	0.023	**

-5 KCal/Mol													
Folding energy of sense strand < -5 KCal/Mol		24.1	15.2	34.2	26.6	19	12	27	21	1	1	0.98	

Features marked by *: at least one of the three p-values is less than 0.05.
Features marked by **: at least two of the three p-values are less than 0.05.
Features marked by ***: (P_{wald} <0.01) and (P_{70}<0.01 or P_{90}<0.01).
At P<0.05, the FDR is controlled at 0.18, 0.15 and 0.16 for the Wald test of monotone trend, the odds ratio permutation test (>70%), and the odds ratio permutation test (>90%), respectively. At P<0.01, the FDR is controlled at 0.056, 0.044 and 0.038 for the three tests, respectively.

Supplementary Table 4 Features defined based on target mRNA sites examined in this study and their significance in the survey

**Feature name**	**Ref**	**% Low**	**% Medium**	**% High**	**% Very high**	**# Low**	**# Medium**	**# High**	**# Very high**	P_{70}	P_{90}	P_{wald}	**Significance**
Local folding potential (mean) ≥ -22.72 KCal/Mol	[31]	12.0	14.6	34.7	38.7	132	161	382	426	0.00001	0.00001	9.3e-09	***
Local folding potential (mean) < -22.72 KCal/Mol		17.9	18.1	34.5	29.5	194	196	374	319	1	1	1	
Anti-sense siRNA binding energy > -10 KCal/Mol	[12]	15.8	16.3	34.1	33.8	269	279	582	577	0.97	0.77	0.93	
Anti-sense siRNA binding energy ≤ -10 KCal/Mol		11.9	16.4	36.5	35.2	57	78	174	168	0.026	0.23	0.072	*
Accessibility score = 0		15.5	15.7	34.8	34.0	277	282	623	610	0.43	0.57	0.63	
Accessibility score > 0	[19]	12.5	19.1	33.9	34.4	49	75	133	135	0.57	0.43	0.37	
H-b index ≥ 28.8		17.6	15.5	34.0	32.9	197	174	381	369	0.97	0.88	0.99	
H-b index < 28.8	[22]	12.1	17.2	35.3	35.4	129	183	375	376	0.032	0.12	0.012	**
Does not pass Repelling Loop Filter		13.2	16.7	31.7	38.4	37	47	89	108	0.20	0.0089	0.087	*
Passes Repelling Loop Filter	[20]	15.2	16.3	35.0	33.5	289	310	667	637	0.80	0.99	0.91	
Local free energy of the most stable structure ≥ -20.9 KCal/Mol	[21]	14.2	15.9	34.1	35.8	156	174	373	392	0.13	0.052	0.048	*

Local free energy of the most stable structure < -20.9 KCal/Mol		15.6	16.8	35.2	32.4	170	183	383	353	0.86	0.95	0.95	
Average local free energy of the ten most stable structures ≥ -20.85 KCal/Mol	[21]	14.4	15.9	34.9	34.8	157	173	381	380	0.16	0.23	0.16	
Average local free energy of the ten most stable structures < -20.85 KCal/Mol		15.5	16.8	34.3	33.4	169	184	375	365	0.84	0.77	0.84	
Target site is on the 1st quartile of CDS		12.8	14.9	38.0	34.3	93	108	276	249	0.0024	0.43	0.056	*
Target site is not on the 1st quartile of CDS		16.0	17.1	32.9	34.0	233	249	480	496	1	0.57	0.94	
Target site is on the 2nd quartile of CDS		15.8	15.6	31.4	37.2	78	77	155	184	0.54	0.023	0.21	*
Target site is not on the 2nd quartile of CDS		14.7	16.6	35.6	33.2	248	280	601	561	0.46	0.98	0.79	
Target site is on the 3rd quartile of CDS		12.5	15.3	34.8	37.3	49	60	136	146	0.017	0.029	0.031	**
Target site is not on the 3rd quartile of CDS	[5]	15.4	16.6	34.6	33.4	277	297	620	599	0.98	0.97	0.97	
Target site is on the 4th quartile of CDS		18.7	19.1	30.4	31.8	53	54	86	90	1	0.91	0.98	
Target site is not on the 4th quartile of CDS		14.4	15.9	35.2	34.5	273	303	670	655	0.00015	0.088	0.025	**
Target site is on the 5'UTR		25.6	15.4	38.5	20.5	10	6	15	8	1	1	0.98	
Target site is not on the 5'UTR	[11, 14]	14.7	16.4	34.5	34.4	316	351	741	737	0.00001	0.00001	0.016	**
Target site is on the 3'UTR		20.6	19.1	38.2	22.1	27	25	50	29	1	1	1	
Target site is not on the 3'UTR	[11, 14]	14.6	16.2	34.4	34.9	299	332	706	716	0.00001	0.00001	0.00052	***
Target site is on the first 100 nucleotides of CDS		15.2	14.5	35.9	34.5	22	21	52	50	0.18	0.43	0.41	
Target site is not on the first 100 nucleotides of CDS	[11, 14]	14.9	16.5	34.5	34.1	304	336	704	695	0.82	0.57	0.59	

Target site is on CDS	[9]	14.4	16.2	34.3	35.2	288	324	688	705	0.00001	0.00001	0.000055	***
Target site is not on CDS		21.2	18.4	38.0	22.3	38	33	68	40	1	1	1	

Features marked by *: at least one of the three p-values is less than 0.05.
Features marked by **: at least two of the three p-values are less than 0.05.
Features marked by ***: (P_{wald} <0.01) and (P_{70}<0.01 or P_{90}<0.01).
At P<0.05, the FDR is controlled at 0.18, 0.15 and 0.16 for the Wald test of monotone trend, the odds ratio permutation test (>70%), and the odds ratio permutation test (>90%), respectively. At P<0.01, the FDR is controlled at 0.056, 0.044 and 0.038 for the three tests, respectively.

Supplementary Table 5 Features based on experimental settings examined in this study

Feature name	Ref	% Low	% Medium	% High	% Very high	# Low	# Medium	# High	# Very high	P_{70}	P_{90}	P_{wald}	Significance
Cell line = HeLa		7.9	10.6	41.2	40.3	33	44	172	168	0.00001	0.00016	4e-09	***
Cell line ≠ HeLa		16.6	17.7	33.1	32.7	293	313	584	577	1	1	1	
Cell line = HEK293		17.1	17.9	28.5	36.5	45	47	75	96	0.98	0.088	0.61	
Cell line ≠ HEK293		14.6	16.1	35.5	33.8	281	310	681	649	0.021	0.91	0.39	*
Cell line = MCF7		12.9	21.0	38.7	27.4	8	13	24	17	0.90	1	0.79	
Cell line ≠ MCF7		15.0	16.2	34.5	34.3	318	344	732	728	0.098	0.00016	0.21	*
Cell line = CV-1 and derivatives		35.2	1.9	37.0	25.9	19	1	20	14	1	1	0.97	
Cell line ≠ CV-1 and derivatives		14.4	16.7	34.6	34.3	307	356	736	731	0.0018	0.00001	0.027	**
Cell line = 3T3		16.7	18.5	40.7	24.1	9	10	22	13	0.97	1	0.92	
Cell line ≠ 3T3		14.9	16.3	34.5	34.4	317	347	734	732	0.026	0.00001	0.085	**
Test method = Western blot		10.8	15.5	34.8	38.9	138	198	445	498	0.00001	0.00001	3.8e-14	***
Test method ≠ Western blot		20.8	17.6	34.4	27.3	188	159	311	247	1	1	1	
Test method = PCR-related		17.7	21.8	34.2	26.2	100	123	193	148	1	1	1	
Test method ≠ PCR-related		14.0	14.4	34.8	36.9	226	234	563	597	0.00001	0.00001	2.6e-08	***
Test method = bDNA		22.2	11.1	26.7	40.0	10	5	12	18	0.84	0.0019	0.47	*

Test method ≠ bDNA		14.8	16.5	34.8	34.0	316	352	744	727	0.16	1	0.53	
Test method = Northern blot		24.0	15.6	34.4	26.0	23	15	33	25	1	1	0.99	
Test method ≠ Northern blot		14.5	16.4	34.6	34.5	303	342	723	720	0.00001	0.00001	0.010	**
Test method = Luciferase assay		44.7	2.1	44.7	8.5	21	1	21	4	1	1	1	
Test method ≠ Luciferase assay		14.3	16.7	34.4	34.7	305	356	735	741	0.00001	0.00001	4.7e-08	***
Transfection method = Synthesized oligos		14.1	16.0	34.9	35.0	213	241	527	529	0.026	0.074	0.028	**
Transfection method = Vector-based		16.8	17.2	34.0	32.0	113	116	229	216	0.97	0.93	0.97	
Test object = mRNA		18.6	20.0	34.2	27.1	136	146	250	198	1	1	1	
Test object ≠ mRNA		13.1	14.5	34.8	37.6	190	211	506	547	0.00001	0.00001	9.3e-10	***
Test object = protein		12.7	14.5	35.0	37.8	182	209	503	543	0.00001	0.00001	2.2e-09	***
Test object ≠ protein		19.3	19.8	33.9	27.0	144	148	253	202	1	1	1	

Features marked by *: at least one of the three p-values is less than 0.05.
Features marked by **: at least two of the three p-values are less than 0.05.
Features marked by ***: (P_{wald} <0.01) and (P_{70}<0.01 or P_{90}<0.01).
At P<0.05, the FDR is controlled at 0.18, 0.15 and 0.16 for the Wald test of monotone trend, the odds ratio permutation test (>70%), and the odds ratio permutation test (>90%), respectively. At P<0.01, the FDR is controlled at 0.056, 0.044 and 0.038 for the three tests, respectively.

Supplementary Table 6 Non-redundant rule sets for four α levels (denoted as RS_{α}) following the DRM procedure. Listing of F_1-F_{17} is at the end of the table.

$RS_{0.951}$

Feature	F_1	F_2	F_3	F_4	F_5	F_6	F_7	F_8	F_9	F_{10}	F_{11}	F_{12}	F_{13}	F_{14}	F_{15}	F_{16}	F_{17}
Rule 1	√	√			√										√		
Rule 2		√			√			√						√	√		
Rule 3	√	√				√									√	√	√
Rule 4		√			√		√	√							√	√	
Rule 5	√				√								√	√	√	√	
Rule 6		√			√	√	√	√							√		
Rule 7		√			√		√	√					√		√		

$RS_{0.895}$

Feature	F_1	F_2	F_3	F_4	F_5	F_6	F_7	F_8	F_9	F_{10}	F_{11}	F_{12}	F_{13}	F_{14}	F_{15}	F_{16}	F_{17}
Rule 1	√	√			√										√		
Rule 2		√			√			√						√	√		
Rule 3	√	√				√									√	√	√
Rule 4		√			√		√	√							√	√	
Rule 5	√				√								√	√	√	√	
Rule 6		√			√	√	√	√							√		
Rule 7		√			√		√	√					√		√		
Rule 8	√				√		√						√	√	√		
Rule 9	√				√	√							√	√		√	

$RS_{0.845}$

Feature	F_1	F_2	F_3	F_4	F_5	F_6	F_7	F_8	F_9	F_{10}	F_{11}	F_{12}	F_{13}	F_{14}	F_{15}	F_{16}	F_{17}
Rule 1	√	√			√										√		
Rule 2		√			√	√		√							√		
Rule 3		√			√			√						√	√		
Rule 4		√			√			√					√		√		
Rule 5	√				√							√		√	√	√	
Rule 6		√		√	√			√				√			√		
Rule 7		√			√		√	√	√						√		
Rule 8		√		√	√			√	√						√		
Rule 9	√	√				√									√	√	√
Rule 10		√			√		√	√							√	√	
Rule 11		√					√	√					√		√	√	
Rule 12	√	√		√									√		√	√	
Rule 13	√				√									√	√	√	√
Rule 14	√				√								√	√	√	√	
Rule 15		√		√	√		√	√							√		
Rule 16		√			√		√	√			√				√		
Rule 17	√			√	√		√						√		√		
Rule 18	√				√		√						√	√	√		

Rule 19		√		√	√			√			√				√		
Rule 20	√				√	√							√	√		√	

$RS_{0.827}$

**Feature**	**F_1**	**F_2**	**F_3**	**F_4**	**F_5**	**F_6**	**F_7**	**F_8**	**F_9**	**F_{10}**	**F_{11}**	**F_{12}**	**F_{13}**	**F_{14}**	**F_{15}**	**F_{16}**	**F_{17}**
Rule 1	√	√			√										√		
Rule 2		√			√	√		√							√		
Rule 3		√			√			√						√	√		
Rule 4		√			√			√					√		√		
Rule 5	√			√		√						√			√	√	
Rule 6	√				√							√		√	√	√	
Rule 7		√			√		√	√				√			√		
Rule 8		√		√	√			√				√			√		
Rule 9		√			√		√	√	√						√		
Rule 10		√		√	√			√	√						√		
Rule 11		√				√	√	√							√	√	
Rule 12	√	√				√									√	√	√
Rule 13	√			√		√							√		√	√	
Rule 14	√				√	√								√	√	√	
Rule 15	√					√							√	√	√	√	
Rule 16		√			√		√	√							√	√	
Rule 17		√					√	√					√		√	√	
Rule 18	√	√						√					√		√	√	
Rule 19	√	√	√										√		√	√	
Rule 20	√	√		√									√		√	√	
Rule 21	√	√											√	√	√	√	
Rule 22	√				√									√	√	√	√
Rule 23	√				√								√	√	√	√	
Rule 24		√		√	√		√	√							√		
Rule 25		√			√		√	√			√				√		
Rule 26	√		√		√		√						√		√		
Rule 27	√			√	√		√						√		√		
Rule 28	√				√		√			√			√		√		
Rule 29	√				√		√						√	√	√		
Rule 30		√		√	√			√			√				√		
Rule 31	√				√	√							√	√		√	

$RS_{0.796}$

**Feature**	**F_1**	**F_2**	**F_3**	**F_4**	**F_5**	**F_6**	**F_7**	**F_8**	**F_9**	**F_{10}**	**F_{11}**	**F_{12}**	**F_{13}**	**F_{14}**	**F_{15}**	**F_{16}**	**F_{17}**
Rule 1	√	√			√										√		
Rule 2		√			√			√	√						√		
Rule 3		√		√	√				√						√		
Rule 4		√			√				√				√		√		
Rule 5	√	√				√									√	√	
Rule 6		√			√			√							√	√	

Rule 7	√				√									√	√	√	
Rule 8		√			√	√		√							√		
Rule 9		√		√	√			√							√		
Rule 10		√			√			√						√	√		
Rule 11		√			√			√			√				√		
Rule 12		√			√			√					√		√		
Rule 13	√			√		√						√			√	√	
Rule 14	√					√						√		√	√	√	
Rule 15		√			√		√	√				√			√		
Rule 16		√				√	√	√							√	√	
Rule 17	√		√			√							√		√	√	
Rule 18	√			√		√								√	√	√	
Rule 19	√			√		√							√		√	√	
Rule 20	√					√							√	√	√	√	
Rule 21		√					√	√					√		√	√	
Rule 22	√				√		√						√		√	√	
Rule 23	√	√						√					√		√	√	
Rule 24	√	√	√										√		√	√	
Rule 25	√	√		√									√		√	√	
Rule 26	√	√											√	√	√	√	
Rule 27		√		√		√	√	√							√		
Rule 28		√				√	√	√							√		√
Rule 29			√		√	√	√	√							√		
Rule 30					√	√	√	√					√		√		
Rule 31	√	√				√		√							√		√
Rule 32	√	√				√		√						√	√		
Rule 33	√	√				√							√	√	√		
Rule 34	√				√		√	√					√		√		
Rule 35	√		√		√		√						√		√		
Rule 36	√			√	√		√							√	√		
Rule 37	√			√	√		√						√		√		
Rule 38	√				√		√			√			√		√		
Rule 39	√				√		√						√	√	√		
Rule 40	√		√		√	√								√		√	
Rule 41	√				√	√							√	√		√	

$RS_{0.784}$

**Feature**	**F_1**	**F_2**	**F_3**	**F_4**	**F_5**	**F_6**	**F_7**	**F_8**	**F_9**	**F_{10}**	**F_{11}**	**F_{12}**	**F_{13}**	**F_{14}**	**F_{15}**	**F_{16}**	**F_{17}**
Rule 1	√	√			√										√		
Rule 2		√			√			√	√						√		
Rule 3		√		√	√				√						√		
Rule 4	√	√				√									√	√	
Rule 5		√			√			√							√	√	
Rule 6	√				√									√	√	√	
Rule 7		√			√	√		√							√		

Rule 8		√		√	√			√							√		
Rule 9		√			√			√						√	√		
Rule 10		√			√			√			√				√		
Rule 11		√			√			√					√		√		
Rule 12	√			√		√						√			√	√	
Rule 13		√			√		√	√				√			√		
Rule 14		√			√		√		√				√		√		
Rule 15		√				√	√	√							√	√	
Rule 16	√			√		√								√	√	√	
Rule 17	√			√		√							√		√	√	
Rule 18	√					√							√	√	√	√	
Rule 19		√					√	√					√		√	√	
Rule 20	√				√		√						√		√	√	
Rule 21	√	√						√					√		√	√	
Rule 22	√	√	√										√		√	√	
Rule 23	√	√		√									√		√	√	
Rule 24	√	√											√	√	√	√	
Rule 25		√		√		√	√	√							√		
Rule 26		√				√	√	√							√		√
Rule 27			√		√	√	√	√							√		
Rule 28					√	√	√	√					√		√		
Rule 29	√	√				√		√							√		√
Rule 30	√	√				√		√						√	√		
Rule 31	√	√				√							√	√	√		
Rule 32	√				√		√	√					√		√		
Rule 33	√		√		√		√						√		√		
Rule 34	√			√	√		√						√		√		
Rule 35	√				√		√			√			√		√		
Rule 36	√				√		√						√	√	√		
Rule 37	√		√		√	√								√		√	
Rule 38	√				√	√							√	√		√	

List of features:

**Feature Index**	**Feature Names**
F_1	2nd nucleotide=A
F_2	4th nucleotide=C
F_3	6th nucleotide≠C
F_4	7th nucleotide≠U
F_5	9th nucleotide=C
F_6	17th nucleotide=A
F_7	18th nucleotide≠C
F_8	19th nucleotide=(A/U)
F_9	At least three (A/U)s in the seven nucleotides at the 3' end
F_{10}	No occurrences of four or more identical nucleotides in a row
F_{11}	No occurrences of G/C stretches of length 7 or longer

F_{12}	G/C content is between 35 and 60%
F_{13}	T_m is between 20 and 60°C
F_{14}	Binding energy of N16-N19 > -9 KCal/Mol
F_{15}	Binding energy of N16-N19 - binding energy of N1-N4 is between 0 and 1 KCal/Mol
F_{16}	Local folding potential (mean) ≥ -22.72 KCal/Mol
F_{17}	Target site is on CDS

Supplementary Table 7 Comparison of UPTR values among the 15 online siRNA design tools.

**Design Program**	**Institution/ Company**	n^T	n^T_{UPE}	n_{UPE}	**UPTR**
siDESIGN Center	Dharmacon, Inc.	33	8	19	4.152
siRNA Target Finder	GenScript Corp.	25	10	46	2.144
Imgenex sirna Designer	Imgenex Corp.	115	27	15	17.751
IDT RNAi Design (SciTools)	Integrated DNA Technologies, Inc.	27	6	7	8.453
BLOCK-iT RNAi Designer	Invitrogen Corp.	21	9	26	3.414
siSearch	Karolinska Institutet	16	7	47	1.469
SiMAX	MWG-Biotech, Inc.	162	71	66	10.609
BIOPREDsi	Novartis Institutes for BioMedical Research	13	6	21	2.818
Promega siRNA Target Designer	Promega Corp.	72	41	106	3.815
QIAGEN siRNA Design Tool	QIAGEN, Inc.	149	78	64	12.019
WI siRNA Selection Program	Whitehead Institute	87	38	24	15.615
Ambion siRNA Target Finder*	Ambion, Inc.	564	7	77	0.897
Jack Lin's siRNA Sequence Finder*	Cold Spring Harbor Laboratory	229	1	91	0.108

EMBOSS siRNA*	Institute Pasteur	765	28	370	0.746
SDS/MPI*	University of Hong Kong	642	8	89	0.886

n^T: number of predicted effective siRNA sites in the tested set (consisting of 1,014 sites);
n^T_{UPE}: number of uniquely predicted effective sites in the tested set;
n_{UPE}: number of uniquely predicted effective sites in the 10,000 randomly selected sites.

* High-coverage tools. The calculation of n^T_{UPE} for low-coverage tools was different from that for high-coverage tools (see text for details).

Supplementary Table 8 Significance of features pertinent to the considerations proposed in [32] for selecting effective siRNAs.

Considerations in [32]		Pertinent features	Significance
Sequence asymmetry of siRNA duplexes	The 5' end of guide strand is (A/U) enriched.	*17th nucleotide≠C*	**
		17th nucleotide≠G	**
		18th nucleotide=A	**
		18th nucleotide≠C	***
		19th nucleotide=A	***
		19th nucleotide≠G	***
		19th nucleotide=(A/U)	***
		At least five (A/U)s in the seven nucleotides at the 3' end	*
		At least three (A/U)s in the seven nucleotides at the 3' end	***
		At least three (A/U)s in the five nucleotides at the 3' end	***
	The 5' end of guide strand is less stable.	*Binding energy of N16-N19 > -9 KCal/Mol*	***
		Binding energy of N16-N19 ≥ binding energy of N1-N4	**
		Binding energy of N16-N19 – binding energy of N1-N4 is between 0 and 1 KCal/Mol	***
siRNA duplex stability	G/C content range between 30% and 52%	G/C content is between 30 and 52%	
	Devoid of internal repeats or palinedrome	*Folding energy of sense strand ≥ -5 KCal/Mol*	**
		T_m < 60°C	***
		T_m is between 20 and 60°C	***
	The center of the duplex have low internal stability.	Binding energy of N6-N11 ≥ -13 KCal/Mol	
Target accessibility		Anti-sense siRNA binding energy > -10 KCal/Mol	
		H-b index < 28.8	*
		Passes the repelling loop filter	
		Local free energy of the most stable structure ≥ -20.9 KCal/Mol	*
Sequence characteristics	U or A at position N19	19th nucleotide=A	***
		19th nucleotide≠G	***
		19th nucleotide=(A/U)	***

	G or C at position N1	1st nucleotide≠U	**
		1st nucleotide=G	**
		1st nucleotide=(G/C)	**
	A+U richness between position N13 and N19	At least five (A/U)s in the seven nucleotides at the 3' end	*
		At least three (A/U)s in the seven nucleotides at the 3' end	***
	A or U at position N10	10th nucleotide=(A/U)	
	U at position 3	3rd nucleotide≠U	
	Devoid of extended runs of altering G/C pairs or three G's	No occurrences of G/C stretches of length 7 or longer	***
		No occurrences of three or more identical nucleotides in a row	***

Features are shown in italic if they are pertinent to the considerations proposed in [32], but not specified explicitly.
Features with no marks: All three p-values are greater than 0.05.
Features marked by *: at least one of the three p-values is less than 0.05.
Features marked by **: at least two of the three p-values are less than 0.05.
Features marked by ***: (P_{wald} <0.01) and (P_{70}<0.01 or P_{90}<0.01).

References

1. Storey JD, Tibshirani R: **Statistical significance for genomewide studies**. *Proc Natl Acad Sci USA* 2003, **100**:9440-9445.
2. Takasaki S, Kotani S, Konagaya A: **An effective method for selecting siRNA target sequences in mammalian cells**. *Cell Cycle* 2004, **3**(6):790-795.
3. Amarzguioui M, Prydz H: **An algorithm for selection of functional siRNA sequences**. *Biochem Biophys Res Commun* 2004, **316**(4):1050-1058.
4. Reynolds A, Leake D, Boese Q, Scaringe S, Marshall WS, Khvorova A: **Rational siRNA design for RNA interference**. *Nat Biotechnol* 2004, **22**(3):326-330.
5. Hsieh AC, Bo R, Manola J, Vazquez F, Bare O, Khvorova A, Scaringe S, Sellers WR: **A library of siRNA duplexes targeting the phosphoinositide 3-kinase pathway: determinants of gene silencing for use in cell-based screens**. *Nucleic Acids Res* 2004, **32**(3):893-901.
6. Jagla B, Aulner N, Kelly PD, Song D, Volchuk A, Zatorski A, Shum D, Mayer T, De Angelis DA, Ouerfelli O *et al*: **Sequence characteristics of functional siRNAs**. *Rna* 2005, **11**(6):864-872.
7. Ui-Tei K, Naito Y, Takahashi F, Haraguchi T, Ohki-Hamazaki H, Juni A, Ueda R, Saigo K: **Guidelines for the selection of highly effective siRNA sequences for mammalian and chick RNA interference**. *Nucleic Acids Res* 2004, **32**(3):936-948.
8. Naito Y, Yamada T, Ui-Tei K, Morishita S, Saigo K: **siDirect: highly effective, target-specific siRNA design software for mammalian RNA interference**. *Nucleic Acids Res* 2004, **32**(Web Server issue):W124-129.
9. Wang L, Mu FY: **A Web-based design center for vector-based siRNA and siRNA cassette**. *Bioinformatics* 2004, **20**(11):1818-1820.
10. Yuan B, Latek R, Hossbach M, Tuschl T, Lewitter F: **siRNA Selection Server: an automated siRNA oligonucleotide prediction server**. *Nucleic Acids Res* 2004, **32**(Web Server issue):W130-134.
11. Cui W, Ning J, Naik UP, Duncan MK: **OptiRNAi, an RNAi design tool**. *Comput Methods Programs Biomed* 2004, **75**(1):67-73.
12. Ding Y, Chan CY, Lawrence CE: **Sfold web server for statistical folding and rational design of nucleic acids**. *Nucleic Acids Res* 2004, **32**(Web Server issue):W135-141.
13. Henschel A, Buchholz F, Habermann B: **DEQOR: a web-based tool for the design and quality control of siRNAs**. *Nucleic Acids Res* 2004, **32**(Web Server issue):W113-120.
14. Elbashir SM, Harborth J, Weber K, Tuschl T: **Analysis of gene function in somatic mammalian cells using small interfering RNAs**. *Methods* 2002, **26**(2):199-213.
15. Kumar R, Conklin DS, Mittal V: **High-throughput selection of effective RNAi probes for gene silencing**. *Genome Res* 2003, **13**(10):2333-2340.
16. Chalk AM, Wahlestedt C, Sonnhammer EL: **Improved and automated prediction of effective siRNA**. *Biochem Biophys Res Commun* 2004, **319**(1):264-274.

17. Khvorova A, Reynolds A, Jayasena SD: **Functional siRNAs and miRNAs exhibit strand bias**. *Cell* 2003, **115**(2):209-216.
18. Poliseno L, Evangelista M, Mercatanti A, Mariani L, Citti L, Rainaldi G: **The energy profiling of short interfering RNAs is highly predictive of their activity**. *Oligonucleotides* 2004, **14**(3):227-232.
19. Scherr M, Rossi JJ, Sczakiel G, Patzel V: **RNA accessibility prediction: a theoretical approach is consistent with experimental studies in cell extracts**. *Nucleic Acids Res* 2000, **28**(13):2455-2461.
20. Yiu SM, Wong PW, Lam TW, Mui YC, Kung HF, Lin M, Cheung YT: **Filtering of ineffective siRNAs and improved siRNA design tool**. *Bioinformatics* 2005, **21**(2):144-151.
21. Schubert S, Grunweller A, Erdmann VA, Kurreck J: **Local RNA target structure influences siRNA efficacy: systematic analysis of intentionally designed binding regions**. *J Mol Biol* 2005, **348**(4):883-893.
22. Luo KQ, Chang DC: **The gene-silencing efficiency of siRNA is strongly dependent on the local structure of mRNA at the targeted region**. *Biochem Biophys Res Commun* 2004, **318**(1):303-310.
23. McManus MT, Haines BB, Dillon CP, Whitehurst CE, van Parijs L, Chen J, Sharp PA: **Small interfering RNA-mediated gene silencing in T lymphocytes**. *J Immunol* 2002, **169**(10):5754-5760.
24. McManus MT, Sharp PA: **Gene silencing in mammals by small interfering RNAs**. *Nat Rev Genet* 2002, **3**(10):737-747.
25. Elmaagacli AH, Koldehoff M, Peceny R, Klein-Hitpass L, Ottinger H, Beelen DW, Opalka B: **WT1 and BCR-ABL specific small interfering RNA have additive effects in the induction of apoptosis in leukemic cells**. *Haematologica* 2005, **90**(3):326-334.
26. Nicholson LJ, Philippe M, Paine AJ, Mann DA, Dolphin CT: **RNA interference mediated in human primary cells via recombinant baculoviral vectors**. *Mol Ther* 2005, **11**(4):638-644.
27. Guan R, Tapang P, Leverson JD, Albert D, Giranda VL, Luo Y: **Small interfering RNA-mediated Polo-like kinase 1 depletion preferentially reduces the survival of p53-defective, oncogenic transformed cells and inhibits tumor growth in animals**. *Cancer Res* 2005, **65**(7):2698-2704.
28. Spankuch-Schmitt B, Bereiter-Hahn J, Kaufmann M, Strebhardt K: **Effect of RNA silencing of polo-like kinase-1 (PLK1) on apoptosis and spindle formation in human cancer cells**. *J Natl Cancer Inst* 2002, **94**(24):1863-1877.
29. Yuan J, Yan R, Kramer A, Eckerdt F, Roller M, Kaufmann M, Strebhardt K: **Cyclin B1 depletion inhibits proliferation and induces apoptosis in human tumor cells**. *Oncogene* 2004, **23**(34):5843-5852.
30. Atkinson PJ, Young KW, Ennion SJ, Kew JN, Nahorski SR, Challiss RA: **Altered Expression of Gq/11{alpha} Protein Shapes mGlu1 and mGlu5 Receptor-mediated Single Cell Inositol 1,4,5-trisphosphate and Ca2+ Signaling**. *Mol Pharmacol* 2005.
31. Sczakiel G, Homann M, Rittner K: **Computer-aided search for effective antisense RNA target sequences of the human immunodeficiency virus type 1**. *Antisense Res Dev* 1993, **3**(1):45-52.

32. Pei Y, Tuschl T: **On the art of identifying effective and specific siRNAs**. *Nat Methods* 2006, **3**(9):670-676.
